# Chromosome-level genome assembly, annotation, and phylogenomics of the gooseneck barnacle *Pollicipes pollicipes*

**DOI:** 10.1093/gigascience/giac021

**Published:** 2022-03-12

**Authors:** James P Bernot, Pavel Avdeyev, Anton Zamyatin, Niklas Dreyer, Nikita Alexeev, Marcos Pérez-Losada, Keith A Crandall

**Affiliations:** Computational Biology Institute, Milken Institute School of Public Health, The George Washington University, Washington, DC 20052, USA; Department of Invertebrate Zoology, National Museum of Natural History, Smithsonian Institution, Washington, DC 20012, USA; Computational Biology Institute, Milken Institute School of Public Health, The George Washington University, Washington, DC 20052, USA; Computer Technologies Laboratory, ITMO University, Saint-Petersburg 197101, Russia; Department of Life Science, National Taiwan Normal University, Taipei 106, Taiwan; Biodiversity Program, International Graduate Program, Academia Sinica, Taipei, Taiwan; Biodiversity Research Center, Academia Sinica, Taipei 115, Taiwan; Natural History Museum of Denmark, University of Copenhagen, Universitetsparken 15, DK-2100, Copenhagen, Denmark; Computer Technologies Laboratory, ITMO University, Saint-Petersburg 197101, Russia; Computational Biology Institute, Milken Institute School of Public Health, The George Washington University, Washington, DC 20052, USA; Department of Biostatistics & Bioinformatics, Milken Institute School of Public Health, The George Washington University, Washington, DC 20052, USA; CIBIO-InBIO, Centro de Investigação em Biodiversidade e Recursos Genéticos, Universidade do Porto, Campus Agrário de Vairão, Vairão 4485-661, Portugal; Computational Biology Institute, Milken Institute School of Public Health, The George Washington University, Washington, DC 20052, USA; Department of Invertebrate Zoology, National Museum of Natural History, Smithsonian Institution, Washington, DC 20012, USA; Department of Biostatistics & Bioinformatics, Milken Institute School of Public Health, The George Washington University, Washington, DC 20052, USA

**Keywords:** barnacle, genome, larval evolution, assembly, annotation, crustacea, phylogeny, Pollicipes

## Abstract

**Background:**

The barnacles are a group of >2,000 species that have fascinated biologists, including Darwin, for centuries. Their lifestyles are extremely diverse, from free-swimming larvae to sessile adults, and even root-like endoparasites. Barnacles also cause hundreds of millions of dollars of losses annually due to biofouling. However, genomic resources for crustaceans, and barnacles in particular, are lacking.

**Results:**

Using 62× Pacific Biosciences coverage, 189× Illumina whole-genome sequencing coverage, 203× HiC coverage, and 69× CHi-C coverage, we produced a chromosome-level genome assembly of the gooseneck barnacle *Pollicipes pollicipes*. The *P. pollicipes* genome is 770 Mb long and its assembly is one of the most contiguous and complete crustacean genomes available, with a scaffold N50 of 47 Mb and 90.5% of the BUSCO Arthropoda gene set. Using the genome annotation produced here along with transcriptomes of 13 other barnacle species, we completed phylogenomic analyses on a nearly 2 million amino acid alignment. Contrary to previous studies, our phylogenies suggest that the Pollicipedomorpha is monophyletic and sister to the Balanomorpha, which alters our understanding of barnacle larval evolution and suggests homoplasy in a number of naupliar characters. We also compared transcriptomes of *P. pollicipes* nauplius larvae and adults and found that nearly one-half of the genes in the genome are differentially expressed, highlighting the vastly different transcriptomes of larvae and adult gooseneck barnacles. Annotation of the genes with KEGG and GO terms reveals that these stages exhibit many differences including cuticle binding, chitin binding, microtubule motor activity, and membrane adhesion.

**Conclusion:**

This study provides high-quality genomic resources for a key group of crustaceans. This is especially valuable given the roles *P. pollicipes* plays in European fisheries, as a sentinel species for coastal ecosystems, and as a model for studying barnacle adhesion as well as its key position in the barnacle tree of life. A combination of genomic, phylogenetic, and transcriptomic analyses here provides valuable insights into the evolution and development of barnacles.

## Data Description

### Context

The Earth BioGenome Project (EBP) has the ambitious goal of sequencing a high-quality genome from each described eukaryotic species on the planet [1]. This goal can be especially difficult for invertebrate species because of the fundamental lack of available reference genomes [2]. The Pancrustacea (“Crustacea” + Hexapoda) is the most biologically diverse and species-rich animal taxon on the planet, containing >1.2 million described species. Even excluding the hyperdiverse insects, the Crustacea contains >60,000 described species [3], including numerous taxa of economic importance as food resources, fouling organisms, keystone species, and model organisms for biological research. Despite their importance, there is little genomic reference data available; <50 species have available genome sequences (42 species in NCBI), and only 7 assemblies approach chromosome-level contiguity.

**Figure 1:**
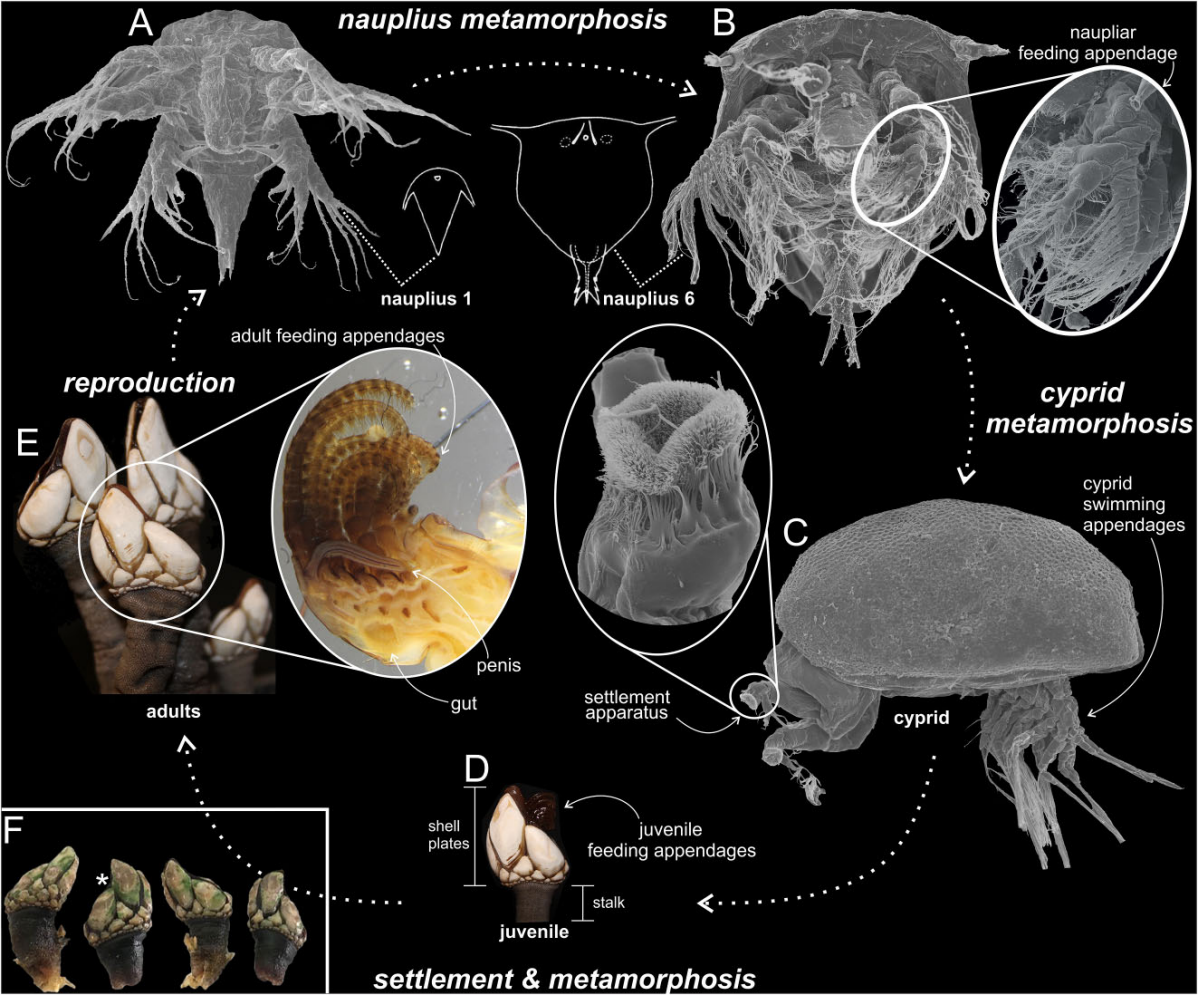
*Pollicipes pollicipes* life cycle. Note the fundamental structural differences among the life history stages. (A) Nauplius stage 1. (B) Nauplius stage 6. (C) Cyprid, insert showing magnified view of the third antennal segment used for permanent attachment to the substratum surface. (D) Juvenile adult. (E) Mature adult, insert showing a dissected adult specimen with 6 cirri or “feeding legs,” the penis, and the gut. (F) *P. pollicipes* voucher and genome sequencing specimens. Asterisk indicates genome hologenophore specimen (USNM 1622609).

The Thecostraca is a pancrustacean taxon containing the familiar and ubiquitous barnacles and a number of parasitic lineages comprising the Ascothoracida [4], Rhizocephala [5], and the enigmatic Facetotecta, for which adult stages have not yet been found [6]. The Cirripedia, or barnacles, are an almost entirely marine group of >2,000 species with a rich fossil record [7]. They display diverse morphological and biological characteristics including (i) free-swimming, plankton-feeding nauplius larvae (Fig. 1A and B), (ii) nonfeeding, settlement-larvae called cyprids (Fig. 1C), and (iii) sessile, shell-plated, suspension-feeding adults (Fig. 1D–F). Such diversity has made them model organisms in larval biology, morphology, sexual evolution, and intertidal ecology [7]. Barnacles have been the focus of evolutionary research since Darwin himself studied the group intently [8–11]. They are also notorious for fouling man-made objects, particularly ships and docks. Fouling barnacles are responsible for hundreds of millions of dollars in economic losses each year, primarily from fuel costs due to increased drag on ship hulls [12]; Schultz et al. [12] estimated that the US Navy alone overspends $180–$500 million each year owing to fouling.

**Figure 2:**
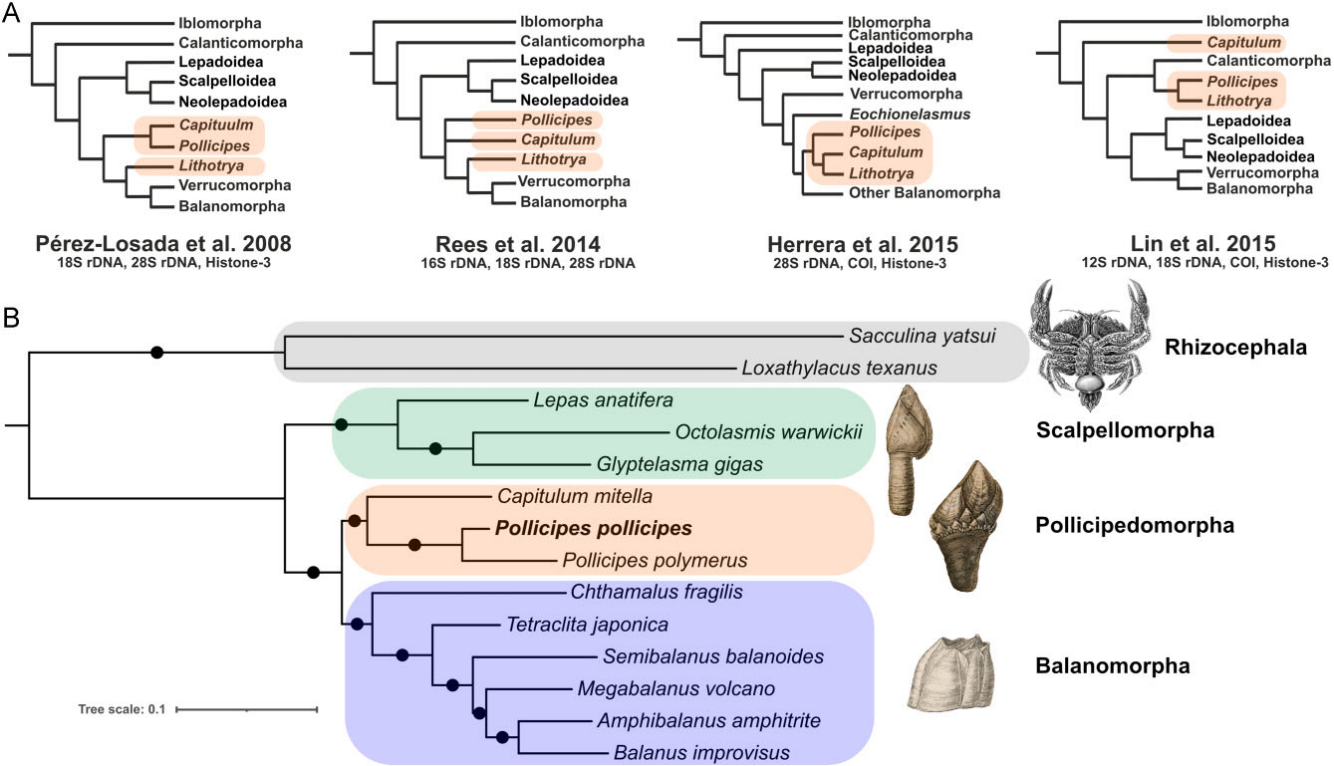
(A) Previous phylogenetic hypotheses on the position of Pollicipedomorpha taxa. (B) Phylogeny of the Cirripedia based on 5,734 protein-coding orthologs comprising 1,999,119 AA positions. The topology was identical across all analyses and all nodes received maximum support in all analyses (PP = 1, BS = 100%). Branch lengths for the partitioned ML analysis are shown. Illustrations from Darwin [8, 18] except for Rhizocephala, which is from Haeckel [19].

The gooseneck barnacle *Pollicipes pollicipes* (Gmelin, 1791 [in Gmelin, 1788–1792], NCBI:txid41117, marinespecies.org:taxname:106177) is a member of the Pollicipedomorpha (Thoracicalcarea), a new order [7] of stalked barnacles with a body encased by a wall of articulating, calcified shell plates atop an elongate peduncle (Fig. 1D). The order includes 4 genera (*Anelasma, Pollicipes, Capitulum*, and *Lithotrya*) that have a close phylogenetic affinity in molecular analyses. Many studies have placed them near the Balanomorpha and Verrucamorpha [13], but their phylogenetic position and even the monophyly of the order are still under debate—particularly in studies using adult and/or larval character matrices (Fig. 2A) [14–17].

Like in many invertebrate taxa, genomic resources for crustaceans are lacking, which has hindered the study of genome and phenotypic evolution, and the estimation of robust phylogenies [2]. The gooseneck barnacle *P. pollicipes* is a particularly good choice for genomic sequencing given its importance in European fisheries [20] and role as a sentinel species for coastal marine ecosystems [21]; *P. pollicipes* is also a model for studying barnacle adhesion mechanisms and engineering of new adhesive materials [22] and occupies a key phylogenetic position in the barnacle tree of life [7]. Furthermore, our barnacle genome project may represent an exemplar for future invertebrate genome sequencing, assembly, and annotation approaches in the EBP because it includes abundant, high-quality data, robust methods for assembly and annotation, appropriate vouchering of specimens used for genome sequencing, and metadata associated with the specimens—all with discoverable identifiers, allowing for an “extended specimen” [23, 24]. Therefore, the goal of this study is to sequence, assemble, and annotate the full genome of the gooseneck barnacle *P. pollicipes*. We think that this barnacle species is a great example of this important group of organisms and our genome approach regarding depth, diversity of data (extended specimen), linkage of data, and FAIRness [25–27] aligns well with the goals of the EBP.

## Methods and Materials

### Genomic sample collection

Samples for this study were collected by hand at 42 09 21.2 N, 8 50 59.2 W in Punta Meda, Nigran, Pontevedra, Spain (Supplementary Fig. S1), preserved in 95% ethanol, and stored at −80°C. Multiple individuals were collected to allow some to be used as vouchers (see Fig. 1), some for transcriptomics/proteomics, and some for genome sequencing. Voucher specimens are deposited in the US National Museum of Natural History, Smithsonian Institution, under accession Nos. USNM 1622609 (hologenophore) and USNM 1622610 (paragenophore lot).

### Larval development and life cycle

We reared larvae and adults of *P. pollicipes* and examined them with macro photography and light and scanning electron microscopy to provide an overview of the life cycle and key morphological features (Fig. 1). The adult specimens used for larval culturing and adult internal anatomy were collected in Quiberon Peninsula on the South coast of Brittany, France. Adult *P. pollicipes* groups or solitary individuals were carefully removed on a piece of substratum/rock using a hammer and chisel. The specimens were transported back to the laboratory wrapped in a wet towel and cultured at the University of Wales, Swansea, United Kingdom. The specimens were housed in laboratory aquaria with running seawater, and egg-lamellae were removed and cultured separately in filtered seawater and antibiotics (50 units penicillin and 0.05 mg streptomycin sulphate/mL water). Upon hatching from the eggs, ∼50 nauplius stage 1 specimens (Fig. 1A) were attracted with a light-source and fixed in 10% seawater-based formalin. The remaining nauplii were reared under a density of 5 × 10^2^ L^–1^ and fed with the flagellate algae *Isochrysis galbana* at a concentration of 100 × 10^4^ cells mL^–1^ at 20**°**C. Upon reaching nauplius stage 3, we switched diet to the large dinoflagellate *Prorocentrum micans*. Finally, 50 last-stage nauplii (nauplius 6) and 10 cyprid larvae were fixed in formalin for examination with scanning electron microscopy (SEM). One adult specimen was carefully dissected along its midline with a tweezers (Fig. 1E). Specimens for SEM-preparation were placed in filtered ddH_2_0 in small glass vessels. We changed the water thrice and left the larvae overnight. Larvae were then gradually dehydrated through an alcohol series (thrice each; 10–100%). Specimens were then critical point dried with liquid carbon dioxide, sputter coated with an alloy of platinum and palladium, placed on their lateral side on SEM-stubs with a hair brow wig taped to a stick, and finally photographed in a JEOL-JSM-6335F fitted with a field emission gun.

## Genome Sequencing

### Pacific Biosciences library preparation and sequencing

DNA was extracted from ethanol-preserved specimens following Dovetail Genomics protocols. DNA samples were quantified using a Qubit 2.0 Fluorometer (Life Technologies, Carlsbad, CA, USA). Fragments of ∼20 kb were selected for library preparation using SMRTbell Template Prep Kit 1.0 (Pacific Biosciences [PacBio], Menlo Park, CA, USA) following the manufacturer-recommended protocol. The pooled library was bound to polymerase using the Sequel Binding Kit 2.0 (PacBio) and loaded onto a PacBio Sequel (PacBio Sequel System, RRID:SCR_017989) using the MagBead Kit V2 (PacBio). Sequencing was performed on 14 PacBio Sequel SMRT cells using Instrument Control Software v5.0.0.6235, Primary Analysis Software v5.0.0.6236, and SMRT Link v5.0.0.6792. The resulting PacBio library contained 7.01 million reads with mean read length 6.84 kb, median 4.74 kb, and read length N50 12.34 kb, for a mean of 62.18× coverage (Supplementary Fig. S4). Statistics were gathered by the NanoStat tool from the NanoPack package [28].

### CHi-C library preparation and sequencing

Two capture Hi-C (CHi-C) libraries were prepared as described previously [29]. Briefly, for each library, ∼500 ng of high molecular weight genomic DNA (mean fragment length = 50 kb) was reconstituted into chromatin *in vitro* and fixed with formaldehyde. Fixed chromatin was digested with DpnII, the 5′ overhangs filled in with biotinylated nucleotides, and then free blunt ends were ligated. After ligation, cross-links were reversed and the DNA purified from protein. Purified DNA was treated to remove biotin that was not internal to ligated fragments. The DNA was then sheared to ∼350 bp mean fragment size and sequencing libraries were generated using NEBNext Ultra enzymes and Illumina-compatible adapters. Biotin-containing fragments were isolated using streptavidin beads before PCR enrichment of each library. The libraries were sequenced on an Illumina HiSeqX (Illumina HiSeq X Ten, RRID:SCR_016385). The number and length of read pairs produced for each library were as follows: 87 million, 2 × 150 bp for library 1 and 90 million, 2 × 150 bp for library 2. Together, these CHi-C libraries provided 68.96× physical coverage of the genome (1–50 kb).

### Dovetail HiC library preparation and sequencing

Two Dovetail HiC libraries were prepared in a similar manner as described previously [30]. Briefly, for each library, chromatin was fixed in place in the nucleus with formaldehyde and then extracted. Fixed chromatin was digested with DpnII, the 5′ overhangs filled in with biotinylated nucleotides, and free blunt ends were then ligated. After ligation, cross-links were reversed and the DNA purified from protein. Purified DNA was treated to remove biotin that was not internal to ligated fragments. The DNA was then sheared to ∼350 bp mean fragment size and sequencing libraries were generated using NEBNext Ultra enzymes and Illumina-compatible adapters. Biotin-containing fragments were isolated using streptavidin beads before PCR enrichment of each library. The libraries were sequenced on an Illumina HiSeqX. The number and length of read pairs produced for each library were as follows: 262 million, 2 × 150 bp for library 1 and 260 million, 2 × 150 bp for library 2. Together, these Dovetail HiC library reads provided 203.37× physical coverage of the genome (1–50 kb).

### Illumina library preparation and sequencing

Approximately 10 µL of the remaining DNA extracted as described above for PacBio sequencing was used for Illumina short-read sequencing. DNA samples were quantified using a Qubit 2.0 Fluorometer and libraries prepared using the standard Illumina DNA Prep protocol. The 10 µL of high molecular weight DNA were added to a PCR plate, combined with 20 µL of nuclease-free water for a total volume of 30 µL, vortexed, and combined with the tagmentation master mix, sealed, and placed on a thermocycler (55°C for 15 minutes, held at 10°C). Beads were resuspended by adding 10 µL of Tagment Stop Buffer, placed on a magnetic stand for 3 minutes, and the supernatant removed and discarded. The sample was washed twice with 100 µL Tagment Wash Buffer, allowing the sample to clear on the magnetic stand for 3 minutes each time. Another 100 µL of Tagment Wash Buffer was added and the tagmented DNA underwent limited PCR amplification to add dual index adapters (i7 and i5) following standard Illumina protocols. The libraries were then cleaned using the standard Illumina DNA double-sided bead purification procedure. The final libraries were used for 150 bp paired-end sequencing using a NextSeq High Output 300 cycle kit on an Illumina NextSeq 500 at the George Washington University Genomics Core.

Quality control showed that the Illumina library contains 1,043.2 million sequences (502 million PE reads) with mean duplication 26.6% and 145 bp mean read length. To exclude possible sequencing errors and eliminate phiX contamination, we filtered the library with quality cut-off 10 using the DADA2 pipeline [31]. The filtered library contained 904.6 million sequences with 25.3% read duplication.

### Genome Assembly

The genome assembly pipeline is shown in Supplementary Fig. S2. The initial genome assembly was performed using FALCON (FALCON, RRID:SCR_018804) [32] v1.8.8 [33]. First, 59.9× whole-genome, single-molecule, real-time sequencing (SMRT) data were used as input to the traditional FALCON pipeline using a length cut-off that corresponds to 50× coverage of data during the initial error-correcting stage. This resulted in 4 million error-corrected reads with an N50 read length equal to 9 kb covering 46.8× of the genome. The error-corrected reads were processed by the overlap portion of the FALCON pipeline. The aligned reads were assembled in the third stage of FALCON into 18,083 contigs. Finally, the assembly was polished with the Arrow algorithm from SMRT Link 5.0.1 using the original raw reads. The obtained assembly was assessed by QUAST-LG [34] and BUSCO v5.2.2 (BUSCO, RRID:SCR_015008) [35]. For the AUGUSTUS (v3.2) (Augustus, RRID:SCR_008417) tool [36] in the BUSCO pipeline, we used a gene model pre-trained on the *Drosophila melanogaster* genome.

### Mitochondrial genome assembly

We assembled the *P. pollicipes* mitochondrial genome using a modified version of the Vertebrate Genome Project mitoassembly pipeline [37]. Briefly, we aligned all PacBio reads to the *P. polymerus* mitochondrial genome (NCBI Reference Sequence: NC_005936.1) [38] using BlasR (BLASR, RRID:SCR_000764) [39] v5.3.3 [40]. We then used CANU (Canu, RRID:SCR_015880) [41] v2.0 to assemble 2,388 extracted reads into a single circular contig 27.8 kb long. The resulting contigs were aligned against the *P. polymerus* mitochondrial genome and itself (see Supplementary Fig. S8). Dot plots indicated the cyclic DNA repetitiveness, with ∼1.9 copies of the full mitochondrial genome present in the contig. We trimmed the contig to keep a single copy of the mitochondrial genome. The obtained sequence was further polished with ∼6,000× Illumina coverage using Pilon (Pilon, RRID:SCR_014731) [42] v1.23 [43]. Pilon confirmed 99.9% nucleotide bases and fixed 9 insertions, 2 deletions, and 1 single-nucleotide polymorphism.

### Genome scaffolding

The initial Falcon assembly, CHi-C reads, and Hi-C reads were used as input in HiRise, a Dovetail Genomics software pipeline for using proximity ligation data to scaffold genome assemblies. An iterative analysis was conducted. First, CHi-C library sequences were aligned to the Falcon draft assembly using a modified SNAP [44] read mapper [45]. The separations of CHi-C read pairs mapped within draft scaffolds were analyzed by HiRise to produce a likelihood model for genomic distance between read pairs. The model was used to identify and break suspected incorrect joinings, to score prospective joins, and to make joins above a threshold. The resulting assembly contained 8,768 contigs of 906 Mb total length with an N50 of 660.8 kb. After aligning and scaffolding CHi-C data, HiC library sequences were aligned and scaffolded with the same method. At the last stage, the original PacBio long reads were used to close gaps between contigs. Supplementary Fig. S5 shows the resulting contact heat map for the resulting assembly of the *P. pollicipes* genome produced by HiRise.

## Genome Curation

### Haplotype filtration

Given the higher rate of core gene duplications (21.1% of Arthropoda genes; Table 2), we suspected the presence of haplotypes in the scaffolded assembly. Therefore, we classified all contigs with PurgeHaplotigs [46] v1.0.0 [47] into primary contigs, haplotigs, repeat contigs, and assembly artifacts based on the read-depth analysis as follows. Read-depth histograms were produced for the draft assembly (see Supplementary Fig. S7). In each read-depth histogram, we chose 3 cut-offs to capture 2 peaks of the bimodal distribution that correspond to haploid and diploid levels of coverage. The first read-depth peak resulted from the duplicated regions and corresponds to the “haploid” level of coverage. The second read-depth peak resulted from regions that are haplotype-fused and corresponded to the “diploid” level of coverage. We removed everything that was not classified as primary and repeated contigs from the assembly. We additionally generated an assembly where repeat contigs were removed. The number of contigs decreased to 1,254 with repeats and 570 without repeats. The rate of duplicated genes was 12.8% after filtration (see Supplementary Tables S1 and S2). We kept contigs containing repeats in the final assembly because they may represent regions of interest for further research (e.g., transposable elements).

### Filtering for contaminants

We screened the barnacle assembly for contamination because the genomic DNA samples came from wild barnacle specimens that may have other species in and on them. The contamination search was first attempted using Kraken 2 (Kraken, RRID:SCR_005484) [48, 49] against the complete Kraken database. Because the Kraken database does not include a nearby reference genome for barnacles, Kraken 2 unclassified the majority of scaffolds and, more problematically, classified them into unrelated taxa including vertebrates, fungi, and plants. Most of these classifications were unlikely (e.g., vertebrates and terrestrial plants present on barnacles), especially given that these results were not confirmed later by manual BLAST searches. This issue was likely caused by the absence of a close reference, and, as a result, each scaffold was classified with minor identity by the *k*-mer approach used in Kraken.

Therefore, we used a modified version of a method for removing human DNA contamination in bacterial genome assemblies recently proposed [50]. Briefly, the original method divides genome scaffolds into overlapping subreads and maps each subread to the reference database using NCBI BLAST [51]. We partitioned our barnacle reads into 10-kb pseudoreads with 5 kb overlap. Pseudoreads were then aligned to the NCBI nucleotide database using MegaBLAST [52] with custom parameters. In contrast to the original strategy, where the authors used the NCBI RefSeq database [53], we mapped against the NCBI nucleotide database because there are not many reference assemblies for crustaceans and we wanted all accessions to the nearest sequenced organisms.

We performed 2 levels of analysis. First, we analyzed hits with an arbitrary length of alignment and e-value <0.01. Second, we analyzed hits with length of alignment ≥500 bp (minimal length of the PacBio error-corrected reads) and e-value <1e−50. The former method showed more homologous hits, while the latter method showed more hits with contamination. Plots with the color representation of each scaffold subreads taxonomy classification were built, and contaminated scaffolds were identified. For each pseudoscaffold classified as a contaminant, the full scaffold from which it came was then aligned to the NCBI nucleotide database with MegaBLAST to confirm bacterial contamination and when confirmed, the contaminated scaffolds were removed from the assembly.

### Polishing

The filtered assembly of long reads from PacBio reads are prone to insertion and deletion errors, which usually are corrected by polishing. Our assembly was polished using Illumina whole-genome sequencing (WGS) reads. Three rounds of polishing were completed using Pilon (v1.23) (-fix-all) to produce the final assembly. Pilon (Pilon, RRID:SCR_014731) confirmed 83.9% of the assembly with Illumina reads alignment at the first round and 85.35% at the third round. Supplementary Table S3 provides detailed statistics of individual base, indels, and gap corrections for each round. After 3 rounds, the percentage of complete Arthropoda BUSCO genes equaled 90.5%.

### Genome size estimation

*P. pollicipes* genome size was estimated from the final assembly length and by *k*-mer analysis (*k* = 21) of the Illumina genomic DNA pair-end reads for validation. The frequency distribution of 21-mers was computed by Jellyfish (Jellyfish, RRID:SCR_005491) [54] v2.3.0 [55]. Supplementary Fig. S3 shows bimodal frequency distribution of 21-mers. The first and second peaks in the distribution correspond to 21-mers from heterozygous and homozygous regions, respectively. The mean 21-mer coverage was 62 for heterozygous regions and 124 for homozygous ones. We approximated the frequency distribution with 2 normal distributions with means 62 (SD 14) and 124 (SD 20) to estimate the monoploid genome size.

### Genome annotation

Genome annotation was performed with the NCBI Eukaryotic Genome Annotation Pipeline [56] v8.5. Briefly, masking of repeats was first attempted with RepeatMasker (RepeatMasker, RRID:SCR_012954), but owing to the lack of a comprehensive repeat library, repeats were masked with WindowMasker [57]. Available transcripts, RNA-Seq (Supplementary Table S4), and protein data from RefSeq [58] were aligned to the masked genome using BLAST followed by refinement with SPLIGN [59]. Protein, transcript, and RNA-Seq alignments were used as input for 2 rounds of gene prediction with Gnomon [60]. The final set of annotated features was built by evaluating the known RefSeq transcripts, the features projected from curated RefSeq genomic alignments, and the most highly supported models predicted by Gnomon, respectively, at each locus. Protein naming, determination of locus type, and GeneID assignment followed the NCBI Eukaryotic Genome Annotation Pipeline standards.

### Transcriptome assembly

All transcriptomes were assembled *de novo* as follows. Raw reads were downloaded from NCBI SRA [61], read quality was assessed using FastQC (FastQC, RRID:SCR_014583) [62] v0.11.8, reads were subjected to quality and adapter trimming using Trimmomatic (Trimmomatic, RRID:SCR_011848) [63] v0.33 (ILLUMINACLIP: TruSeq3-PE-2.fa:2:30:10 LEADING:3 TRAILING:3 SLIDINGWINDOW:4:15 MINLEN:50) [64], and quality trimming and adapter removal was confirmed using FastQC again after trimming. Trimmed reads were error-corrected using Rcorrector [65] v1.0.4 [66] with default settings. Error-corrected reads were assembled using Trinity (Trinity, RRID:SCR_013048) [67] v2.10.0 [68, 69] under default parameters except that minimum *k*-mer coverage was set to 2. Assembled contigs were translated to amino acid (AA) sequences using TransDecoder (TransDecoder, RRID:SCR_017647) [70] v5.2.0 [68] with open reading frames identified using default parameters.

### Ortholog identification

Orthologs were identified using the phylogenetic approach described by Yang and Smith [71] and the scripts provided in that study. First, the predicted proteins from the transcriptomes had redundancy in AA sequence reduced using CD-HIT (CD-HIT, RRID:SCR_007105) [72] v4.6.8 [73, 74] with a 99% similarity threshold. Then the transcriptomes and genome were subjected to an all-by-all BLAST search (-max_target_seqs 1000 -evalue 10) and the resulting BLAST output was filtered for a hit fraction ≥0.4. Filtered BLAST hits were further clustered using MCL [75] v14.137 [76] with a −log E-value cut-off set to 5 and an I-value of 1.4 to identify homologous protein sequences. Fasta files were written from the MCL output using write_fasta_files_from_mcl.py.

Each cluster of homologs was then aligned individually with MAFFT [77] v7.427 (–genafpair–maxiterate 1000 if <1,000 sequences; –auto if >1,000 sequences) [78], trimmed using phyutility (minimum column occupancy = 0.1) [79], and trees were built using either RAxML (RAxML, RRID:SCR_006086) [80] v8.2.12 [81] under the model “PROTGAMMALG” for clusters with <1,000 sequences, or FastTree (FastTree, RRID:SCR_015501) [82] v2.1.10 [83] under the model “-lg” for clusters >1,000 sequences. The resulting trees may contain branches representing paralogs or misassembled contigs, so they were filtered using the following 3 methods from Yang and Smith [71]. First, divergent sequences were removed from clusters if a terminal branch was longer than 0.75 or >10× longer than its sister using trim_tips.py, following the parameters used for the MIL dataset, a taxon of similar age, in Yang and Smith [71]. Next, if monophyletic or paraphyletic tips from the same taxa were present in a tree, only the sequence with the highest number of non-ambiguous characters in the trimmed alignment was kept and the rest removed following previously published methods [71, 84]. Last, potential deep paralogs were removed using cut_long_internal_branches.py with an internal branch length cut-off of 1.5 and a minimum number of taxa of 7 (i.e., 50%). Fasta files were written from the trimmed trees and alignments and the entire process of aligning, trimming alignments, building trees, and removing paralogs and long branches was repeated. After the second round of refinement, the trees were called homolog trees and were further pruned to call orthologs.

Orthologs were called using the maximum inclusion method [71, 84, 85]. After pruning the homolog trees to identify maximum inclusion orthologs, the remaining subtrees might still have contained terminal taxa subtended by long branches as a result of the subtree trimming method [71]. To account for this, the trees were trimmed once more using a range of permissive-to-strict branch length trimming parameters, referred to from here on as permissive and strict branch trimming, with relative branch lengths of 10× and absolute branch lengths of 0.4 or 0.3 at the permissive and strict levels, respectively. Because of the large number of orthologs retrieved from both trimming parameters, the orthologs resulting from the strict, more conservative, trimming were used for downstream phylogenetic analyses.

### Phylogenomics

The final orthologs were aligned individually using MAFFT (MAFFT, RRID:SCR_011811) and trimmed using Gblocks (Gblocks, RRID:SCR_015945) [86] v0.91b [87] following the same parameters detailed above. The Gblocks-trimmed alignments were then concatenated using concatenate_matrices.py with a minimum length of 100 AA and a minimum taxon cut-off of 7 (50%). Phylogenetic analyses were completed using concatenation and coalescent methods. Concatenated analyses were done with a maximum likelihood (ML) partitioned analysis and with an ML mixture model. The partitioned, concatenated analysis was carried out using IQTree [88] v1.6.11 [89]. Partitions and models of evolution for each partition were selected using the fast relaxed-hierarchical clustering algorithm (-rclusterf) [90], followed by tree building with 1,000 ultrafast bootstrap (BS) pseudoreplicates [91]. Mixture models were also used for ML tree search because they account for among-site variation in AA propensities and thus are less prone to artifacts like long branch attraction [92–94]. For the ML mixture model tree search, the c20 mixture model implemented in IQTREE v1.6.11 (-m LG+C20+F+G) was used to build a starting tree, and the resulting tree was used as a guide tree for a c60 posterior mean site frequency model [95] (-m LG+C60+F+G -ft) with 100 BS pseudoreplicates. For the coalescent approach, individual gene trees were built for each ortholog using IQTree and the substitution model of best fit (-mfp) with 1,000 rapid BS pseudoreplicates. A species tree was then estimated by using all gene trees as input in ASTRAL [96] v5.6.3 [97].

### Differential gene expression

Raw RNA-Seq reads for 2 replicates each of *P. pollicipes* nauplii and adults were downloaded from NCBI (Supplementary Table S4) [98]. Reads were subjected to error correction with Rcorrector [66] using default settings and aligned to the *P. pollicipes* genome assembly GCA_011947565.2 downloaded from NCBI using HISAT2 (HISAT2, RRID:SCR_015530) [99] v2.1 [100]. A GTF file was generated using GffRead (gffread, RRID:SCR_018965) [101] v0.12.7 [102] and the *P. pollicipes* genome GFF file from NCBI. Read counts were generated using featureCounts in the Subread package [103] v2.0.1 (-t exon -g gene_name) [104]. Differential gene expression analysis was performed using DESeq2 (DESeq2, RRID:SCR_015687) [105] v1.34 [106] with default settings. Results were considered significant when *P* < 0.05 after false discovery rate (FDR) correction (*q* < 0.05). A log_2_ fold-change of ≥2 was used to further filter differentially expressed genes (DEGs). To estimate the number of genes with expression unique to, or shared between, the nauplius and adult stages, FPKM was calculated for each sample using DESeq2. Genes with FPKM <0.5 were counted as not expressed owing to the presence of transcriptional noise in RNA-Seq datasets [107,108].

To classify the DEGs into functional categories, the AA sequences of all genes were mapped to GO terms [109, 110] by identifying pfam domains [111] with InterProScan (InterProScan, RRID:SCR_005829) [112] 5.46–81.0 [113]. Because DESeq2 maps reads to genes and not constituent isoforms, when >1 isoform was present for a gene, the longest isoform was used for the functional mapping of DEGs. Enrichment analysis of Gene Ontology (GO) terms was carried out with topGO (topGO, RRID:SCR_014798) [114] v2.44 [115] (nodeSize = 5) by comparing GO terms from DEGs to GO terms of all expressed protein-coding genes in the genome, and significance was determined using Fisher exact test (*q* < 0.05). To further identify functional categories and pathways, DEGs were mapped to KEGG orthologs and pathways [116] using KofamKOALA [117] v2021-10-03 with an E-value cut-off of 0.01 [118].

## Results

### Larval development and life cycle

Here we document the life cycle of *P. pollicipes*. It is not the purpose of this study to describe all morphological changes seen during larval development, nor was it our intention to report on the setation formulae of naupliar stages. Briefly, the first nauplius stage (N1) hatches from the egg and is nonfeeding (lecithotrophic), relying on yolk stores (Fig. 1A). This stage is morphologically reduced compared to the later, more complex naupliar instars (Fig. 1A and B). Shortly after hatching, the nauplius undergoes a series of 5 molts of feeding instars, finally arriving at the N6 (Fig. 1B). The nauplius increases in size by nearly 3× during development, with the N1 growing from 0.21–0.24 mm to an N6 of 0.56–0.62 mm (N = 50 per stage). After 9–11 days post-hatching, the N6 molts into the next larval phase, the cyprid, which is a stage specialized for settlement (Fig. 1C). Using modified antennules equipped with a battery of sensory and attachment structures (Fig. 1C), the cyprid walks over substrates in a bipedal, exploratory manner. After 2–3 days of various probing behaviors, the cyprid commits to permanent settlement by releasing larval cement. Juvenile metamorphosis begins, followed by subsequent molting into the adult phase (Fig. 1D). Juveniles and adults grow rapidly from a ∼0.5-mm cyprid to large stalked adults that can exceed 3 cm in length and shell plate widths >1 cm. Adults are often found in clusters of individuals (Fig. 1E). The life cycle is completed when hermaphroditic adults cross-fertilize via an extensible penis (Fig. 1E) and produce clutches of hundreds of eggs.

### Genome assembly

We sequenced the *P. pollicipes* genome with 62× PacBio coverage, 196× Illumina WGS coverage, 203× HiC coverage, and 69× capture Hi-C (CHi-C) coverage (Table 1). The total assembly length was 770 Mb with a scaffold N50 of 47 Mb, a scaffold L50 of 8, and the largest scaffold being 64 Mb (Table 2). More than 92% of the assembly length was composed of 17 large scaffolds (Supplementary Figs S5 and S6). Of the 1,066 genes in the arthropod BUSCO gene set [119, 120], 90.5% of them were assembled completely, 3.3% were fragmented, and 6.2% were missing from the assembly. Results from BUSCO analysis of the 978 conserved single-copy metazoan genes were similar, with 91.2% assembled completely, 4.4% fragmented, and 4.4% missing (Table 2). Using a modified version of genome contamination removal suggested in [50], we identified 62 of the scaffolds in the assembly as bacterial contaminants, which we then removed. All 62 contaminant scaffolds were relatively small and collectively comprised ∼7 Mb, or just <1% of the final assembly length. This method is highly effective at identifying sequences containing homogenous contaminant DNA, but it may be less effective in the presence of a small proportion of chimeric contamination. We also assembled the mitochondrial genome separately using the PacBio reads and Canu, and then polished the assembly with 6,000× Illumina coverage using Pilon. The final mitochondrial genome was 15,090 bp.

**Table 1:** Genome sequencing mean coverage based on raw data (prior to QA/QC) and genome size of 770 Mb

Data type	Raw data (bp)	Coverage (×)
PacBio	47,984,705,480	62
Illumina WGS	150,600,000,000	196
HiC	156,600,000,000	203
CHi-C	53,100,000,000	69

**Table 2:** *Pollicipes pollicipes* genome assembly statistics

Statistic	Value
Total assembly length (Mb)	770
GC (%)	52.3
Largest scaffold (bp)	64,043,775
Scaffold N50 (bp)	47,009,503
Scaffold N75 (bp)	37,696,644
Scaffold L50	8
Scaffold L75	12
No. contigs	1,254
Contig N50 (bp)	95,549
Contig N75 (bp)	22,233
Contig N90 (bp)	16,125
BUSCO Arthropoda (%)	
Complete	90.5
Single	69.4
Duplicated	21.1
Fragmented	3.3
Missing	6.2
BUSCO Metazoa (%)	
Complete	91.2
Single	67.8
Duplicate	23.4
Fragmented	4.4
Missing	4.4

To validate the genome size measured from the assembly length, we also estimated the genome with a *k*-mer analysis of Illumina WGS using Jellyfish. The estimated genome size from Jellyfish was 702 Mb, close to the total length of the resulting assembly (770 Mb), indicating that our assembly covers the majority of the genome well. However, there was a double peak in the distribution of *k*-mers in the Jellyfish estimate (Supplementary Fig. S7), which affects *k*-mer–based size estimates [121]. This bimodal distribution is typical of heterozygous genomes [122], which is unsurprising given the samples we sequenced were of non-inbred individuals from a large, wild population. As a result, the assembly length of 770 Mb was used to calculate coverage estimates.

We compared our newly generated *P. pollicipes* assembly with the 7 other available chromosome-level crustacean assemblies (*Caligus rogercresseyi, Daphnia carinata, Daphnia magna, Eriocheir sinensis, Paralithodes platypus, Tigriopus californicus, Tigriopus japonicus*) and the 3 other available barnacle genome assemblies (Table 3). Because BUSCO scores and contiguity statistics were not provided for all of these assemblies, we generated BUSCO reports and measured N50 and L50 for each for comparative purposes.

**Table 3:** Comparison of barnacle genomes and chromosome-level crustacean genome assemblies

Contiguity	Taxon	Species	Assembly	Genome size (Mb)	Scaffold N50	Scaffold L50	Arthropod BUSCO (%)	Reference
**Chromosome**	**Thecostraca**	***Pollicipes pollicipes***	**GCA_011947565.2**	**770**	**47,009,503**	**8**	**90.5**	**This study**
Scaffold	Thecostraca	*Amphibalanus amphitrite*	GCA_009805615.1	613	458,238	415	92.4	[123]
Scaffold	Thecostraca	*Semibalanus balanoides*	GCA_014673585.1	482^1^	56,726	1,896	56.4	NCBI
Contig	Thecostraca	*Semibalanus balanoides*	GCA_003709985.1	101^1^	1,475	24,797	14.5	NCBI
Chromosome	Branchiopoda	*Daphnia carinata*	GCA_013167095.1	132	8,418,570	7	98.6	NCBI
Chromosome	Branchiopoda	*Daphnia magna*	GCA_003990815.1	123	10,124,675	6	98.0	[124]
Chromosome	Copepoda	*Caligus rogercresseyi*	GCA_013387185.1	478	27,802,916	8	61.5	NCBI
Chromosome	Copepoda	*Tigriopus californicus*	GCA_007210705.1	191	15,806,032	6	93.5	[125]
Chromosome	Copepoda	*Tigriopus japonicus*	GCA_010645155.1	197	10,654,335	8	94.1	[126]
Chromosome	Decapoda	*Eriocheir sinensis*	GCA_013436485.1	1,272	17,608,299	30	92.6	[127]
Chromosome	Decapoda	*Paralithodes platypus*	GCA_013283005.1	4,805	51,153,954	39	81.4	[128]

1 The size of this assembly is much shorter than the estimated size of the haploid genome (1,300–1,600 Mb).

Genome annotation with the NCBI Eukaryotic Genome Annotation Pipeline identified 31,804 transcripts and 25,694 genes. Of the genes, 20,444 were protein coding, 4,220 were noncoding, and 1,030 were pseudogenes. The 24,664 genes (excluding pseudogenes) had a mean length of 13,244 bp and a median length of 6,980 bp. A mean of 1.3 transcripts were identified for each gene, with a mean of 7.48 exons per transcript. Exons had a mean length of 241 bp while introns averaged 2,077 bp. RepeatMasker identified 3.2% of the genome as repetitive sequences, but a comprehensive repeat library is not available for barnacles, especially not for gooseneck barnacles, and nearly all repeats were classified as simple repeats or low-complexity repeats. To avoid reliance on a repeat library, WindowMasker was used and masked 18.5% of the genome prior to annotation.

### Phylogenomics

**Figure 3:**
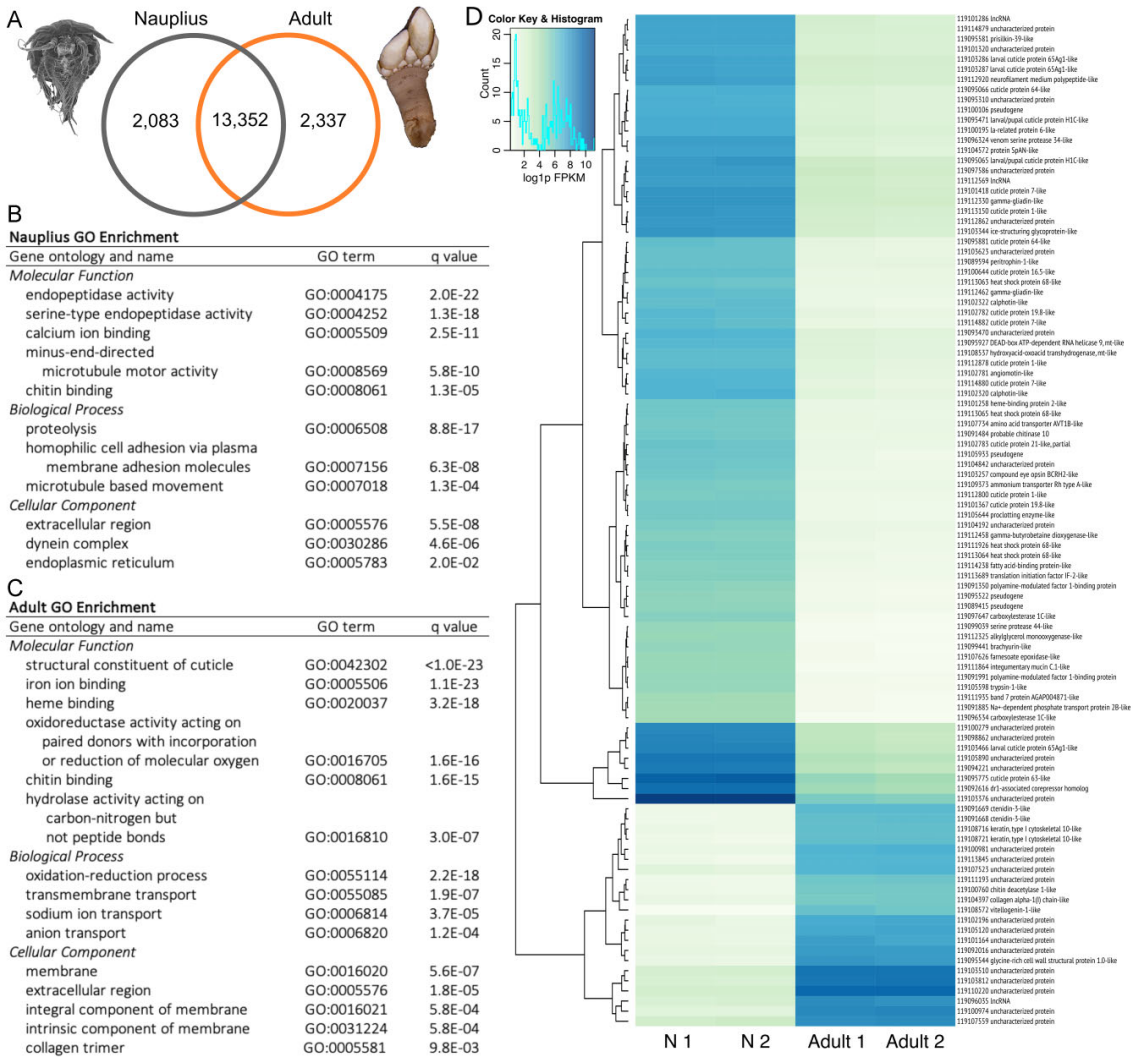
: (A) Venn diagram of genes expressed in each life stage. (B) Summary of most significant, enriched GO terms in nauplius DEGs accounting for nested GO terms. (C) Summary of most significant, enriched GO terms in adult DEGs accounting for nested GO terms. (D) Heat map of top 100 DEGs including gene IDs and annotations, clustered according to expression on the y-axis. N 1 = nauplius 1, N 2 = nauplius 2.

A phylogenetic analysis of selected barnacles was performed using the *P. pollicipes* genome and transcriptomes from 13 other barnacle species (Table 4). In total, 5,734 orthologs of ≥100 AA were identified, which produced a concatenated alignment 1,999,119 AA long. The Rhizocephala was selected as the outgroup following previous studies [5, 7, 129, 130]. All concatenated and coalescent-based phylogenetic analyses had identical topologies, and each tree had maximum support values (posterior probability [PP] = 1, BS = 100%) for all nodes. The tree from the partitioned ML analysis is shown in Fig. 3D.

**Table 4:** Taxa and orthologs used in phylogenetic analyses

Taxon	No. orthologs (%)	AA positions	Accession No.
*Pollicipes polymerus*	2,137 (37)	314,239	SRR10034703
*Capitulum mitella*	2,220 (39)	475,439	SRR10012027
*Loxathylacus texanus*	2,216 (39)	534,367	SRR5140130
*Semibalanus balanoides*	3,685 (64)	678,927	SRR5140144
*Sacculina yatsui*	2,238 (39)	783,829	DRR169034, DRR169035, DRR169036
*Balanus improvisus*	4,232 (74)	931,663	SRR8775110
*Tetraclita japonica*	4,571 (80)	1,137,491	SRR426837
*Chthmalus fragilis*	4,833 (84)	1,188,730	SRR4113502
*Lepas anatifera*	4,919 (86)	1,653,588	SRR6818896
*Pollicipes pollicipes*	5,092 (89)	1,742,039	GCA_011947565.2
*Megabalanus volcano*	5,363 (94)	1,777,080	SRR5091879, SRR5091880
*Octolasmis warwickii*	5,161 (90)	1,790,600	SRR10527303
*Glyptelasma gigas*	5,221 (91)	1,790,661	SRR10523768
*Amphibalanus amphitrite*	5,385 (94)	1,807,445	SRR10034703

### Differential gene expression of nauplii and adults

On average, 76.4% of RNA-Seq reads per sample aligned to the *Pollicipes* genome. The total aligned reads per sample are as follows: larva1 = 26.6 million, larva2 = 12.5 million, adult1 = 34.2 million, adult2 = 26.1 million. In total, reads aligned to 23,075 genes from the assembly. After removing genes with very low expression (<0.5 FPKM) to filter out transcriptional noise, we observed 2,083 genes expressed only in the nauplius stage, 2,337 unique to the adult stage, and 13,352 genes were expressed in both stages (Fig. 3A). However, many of the shared genes differed in their expression level. Of the 24,668 genes in the *P. pollicipes* genome, 11,846 were DEGs between the nauplius and adult stages after FDR correction (*q* < 0.05). A similar proportion of the DEGs were overexpressed in each stage (5,870 in nauplii, 5,976 in adults). To further filter the DEGs, a log_2_ fold-change > 2 cut-off was applied, which resulted in 5,189 DEGs (2,400 overexpressed in nauplii, 2,789 overexpressed in adults). Of these DEGs, 91 and 112 in nauplii and adults, respectively, were classified as pseudogenes in the genome annotation, while 332 genes in nauplii and 148 genes in adults were long non-coding RNAs (lncRNAs).

To explore the functions of DEGs, they were further mapped to GO terms and KEGG orthologs and pathways. We attempted to map all expressed protein-coding genes to GO terms with pfam and annotated 51% (10,436/20,443) of all genes with GO terms, including 51.5% (2,321/4,507) of the most highly DEGs. Figure 3B shows the most significant, enriched GO terms in the nauplius stage accounting for the nesting of GO terms (see full results in Supplementary Table S5, Supplementary Figs S9–S11). DEGs in nauplii were enriched for molecular motor activity, peptidases, homophilic cell adhesion via membrane-bound proteins, and chitin-binding proteins, among others (Fig. 3B). Overall, secretory proteins were enriched in nauplii relative to adults. Results of the GO enrichment analysis in the adult stage are likewise provided (Fig. 3C, Supplementary Table S6, Supplementary Figs S12–S14). DEGs in adults were enriched for structural components of cuticle, iron/heme binding, oxioreductase and hydrolase activity, sodium and anion transport, and chitin binding (Fig. 3C). Enrichment for membrane-bound proteins was highly significant (*P* = 5.6E−10) in adults but not in nauplii.

Functions of DEGs were also examined using KEGG orthologs and KEGG pathways. Of the protein-coding DEGs, 81.8% (3,685/4,507) were assigned to KEGG orthologs using KofamKOALA and these mapped to 335 KEGG pathways (Supplementary Tables S7 and S8). The most frequently identified KEGG pathways assigned to DEGs overexpressed in the nauplius with the percent of annotated DEGs followed by the count of DEGs in parentheses were represented as follows: metabolism (6.6%, 104), biosynthesis of secondary metabolites (4.8%, 76), transport and catabolism (2.8%, 45), signal transduction (2.4%, 38), carbohydrate metabolism (2.3%, 36), glycan biosynthesis and metabolism (2.1%, 33), amino acid metabolism (1.6%, 25), and transcription and translation (1.5%, 23). The most frequently identified KEGG pathways assigned to DEGs in adults were metabolism (11%, 229), carbohydrate metabolism (3.7%, 78), signal transduction (3.7%, 78), biosynthesis of secondary metabolites (3.6%, 76), amino acid metabolism (2.8%, 59), biosynthesis of cofactors (2.6%, 54), metabolism of cofactors and vitamins (2.3%, 49), and lipid metabolism (2.2%, 46).

The functional annotation from the NCBI annotation pipeline was examined manually for the top 100 most differentially expressed genes (*q* < 1E−10, log_2_ fold-change > 7) (Fig. 3D). The most common annotations for these DEGs were as follows: 18 were cuticle proteins (all upregulated in the nauplius); 14 were various enzymes (e.g., proteases, deacetylases, oxygenases, 1 RNA helicase); 4 were heat shock proteins; 4 were involved with chitin modifications (deactylase, chitinase, prisilkin-39-like, peritrophin-1-like); 4 were pseudogenes; 3 were involved with vision pathways (2 calphotins, 1 opsin, all upregulated in the nauplius); 3 were lncRNAs; and the remainder had miscellaneous functions.

## Discussion

### Genome assembly

We assembled a highly contiguous genome for the stalked barnacle *P. pollicipes*. More than 92% of the assembly length was composed of 17 large scaffolds, which likely represent 16 or 17 chromosomes or chromosome arms (Supplementary Figs S5 and S6). The smaller 17th scaffold may represent a small chromosome or the arm of a chromosome that remained unlinked. It is difficult to confirm a chromosome count for *P. pollicipes* because the number of chromosomes has not yet been recorded for this species, and chromosomal counts in crustaceans are highly variable [131]. Nonetheless, the scaffolds assembled here are as long as or longer than most chromosome-level assemblies in other crustaceans (Table 3). Our assembly has greater contiguity than all other chromosome-level crustacean assemblies, except for the relatively giant genome of the blue king crab *Paralithodes platypus*. Moreover, our barnacle assembly has relatively high BUSCO scores. Notably, this assembly has higher contiguity and BUSCO scores compared to all other barnacle genome assemblies (Table 3).

### Phylogenomics

The Pollicipedomorpha has proven to be one of the most difficult clades of stalked and acorn barnacles to resolve in phylogenetic analyses, in terms of both its relationship to the other barnacle orders and also the relationships among its 4 genera [7]. Barnacle phylogenies based on morphology or molecular data have yielded very different results depending on the characters or genes used (e.g., Fig. 2A). Historically, larval characters have played a major role in our understanding of the phylogeny and evolution of thecostacans, which is especially true for the parasitic taxa that often lack traditional adult barnacle characters [5, 7]. Still, using a matrix of 41 larval characters across all major barnacle lineages, Pérez-Losada et al. [129] were not able to resolve thecostracan relationships below the sub-class level. Similar attempts to code larval characters for phylogenetic inference (e.g., [133, 134]) ultimately failed at recovering topologies consistent with those inferred using nuclear and mitochondrial protein coding and ribosomal genes (Fig 2A). While adult characters consistently unite *Capitulum* and *Pollicipes*, larval characters have separated the genera in some analyses. Further obscuring this situation, phylogenetic analyses of characters from different larval stages have led to conflicting phylogenies. For example, because *Pollicipes* and balanomorphans share some naupliar characters (e.g., more oval carapaces and lack of marginal carapace spines; Fig. 1A and B; [132–134]) that are absent in *Capitulum*, Korn [135] and Newman and Ross [136] found *Pollicipes* nested within Balanomorpha and Korn [135] found *Capitulum* within Scallpelomorpha. Cypris larval characters, however, united *Capitulum* and *Pollicipes* (i.e., heavily ornamented carapaces and third antennal segments surrounded by a series of velar flaps or filaments [137]).

Here, we resolved part of the Pollicipedomorpha conundrum with phylogenomic analyses of nearly 2 million AA positions from 14 barnacles. We found robust support for the independence of the order and its sister relationship with the Balanomorpha. Reinterpreting the larval characters in light of this phylogeny suggests that the shared naupliar features in *Pollicipes* and balamorphan taxa that are lacking in *Capitulum* are the result of homoplasy. Still, questions remain regarding the interrelationships of the 4 pollicipedomorphan genera. To further resolve the situation, *Analesma* and *Lithotrya* must be included in future phylogenomic analyses. Sampling the 8 remaining species in the Pollicipedomorpha is thus within reach and is crucial to understanding the evolution of key larval characters in this morphologically diverse order. Taken together, this work supports the validity of the Pollicipedomorpha and highlights the fact that larval character analyses should be coupled with robust molecular phylogenetic hypotheses to understand barnacle evolution.

### Differential gene expression of nauplii and adults

The differences in the transcriptomes of the nauplius and adult *P. pollicipes* are striking. Nearly half of all genes (i.e., 11,846/24,664) undergo significant differential expression between these stages. These transcriptional differences reflect the vastly different biology of larval and adult barnacles. For example, among the 100 most differentially expressed genes, cuticle proteins were highly upregulated in the nauplius, a stage in which cuticle is rapidly being modified as individuals molt 6 times within 10–25 days [138] (Fig. 1A–C). Similarly, genes related to vision were upregulated in nauplii, which is unsurprising considering that nauplii possess an eye whereas adults do not. Numerous structural proteins (e.g., chitin, keratin, collagen) were upregulated in adults; at this stage, barnacles grow orders of magnitude larger and build shell plates and a peduncle >2× their body length (Fig. 3D). One of the most upregulated genes in adults was vitellogenin-1-like (Fig. 3D), which is in alignment with the developmental biology of *Pollicipes*; adults provide large yolk stores that the non-feeding (lecithotrophic) first nauplius stage relies on. Adults' DEGs were also enriched for heme proteins (Fig. 3C), which may be necessary for oxygen delivery given that, at their size, adult gooseneck barnacles cannot rely on passive diffusion of oxygen like nauplii.

GO enrichment analysis and KEGG pathways also revealed broader patterns. Nauplii upregulated genes involved in tissue morphogenesis, such as homophilic cell adhesion genes including cadherins (Fig. 3B, Supplementary Table S5) [139], while adults upregulated proteins that may be involved in tissue modeling and adhesion, such as cuticle-binding proteins, chitin-binding proteins, and collagen trimers (Fig. 3C, Supplementary Table S6). At the broadest levels, the enrichment analysis showed that nauplii upregulate excretory (i.e., secretory) proteins, while adult barnacles upregulated more membrane-bound proteins (Fig. 3B and C, Supplementary Tables S5 and S6). Furthermore, preliminary pathway analysis showed that adults upregulate genes involved in carbohydrate metabolism, lipid metabolism, metabolism of cofactors and vitamins, and amino acid metabolism. One likely factor at play here is dietary change; nauplii feed on small single-celled phytoplankton, while adults feed mostly on small crustaceans [138, 140]; concordantly, DEGs were often involved in macronutrient metabolism in the KEGG pathway and GO enrichment analyses. A total of 332 lncRNAs were also differentially expressed between adults and nauplii (Fig. 3D). Given that lncRNAs are thought to play important roles in gene regulation [141], further research is needed to assess their potential functions.

Overall, the differences in larval and adult transcriptomes of *P. pollicipes* are substantial. This is true even when compared to transcriptional differences typically seen in other arthropods with profound metamorphoses, such as holometabolous insects. For example, in *Anopheles* [142], *Apis* [143], *Drosophila* [144], and other insects [145–147], typically ∼3–30% of their genes are differentially expressed between larva and adult stages compared to the 48% of genes observed here in *P. pollicipes*. Stark differences in the biology of the nauplius and adult barnacle stages appear to be reflected in drastic transcriptomic differences both in the degree of expression of nearly half of their genes, as well as the number of genes (>4,000) that exhibited stage-specific expression (Fig. 3A). A noteworthy limitation of this DEG analysis is that there were only 2 replicates each for the nauplius and adult stages, although the nauplius libraries were prepared from pooled individuals [127], which reduces sample variability and helps compensate for the lack of replication [148]. Still, a better understanding of transcriptional differences across *P. pollicipes* life stages requires additional replication, ideally with nauplius samples separated by stage (i.e., N1–N6), and samples of the cyprid stage (Fig. 1).

## Conclusions

By combining Illumina short reads, PacBio long reads, and Hi-C and CHi-C chromatin-conformation capture data, we produced a high-quality genome assembly and annotation for the gooseneck barnacle *P. pollicipes*. This is one of the most contiguous crustacean genomes to date and, to our knowledge, the most complete assembly for a barnacle species. Using the genome annotation and transcriptomic data from 13 other barnacles, we completed phylogenetic analyses with the greatest number of orthologs and AA positions to date for barnacles and showed that the Pollicipedomorpha is a monophyletic order sister to Balanomorpha (Fig. 2). Our DEG analysis of nauplii and adult transcriptomes revealed large differences in metabolic function and regulation in *P. pollicipes*, underlying the vast difference in lifestyle between these 2 stages. This study hence provides a valuable example of good genomic practices, high-quality genomic resources for a key group of crustaceans, and valuable insights into the evolution and development of barnacles.

## Supplementary Material

giac021_Supplemental_File

giac021_GIGA-D-21-00365_Original_Submission

giac021_GIGA-D-21-00365_Revision_1

giac021_GIGA-D-21-00365_Revision_2

giac021_Response_to_Reviewer_Comments_Revision_1

giac021_Response_to_Reviewer_Comments_Revision_2

giac021_Reviewer_1_Report_Original_Submission Chao Bian -- 11/23/2021 Reviewed

giac021_Reviewer_1_Report_Revision_1 Chao Bian -- 1/18/2022 Reviewed

giac021_Reviewer_1_Report_Revision_2 Chao Bian -- 1/20/2022 Reviewed

giac021_Reviewer_2_Report_Original_Submission Rafael Zardoya, PhD -- 12/7/2021 Reviewed

## Data Availability

BioProject: PRJNA614970 (*Pollicipes pollicipes*) Associated BioProjects: PRJNA533106 (EBP); PRJNA649812 (GIGA) Mitochondrial genome accession: CM029732.1 Biosample: SAMN14444043 WGS Project: JAAVLY02 GenBank (and RefSeq) assembly accession: GCA_011947565.2 NCBI Annotation (Release 100): https://www.ncbi.nlm.nih.gov/genome/annotation_euk/Pollicipes_pollicipes/100/ SRA accessions: SRR11456527-40 (PacBio), SRR12730898 (Illumina NextSeq WGS), SRR11483033-34 (HiC), SRR11483035-36 (CHi-C) Hologenophore: USNM 1622609 Paragenophores: USNM 1622610 (lot of 6 specimens) All supporting data and materials are available in the *GigaScience* GigaDB database [149].

## Additional Files

**Supplementary Figure S1**. Distribution map of *Pollicipes pollicipes* based on the 159 occurrences with location data available (of 372 total occurrences) from GBIF GBIF.org (28 March 2020) GBIF Occurrence Download https://doi.org/10.15468/dl.qjbfsz. Inlaid in the distribution map is a magnified view of the boxed location near Vigo, Spain where the specimens were collected for this study.

**Supplementary Figure S2**. Genome assembly pipeline.

**Supplementary Figure S3**. Distinct k-mer frequency distribution for the Illumina WGS reads and two normal distributions mixture for bimodal model (red).

**Supplementary Figure S4**. Histogram of read lengths for Pacbio reads (Nanoplot output).

**Supplementary Figure S5**. HiC contact heat map for 17 scaffolds more than 1 Mbp in length. Each point color represents the number of HiC read-pairs in this bin. White vertical and horizontal lines have been added to show the borders between scaffolds.

**Supplementary Figure S6**. Barplot of the lengths of the 20 largest scaffolds.

**Supplementary Figure S7**. Read-depth histogram for Illumina short reads. X-axis - average read depth, Y-axis - number of reads with this depth. Red arrows represent coverage cutoffs for heterozygous and homozygous peaks.

**Supplementary Figure S8**. Pairwise alignments (a) assembled mitochondrial contig and *P. polymerus* mitochondrial DNA (b) assembled contig aligned to itself.

**Supplementary Figure S9**. Nauplius Molecular Function GO enrichment. TopGO subgraph induced by Molecular Function terms with q < 0.0001. Boxes indicate significant terms. Box color represents the relative significance, ranging from yellow (less significant) to dark red (most significant).

**Supplementary Figure S10**. Nauplius Biological Process GO enrichment. TopGO subgraph induced by Biological Process terms with q < 0.001. Boxes indicate significant terms. Box color represents the relative significance, ranging from yellow (less significant) to dark red (most significant).

**Supplementary Figure S11**. Nauplius Cellular Component GO enrichment. TopGO subgraph induced by Cellular Component terms with q < 0.05. Box color represents the relative significance, ranging from yellow (less significant) to dark red (most significant).

**Supplementary Figure S** **12**. Adult Molecular Function GO enrichment. TopGO subgraph induced by Molecular Function terms with q < 0.0001. Box color represents the relative significance, ranging from yellow (less significant) to dark red (most significant).

**Supplementary Figure S13**. Adult Biological Process GO enrichment. TopGO subgraph induced by Biological Process terms with q < 0.001. Box color represents the relative significance, ranging from yellow (less significant) to dark red (most significant).

**Supplementary Figure S** **14**. Adult Cellular Component GO enrichment. TopGO subgraph induced by Cellular Component terms with q < 0.05. Box color represents the relative significance, ranging from yellow (less significant) to dark red (most significant).

**Supplementary Table S** **1**. QUAST summary report.

**Supplementary Table S** **2**. BUSCO summary report.

**Supplementary Table S** **3**. Pilon polishing summary.

**Supplementary Table S4**. *Pollicipes pollicipes* RNA-Seq data on NCBI used for genome annotation.

**Supplementary Table S5**. Nauplius GO enrichment.

**Supplementary Table S6**. Adult GO enrichment.

**Supplementary Table S7**. Nauplius DEG KEGG Orthologs.

**Supplementary Table S8**. Adult DEG KEGG Orthologs.

## Abbreviations

AA: amino acid; BLAST: Basic Local Alignment Search Tool; bp: base pairs; BS: bootstrap; BUSCO: Benchmarking Universal Single-Copy Orthologs; DEG: differentially expressed gene; EBP: Earth Biogenome Project; FAIR: findable, accessible, interoperable, and reusable; FDR: false discovery rate; FPKM: fragments per kilobase of transcript per million mapped reads; GO: Gene Ontology; GIGA: Global Invertebrate Genomics Alliance; kb: kilobase pairs; KEGG: Kyoto Encyclopedia of Genes and Genomes; lncRNA: long non-coding RNA; MAFFT: Multiple Alignment using Fast Fourier Transform; Mb: megabase pairs; ML: maximum likelihood; N1–N6: nauplius 1–nauplius 6; NCBI: National Center for Biotechnology Information; PacBio: Pacific Biosciences; PP: posterior probability; RAxML: Randomized Axelerated Maximum Likelihood; SEM: scanning electron microscopy; SMRT: single-molecule real-time; SRA: Sequence Read Archive; USNM: US National Museum of Natural History; WGS: whole-genome sequencing.
