# Supplementary material for: Chromosome-level genome assembly, annotation, and phylogenomics of the gooseneck barnacle Pollicipes pollicipes
Source: Gigascience. 2022 Mar 12;11:giac021. doi: 10.1093/gigascience/giac021 (PMC8917513; doi:10.1093/gigascience/giac021)
Supplement: giac021_Supplemental_File [file giac021_supplemental_file.docx]

**Supplemental Information**

**Table S1**. QUAST summary report.


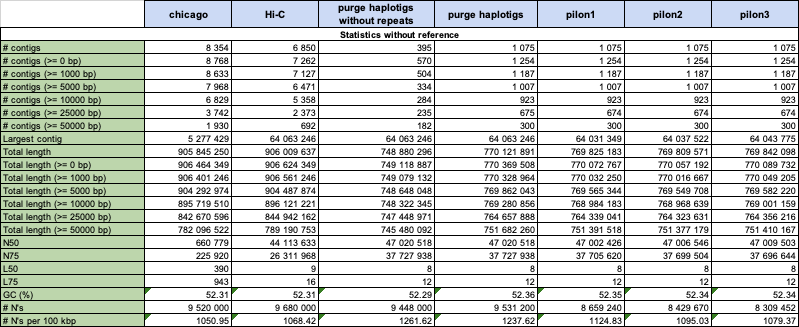


**Table S2**. BUSCO summary report.

|  | BUSCO Arthropoda | | | | | | BUSCO Metazoa | | | | | | BUSCO Eukaryota | | | | | |
| --- | --- | --- | --- | --- | --- | --- | --- | --- | --- | --- | --- | --- | --- | --- | --- | --- | --- | --- |
| assemblies | complete | single | duplicated | fragmented | missing | number | complete | single | duplicated | fragmented | missing | number | complete | single | duplicated | fragmented | missing | number |
| FALCON_ARROW | 89.3 | 51.7 | 37.6 | 4.2 | 6.5 | 1013 | 89.7 | 53.6 | 36.1 | 5.6 | 4.7 | 954 | 90.2 | 55.7 | 34.5 | 7.1 | 2.7 | 255 |
| CHi-C | 90.2 | 62.5 | 27.7 | 3.7 | 6.1 |  | 90.3 | 62.2 | 28.1 | 5.2 | 4.5 |  | 91 | 65.9 | 25.1 | 6.7 | 2.3 |  |
| Hi-C | 90.4 | 63.7 | 26.7 | 3.6 | 6 |  | 90.4 | 62.9 | 27.5 | 5.1 | 4.5 |  | 91 | 67.5 | 23.5 | 6.7 | 2.3 |  |
| PurgeHaplo_without_repeats | 90.2 | 72 | 18.2 | 3.6 | 6.2 |  | 90 | 70.9 | 19.1 | 5.2 | 4.8 |  | 91 | 74.9 | 16.1 | 6.7 | 2.3 |  |
| PurgeHaplo | 90.1 | 71 | 19.1 | 3.5 | 6.4 |  | 89.9 | 69.4 | 20.5 | 5.1 | 5 |  | 91 | 74.1 | 16.9 | 6.7 | 2.3 |  |
| Pilon 1 | 90.4 | 70.3 | 20.1 | 3.5 | 6.1 |  | 91.1 | 68.8 | 22.3 | 4.5 | 4.4 |  | 91.7 | 73.3 | 18.4 | 5.9 | 2.4 |  |
| Pilon 2 | 90.4 | 69.8 | 20.6 | 3.4 | 6.2 |  | 91.3 | 68.3 | 23 | 4.3 | 4.4 |  | 91.7 | 72.5 | 19.2 | 5.9 | 2.4 |  |
| Pilon 3 (final) | 90.5 | 69.4 | 21.1 | 3.3 | 6.2 |  | 91.2 | 67.8 | 23.4 | 4.4 | 4.4 |  | 91.8 | 72.2 | 19.6 | 5.9 | 2.3 |  |

**Table S3**. Pilon polishing statistical summary.

|  | round 1 | round 2 | round 3 |
| --- | --- | --- | --- |
| total bases: | 770 369 508 | 770 072 767 | 770 057 192 |
| confirmed: | 646 159 414 (83.88%) | 653 898 879 (84.91%) | 657 234 049 (85.35%) |
|  |  |  |  |
| snps: | 681 850 (0.089%) | 227 062 (0.029%) | 103 564 (0.013%) |
| insertions: | 208 860 | 36 477 | 13 327 |
| insert bases: | 277 671 (0.036%) | 61 124 (0.0079%) | 25 577 (0.0033%) |
| deletions: | 372 644 | 51 395 | 17 313 |
| delete bases: | 591 383 (0.077%) | 92 332 (0.012%) | 35 841 (0.0047%) |
|  |  |  |  |
| Gaps filled: | 3 202 | 1 357 | 800 |
| Gaps closed: | 70 | 17 | 1 |
| Partially filled: | 3 132 | 1 340 | 799 |
| sequences with gaps filled: | 115 | 57 | 42 |
| deleted: | 333 946bp (0.043%) | 64 009bp (0.0083%) | 26 555bp (0.0034%) |
| added: | 1 596 612bp (0.21%) | 44 1231bp (0.057%) | 250 674bp (0.033%) |
|  |  |  |  |
| Breaks fixed: | 56 724 | 28 054 | 21 841 |
| sequences with break fix: | 574 | 392 | 283 |
| deleted: | 14 531 906bp (1.9%) | 694 1976bp (0.9%) | 5 143 895bp (0.67%) |
| added: | 14 123 253bp (1.8%) | 680 6246bp (0.88%) | 5 082 718bp (0.66%) |

**Table S4**. Transcriptome data (HiSeq RNA-Seq data) for *Pollicipes pollicipes* from NCBI used for genome annotation.

| **Experiment Accession** | **Tissue** | **Study Accession** | **Sample Accession** | **Total Size, Mb** | **Total Spots** | **Total Bases** |
| --- | --- | --- | --- | --- | --- | --- |
| SRX3775828 | Cement gland | SRP134179 | SRS3029963 | 4697.77 | 43,736,131 | 11734345263 |
| SRX3009428 | adult1 | SRP112554 | SRS2359512 | 6329.36 | 44,871,270 | 9063996540 |
| SRX3009427 | adult2 | SRP112554 | SRS2359513 | 4793.31 | 34,339,943 | 6936668486 |
| SRX3009426 | larvae1 | SRP112554 | SRS2359511 | 3675.73 | 26,054,881 | 5263085962 |
| SRX3009425 | larvae2 | SRP112554 | SRS2359510 | 2272.08 | 16,266,423 | 3285817446 |

| **Table S5. Nauplius GO Enrichment** | |  |  |  |  |  |
| --- | --- | --- | --- | --- | --- | --- |
| GO.ID | Term | Annotated | Significant | Expected | classicFisher | fdr |
| *Molecular Function* |  |  |  |  |  |  |
| GO:0004175 | endopeptidase activity | 422 | 103 | 35.63 | 2.00E-24 | 2.00E-22 |
| GO:0008233 | peptidase activity | 615 | 122 | 51.93 | 1.90E-20 | 9.50E-19 |
| GO:0004252 | serine-type endopeptidase activity | 296 | 77 | 24.99 | 3.90E-20 | 1.30E-18 |
| GO:0008236 | serine-type peptidase activity | 319 | 78 | 26.94 | 1.20E-18 | 2.40E-17 |
| GO:0017171 | serine hydrolase activity | 319 | 78 | 26.94 | 1.20E-18 | 2.40E-17 |
| GO:0016787 | hydrolase activity | 1583 | 214 | 133.67 | 2.40E-14 | 4.00E-13 |
| GO:0140096 | catalytic activity, acting on a protein | 1226 | 171 | 103.52 | 1.80E-12 | 2.50E-11 |
| GO:0005509 | calcium ion binding | 258 | 58 | 21.79 | 2.00E-12 | 2.50E-11 |
| GO:0008569 | minus-end-directed microtubule motor activity | 18 | 13 | 1.52 | 5.80E-11 | 5.80E-10 |
| GO:1990939 | ATP-dependent microtubule motor activity | 18 | 13 | 1.52 | 5.80E-11 | 5.80E-10 |
| GO:0008237 | metallopeptidase activity | 165 | 35 | 13.93 | 2.40E-07 | 2.18E-06 |
| GO:0008061 | chitin binding | 126 | 28 | 10.64 | 1.50E-06 | 1.25E-05 |
| GO:0004222 | metalloendopeptidase activity | 101 | 24 | 8.53 | 2.40E-06 | 1.85E-05 |
| GO:0003777 | microtubule motor activity | 65 | 18 | 5.49 | 4.30E-06 | 3.07E-05 |
| GO:0030414 | peptidase inhibitor activity | 127 | 26 | 10.72 | 1.70E-05 | 0.00010625 |
| GO:0061134 | peptidase regulator activity | 127 | 26 | 10.72 | 1.70E-05 | 0.00010625 |
| GO:0003824 | catalytic activity | 3566 | 352 | 301.11 | 4.60E-05 | 0.000270588 |
| GO:0004857 | enzyme inhibitor activity | 144 | 27 | 12.16 | 6.00E-05 | 0.000333333 |
| GO:0003779 | actin binding | 52 | 14 | 4.39 | 7.00E-05 | 0.000365 |
| GO:0003774 | motor activity | 100 | 21 | 8.44 | 7.30E-05 | 0.000365 |
| GO:0004180 | carboxypeptidase activity | 19 | 8 | 1.6 | 8.10E-05 | 0.000385714 |
| GO:0004181 | metallocarboxypeptidase activity | 15 | 7 | 1.27 | 0.0001 | 0.000454545 |
| GO:0016758 | transferase activity, transferring hexos... | 100 | 20 | 8.44 | 0.00022 | 0.000956522 |
| GO:0005319 | lipid transporter activity | 29 | 9 | 2.45 | 0.00044 | 0.001833333 |
| GO:0008238 | exopeptidase activity | 48 | 12 | 4.05 | 0.00049 | 0.00196 |
| GO:0004656 | procollagen-proline 4-dioxygenase activi... | 6 | 4 | 0.51 | 0.00066 | 0.002275862 |
| GO:0019798 | procollagen-proline dioxygenase activity | 6 | 4 | 0.51 | 0.00066 | 0.002275862 |
| GO:0031543 | peptidyl-proline dioxygenase activity | 6 | 4 | 0.51 | 0.00066 | 0.002275862 |
| GO:0031545 | peptidyl-proline 4-dioxygenase activity | 6 | 4 | 0.51 | 0.00066 | 0.002275862 |
| GO:0051015 | actin filament binding | 10 | 5 | 0.84 | 0.00074 | 0.002466667 |
| GO:0016706 | 2-oxoglutarate-dependent dioxygenase act... | 7 | 4 | 0.59 | 0.00143 | 0.004612903 |
| GO:0004725 | protein tyrosine phosphatase activity | 59 | 12 | 4.98 | 0.00331 | 0.01034375 |
| GO:0030234 | enzyme regulator activity | 196 | 28 | 16.55 | 0.00395 | 0.011969697 |
| GO:0000030 | mannosyltransferase activity | 14 | 5 | 1.18 | 0.00445 | 0.013088235 |
| GO:0015018 | galactosylgalactosylxylosylprotein 3-bet... | 6 | 3 | 0.51 | 0.00988 | 0.027444444 |
| GO:0015020 | glucuronosyltransferase activity | 6 | 3 | 0.51 | 0.00988 | 0.027444444 |
| GO:0016715 | oxidoreductase activity, acting on paire... | 11 | 4 | 0.93 | 0.01028 | 0.027783784 |
| GO:0005507 | copper ion binding | 17 | 5 | 1.44 | 0.01111 | 0.029236842 |
| GO:0016757 | transferase activity, transferring glyco... | 156 | 22 | 13.17 | 0.0115 | 0.029487179 |
| GO:0008235 | metalloexopeptidase activity | 31 | 7 | 2.62 | 0.013 | 0.0325 |
| GO:0046872 | metal ion binding | 812 | 86 | 68.57 | 0.01428 | 0.032727273 |
| GO:0008241 | peptidyl-dipeptidase activity | 12 | 4 | 1.01 | 0.0144 | 0.032727273 |
| GO:0017128 | phospholipid scramblase activity | 12 | 4 | 1.01 | 0.0144 | 0.032727273 |
| GO:0140303 | intramembrane lipid transporter activity | 12 | 4 | 1.01 | 0.0144 | 0.032727273 |
| GO:0008146 | sulfotransferase activity | 89 | 14 | 7.52 | 0.01652 | 0.035604167 |
| GO:0004721 | phosphoprotein phosphatase activity | 98 | 15 | 8.28 | 0.01694 | 0.035604167 |
| GO:0043169 | cation binding | 818 | 86 | 69.07 | 0.01702 | 0.035604167 |
| GO:0008092 | cytoskeletal protein binding | 134 | 19 | 11.31 | 0.01709 | 0.035604167 |
| GO:0030674 | protein-macromolecule adaptor activity | 13 | 4 | 1.1 | 0.01944 | 0.039673469 |
| GO:0008194 | UDP-glycosyltransferase activity | 50 | 9 | 4.22 | 0.02252 | 0.04504 |
| GO:0004806 | triglyceride lipase activity | 8 | 3 | 0.68 | 0.02432 | 0.047686275 |
| GO:0005548 | phospholipid transporter activity | 14 | 4 | 1.18 | 0.02544 | 0.048923077 |
| *Biological Process* |  |  |  |  |  |  |
| GO:0006508 | proteolysis | 638 | 125 | 58.26 | 8.80E-19 | 8.80E-17 |
| GO:0007156 | homophilic cell adhesion via plasma memb... | 49 | 20 | 4.47 | 2.50E-09 | 6.25E-08 |
| GO:0098609 | cell-cell adhesion | 49 | 20 | 4.47 | 2.50E-09 | 6.25E-08 |
| GO:0098742 | cell-cell adhesion via plasma-membrane a... | 49 | 20 | 4.47 | 2.50E-09 | 6.25E-08 |
| GO:0007155 | cell adhesion | 72 | 24 | 6.57 | 7.90E-09 | 1.32E-07 |
| GO:0022610 | biological adhesion | 72 | 24 | 6.57 | 7.90E-09 | 1.32E-07 |
| GO:0019538 | protein metabolic process | 1506 | 189 | 137.52 | 6.60E-08 | 9.43E-07 |
| GO:1901564 | organonitrogen compound metabolic proces... | 1680 | 202 | 153.41 | 5.80E-07 | 7.25E-06 |
| GO:0007018 | microtubule-based movement | 71 | 19 | 6.48 | 1.20E-05 | 0.000133333 |
| GO:0006928 | movement of cell or subcellular componen... | 72 | 19 | 6.57 | 1.50E-05 | 0.00015 |
| GO:0007017 | microtubule-based process | 91 | 20 | 8.31 | 0.00015 | 0.001133333 |
| GO:0006486 | protein glycosylation | 78 | 18 | 7.12 | 0.00017 | 0.001133333 |
| GO:0009101 | glycoprotein biosynthetic process | 78 | 18 | 7.12 | 0.00017 | 0.001133333 |
| GO:0043413 | macromolecule glycosylation | 78 | 18 | 7.12 | 0.00017 | 0.001133333 |
| GO:0070085 | glycosylation | 78 | 18 | 7.12 | 0.00017 | 0.001133333 |
| GO:0009100 | glycoprotein metabolic process | 79 | 18 | 7.21 | 0.0002 | 0.00125 |
| GO:0006493 | protein O-linked glycosylation | 8 | 5 | 0.73 | 0.00028 | 0.001647059 |
| GO:0009166 | nucleotide catabolic process | 13 | 6 | 1.19 | 0.00055 | 0.003055556 |
| GO:0046434 | organophosphate catabolic process | 23 | 8 | 2.1 | 0.00065 | 0.003421053 |
| GO:0006869 | lipid transport | 36 | 10 | 3.29 | 0.00105 | 0.004818182 |
| GO:0006720 | isoprenoid metabolic process | 10 | 5 | 0.91 | 0.00106 | 0.004818182 |
| GO:0008299 | isoprenoid biosynthetic process | 10 | 5 | 0.91 | 0.00106 | 0.004818182 |
| GO:1901135 | carbohydrate derivative metabolic proces... | 182 | 29 | 16.62 | 0.00191 | 0.008304348 |
| GO:1901292 | nucleoside phosphate catabolic process | 16 | 6 | 1.46 | 0.00203 | 0.008458333 |
| GO:0010876 | lipid localization | 40 | 10 | 3.65 | 0.00251 | 0.01004 |
| GO:0034404 | nucleobase-containing small molecule bio... | 17 | 6 | 1.55 | 0.0029 | 0.011153846 |
| GO:1901137 | carbohydrate derivative biosynthetic pro... | 147 | 23 | 13.42 | 0.00682 | 0.025259259 |
| GO:0034655 | nucleobase-containing compound catabolic... | 33 | 8 | 3.01 | 0.00814 | 0.029071429 |
| GO:0015711 | organic anion transport | 28 | 7 | 2.56 | 0.01099 | 0.037896552 |
| GO:0006820 | anion transport | 57 | 11 | 5.2 | 0.01262 | 0.042066667 |
| *Cellular Component* |  |  |  |  |  |  |
| GO:0005576 | extracellular region | 246 | 53 | 22.7 | 5.50E-10 | 5.50E-08 |
| GO:0030286 | dynein complex | 26 | 13 | 2.4 | 9.10E-08 | 4.55E-06 |
| GO:0015630 | microtubule cytoskeleton | 36 | 15 | 3.32 | 1.90E-07 | 6.00E-06 |
| GO:0005875 | microtubule associated complex | 32 | 14 | 2.95 | 2.40E-07 | 6.00E-06 |
| GO:0005856 | cytoskeleton | 93 | 21 | 8.58 | 6.80E-05 | 0.00136 |
| GO:0005783 | endoplasmic reticulum | 25 | 8 | 2.31 | 0.0013 | 0.021666667 |

| **Table S6. Adult GO Enrichment** | |  |  |  |  |  |
| --- | --- | --- | --- | --- | --- | --- |
| GO.ID | Term | Annotated | Significant | Expected | classicFisher | fdr |
| *Molecular Function* |  |  |  |  |  |  |
| GO:0042302 | structural constituent of cuticle | 417 | 162 | 58.83 | < 1e-30 | <1.00E-23 |
| GO:0005506 | iron ion binding | 148 | 74 | 20.88 | 1.10E-25 | 1.10E-23 |
| GO:0005198 | structural molecule activity | 583 | 172 | 82.25 | 1.30E-23 | 6.50E-22 |
| GO:0020037 | heme binding | 151 | 67 | 21.3 | 9.70E-20 | 3.23E-18 |
| GO:0046906 | tetrapyrrole binding | 158 | 67 | 22.29 | 1.90E-18 | 4.75E-17 |
| GO:0016705 | oxidoreductase activity, acting on paire... | 154 | 65 | 21.73 | 8.20E-18 | 1.64E-16 |
| GO:0008061 | chitin binding | 126 | 56 | 17.78 | 9.70E-17 | 1.62E-15 |
| GO:0022857 | transmembrane transporter activity | 820 | 196 | 115.68 | 1.90E-15 | 2.71E-14 |
| GO:0005215 | transporter activity | 858 | 202 | 121.04 | 3.50E-15 | 4.38E-14 |
| GO:0016491 | oxidoreductase activity | 516 | 137 | 72.79 | 1.20E-14 | 1.33E-13 |
| GO:0016810 | hydrolase activity, acting on carbon-nit... | 29 | 17 | 4.09 | 3.00E-08 | 3.00E-07 |
| GO:0046914 | transition metal ion binding | 422 | 100 | 59.53 | 4.50E-08 | 4.09E-07 |
| GO:0015081 | sodium ion transmembrane transporter act... | 55 | 22 | 7.76 | 2.00E-06 | 1.67E-05 |
| GO:0015267 | channel activity | 312 | 74 | 44.01 | 2.60E-06 | 1.86E-05 |
| GO:0022803 | passive transmembrane transporter activi... | 312 | 74 | 44.01 | 2.60E-06 | 1.86E-05 |
| GO:0005272 | sodium channel activity | 52 | 21 | 7.34 | 2.80E-06 | 1.87E-05 |
| GO:0046873 | metal ion transmembrane transporter acti... | 91 | 30 | 12.84 | 3.70E-06 | 2.31E-05 |
| GO:0015318 | inorganic molecular entity transmembrane... | 401 | 89 | 56.57 | 4.90E-06 | 2.88E-05 |
| GO:0016811 | hydrolase activity, acting on carbon-nit... | 18 | 11 | 2.54 | 5.20E-06 | 2.89E-05 |
| GO:0005216 | ion channel activity | 307 | 71 | 43.31 | 1.10E-05 | 5.79E-05 |
| GO:0015075 | ion transmembrane transporter activity | 422 | 90 | 59.53 | 2.30E-05 | 0.000115 |
| GO:0005201 | extracellular matrix structural constitu... | 13 | 8 | 1.83 | 0.0001 | 0.00047619 |
| GO:0043169 | cation binding | 818 | 152 | 115.4 | 0.00011 | 5.00E-04 |
| GO:0046872 | metal ion binding | 812 | 151 | 114.55 | 0.00012 | 0.000521739 |
| GO:0005261 | cation channel activity | 67 | 21 | 9.45 | 0.00023 | 0.000958333 |
| GO:0004061 | arylformamidase activity | 6 | 5 | 0.85 | 0.00029 | 0.00116 |
| GO:0004252 | serine-type endopeptidase activity | 296 | 62 | 41.76 | 0.0007 | 0.002571429 |
| GO:0030170 | pyridoxal phosphate binding | 58 | 18 | 8.18 | 0.00072 | 0.002571429 |
| GO:0070279 | vitamin B6 binding | 58 | 18 | 8.18 | 0.00072 | 0.002571429 |
| GO:0008171 | O-methyltransferase activity | 7 | 5 | 0.99 | 0.00091 | 0.003137931 |
| GO:0008146 | sulfotransferase activity | 89 | 24 | 12.56 | 0.00102 | 0.0034 |
| GO:0004888 | transmembrane signaling receptor activit... | 137 | 33 | 19.33 | 0.00115 | 0.00369697 |
| GO:0004930 | G protein-coupled receptor activity | 85 | 23 | 11.99 | 0.00122 | 0.00369697 |
| GO:0004175 | endopeptidase activity | 422 | 82 | 59.53 | 0.00122 | 0.00369697 |
| GO:0038023 | signaling receptor activity | 139 | 33 | 19.61 | 0.0015 | 0.004285714 |
| GO:0060089 | molecular transducer activity | 139 | 33 | 19.61 | 0.0015 | 0.004285714 |
| GO:0022890 | inorganic cation transmembrane transport... | 151 | 35 | 21.3 | 0.00171 | 0.00475 |
| GO:0008236 | serine-type peptidase activity | 319 | 64 | 45 | 0.00183 | 0.004815789 |
| GO:0017171 | serine hydrolase activity | 319 | 64 | 45 | 0.00183 | 0.004815789 |
| GO:0005544 | calcium-dependent phospholipid binding | 27 | 10 | 3.81 | 0.00258 | 0.006615385 |
| GO:0016782 | transferase activity, transferring sulfu... | 95 | 24 | 13.4 | 0.00266 | 0.00665 |
| GO:0019239 | deaminase activity | 12 | 6 | 1.69 | 0.00336 | 0.008195122 |
| GO:0015077 | monovalent inorganic cation transmembran... | 114 | 27 | 16.08 | 0.004 | 0.00952381 |
| GO:0008324 | cation transmembrane transporter activit... | 165 | 36 | 23.28 | 0.00438 | 0.010186047 |
| GO:0015276 | ligand-gated ion channel activity | 145 | 32 | 20.46 | 0.00583 | 0.012955556 |
| GO:0022834 | ligand-gated channel activity | 145 | 32 | 20.46 | 0.00583 | 0.012955556 |
| GO:0008194 | UDP-glycosyltransferase activity | 50 | 14 | 7.05 | 0.00762 | 0.016565217 |
| GO:0022836 | gated channel activity | 160 | 34 | 22.57 | 0.00839 | 0.017851064 |
| GO:0004867 | serine-type endopeptidase inhibitor acti... | 36 | 11 | 5.08 | 0.00863 | 0.017979167 |
| GO:0004359 | glutaminase activity | 7 | 4 | 0.99 | 0.00967 | 0.019734694 |
| GO:0016701 | oxidoreductase activity, acting on singl... | 28 | 9 | 3.95 | 0.01194 | 0.02388 |
| GO:0019842 | vitamin binding | 74 | 18 | 10.44 | 0.01286 | 0.025215686 |
| GO:0008233 | peptidase activity | 615 | 106 | 86.76 | 0.01371 | 0.026365385 |
| GO:0004623 | phospholipase A2 activity | 8 | 4 | 1.13 | 0.0172 | 0.031851852 |
| GO:0015293 | symporter activity | 8 | 4 | 1.13 | 0.0172 | 0.031851852 |
| GO:0017128 | phospholipid scramblase activity | 12 | 5 | 1.69 | 0.01857 | 0.033160714 |
| GO:0140303 | intramembrane lipid transporter activity | 12 | 5 | 1.69 | 0.01857 | 0.033160714 |
| GO:0004356 | glutamate-ammonia ligase activity | 5 | 3 | 0.71 | 0.02243 | 0.038016949 |
| GO:0016211 | ammonia ligase activity | 5 | 3 | 0.71 | 0.02243 | 0.038016949 |
| GO:0016880 | acid-ammonia (or amide) ligase activity | 5 | 3 | 0.71 | 0.02243 | 0.038016949 |
| GO:0008234 | cysteine-type peptidase activity | 62 | 15 | 8.75 | 0.02304 | 0.0384 |
| GO:0016747 | transferase activity, transferring acyl ... | 108 | 23 | 15.24 | 0.02619 | 0.042934426 |
| *Biological Process* |  |  |  |  |  |  |
| GO:0055114 | oxidation-reduction process | 550 | 152 | 75.07 | 2.20E-20 | 2.20E-18 |
| GO:0055085 | transmembrane transport | 637 | 137 | 86.94 | 3.70E-09 | 1.85E-07 |
| GO:0006810 | transport | 1053 | 201 | 143.72 | 1.70E-08 | 5.50E-07 |
| GO:0051234 | establishment of localization | 1056 | 201 | 144.13 | 2.20E-08 | 5.50E-07 |
| GO:0051179 | localization | 1068 | 201 | 145.77 | 5.70E-08 | 1.14E-06 |
| GO:0006814 | sodium ion transport | 53 | 21 | 7.23 | 2.20E-06 | 3.67E-05 |
| GO:0006820 | anion transport | 57 | 21 | 7.78 | 8.70E-06 | 0.000124286 |
| GO:1901605 | alpha-amino acid metabolic process | 33 | 14 | 4.5 | 4.50E-05 | 0.0005625 |
| GO:0006811 | ion transport | 440 | 88 | 60.05 | 7.10E-05 | 0.000788889 |
| GO:0030001 | metal ion transport | 114 | 30 | 15.56 | 0.00021 | 0.0021 |
| GO:0006541 | glutamine metabolic process | 12 | 7 | 1.64 | 0.00036 | 0.003272727 |
| GO:1901606 | alpha-amino acid catabolic process | 11 | 6 | 1.5 | 0.00159 | 0.007956522 |
| GO:0006568 | tryptophan metabolic process | 8 | 5 | 1.09 | 0.00183 | 0.007956522 |
| GO:0006569 | tryptophan catabolic process | 8 | 5 | 1.09 | 0.00183 | 0.007956522 |
| GO:0006586 | indolalkylamine metabolic process | 8 | 5 | 1.09 | 0.00183 | 0.007956522 |
| GO:0009310 | amine catabolic process | 8 | 5 | 1.09 | 0.00183 | 0.007956522 |
| GO:0019441 | tryptophan catabolic process to kynureni... | 8 | 5 | 1.09 | 0.00183 | 0.007956522 |
| GO:0042402 | cellular biogenic amine catabolic proces... | 8 | 5 | 1.09 | 0.00183 | 0.007956522 |
| GO:0042430 | indole-containing compound metabolic pro... | 8 | 5 | 1.09 | 0.00183 | 0.007956522 |
| GO:0042436 | indole-containing compound catabolic pro... | 8 | 5 | 1.09 | 0.00183 | 0.007956522 |
| GO:0042537 | benzene-containing compound metabolic pr... | 8 | 5 | 1.09 | 0.00183 | 0.007956522 |
| GO:0046218 | indolalkylamine catabolic process | 8 | 5 | 1.09 | 0.00183 | 0.007956522 |
| GO:0070189 | kynurenine metabolic process | 8 | 5 | 1.09 | 0.00183 | 0.007956522 |
| GO:0006508 | proteolysis | 638 | 111 | 87.08 | 0.00246 | 0.01025 |
| GO:0015711 | organic anion transport | 28 | 10 | 3.82 | 0.00271 | 0.01084 |
| GO:0009064 | glutamine family amino acid metabolic pr... | 16 | 7 | 2.18 | 0.0032 | 0.011851852 |
| GO:0007009 | plasma membrane organization | 20 | 8 | 2.73 | 0.0032 | 0.011851852 |
| GO:0006576 | cellular biogenic amine metabolic proces... | 10 | 5 | 1.36 | 0.0065 | 0.020967742 |
| GO:0009074 | aromatic amino acid family catabolic pro... | 10 | 5 | 1.36 | 0.0065 | 0.020967742 |
| GO:0009308 | amine metabolic process | 10 | 5 | 1.36 | 0.0065 | 0.020967742 |
| GO:0044106 | cellular amine metabolic process | 10 | 5 | 1.36 | 0.0065 | 0.020967742 |
| GO:0009063 | cellular amino acid catabolic process | 14 | 6 | 1.91 | 0.00717 | 0.021923077 |
| GO:0015908 | fatty acid transport | 7 | 4 | 0.96 | 0.00855 | 0.021923077 |
| GO:0015909 | long-chain fatty acid transport | 7 | 4 | 0.96 | 0.00855 | 0.021923077 |
| GO:0032309 | icosanoid secretion | 7 | 4 | 0.96 | 0.00855 | 0.021923077 |
| GO:0050482 | arachidonic acid secretion | 7 | 4 | 0.96 | 0.00855 | 0.021923077 |
| GO:0071715 | icosanoid transport | 7 | 4 | 0.96 | 0.00855 | 0.021923077 |
| GO:1901571 | fatty acid derivative transport | 7 | 4 | 0.96 | 0.00855 | 0.021923077 |
| GO:1903963 | arachidonate transport | 7 | 4 | 0.96 | 0.00855 | 0.021923077 |
| GO:0009072 | aromatic amino acid family metabolic pro... | 11 | 5 | 1.5 | 0.0106 | 0.0265 |
| GO:0044282 | small molecule catabolic process | 24 | 8 | 3.28 | 0.01136 | 0.027707317 |
| GO:0017121 | plasma membrane phospholipid scrambling | 12 | 5 | 1.64 | 0.01616 | 0.035911111 |
| GO:0034204 | lipid translocation | 12 | 5 | 1.64 | 0.01616 | 0.035911111 |
| GO:0045332 | phospholipid translocation | 12 | 5 | 1.64 | 0.01616 | 0.035911111 |
| GO:0097035 | regulation of membrane lipid distributio... | 12 | 5 | 1.64 | 0.01616 | 0.035911111 |
| GO:0006869 | lipid transport | 36 | 10 | 4.91 | 0.01903 | 0.040411765 |
| GO:0006542 | glutamine biosynthetic process | 5 | 3 | 0.68 | 0.02045 | 0.040411765 |
| GO:0006766 | vitamin metabolic process | 5 | 3 | 0.68 | 0.02045 | 0.040411765 |
| GO:0006767 | water-soluble vitamin metabolic process | 5 | 3 | 0.68 | 0.02045 | 0.040411765 |
| GO:0016054 | organic acid catabolic process | 17 | 6 | 2.32 | 0.02061 | 0.040411765 |
| GO:0046395 | carboxylic acid catabolic process | 17 | 6 | 2.32 | 0.02061 | 0.040411765 |
| GO:0015672 | monovalent inorganic cation transport | 122 | 25 | 16.65 | 0.02221 | 0.041732143 |
| GO:0015718 | monocarboxylic acid transport | 13 | 5 | 1.77 | 0.02337 | 0.041732143 |
| GO:0015849 | organic acid transport | 13 | 5 | 1.77 | 0.02337 | 0.041732143 |
| GO:0042180 | cellular ketone metabolic process | 13 | 5 | 1.77 | 0.02337 | 0.041732143 |
| GO:0046942 | carboxylic acid transport | 13 | 5 | 1.77 | 0.02337 | 0.041732143 |
| GO:0015698 | inorganic anion transport | 23 | 7 | 3.14 | 0.02909 | 0.049644068 |
| GO:0010256 | endomembrane system organization | 28 | 8 | 3.82 | 0.02929 | 0.049644068 |
| GO:0061024 | membrane organization | 28 | 8 | 3.82 | 0.02929 | 0.049644068 |
| *Cellular Component* |  |  |  |  |  |  |
| GO:0016020 | membrane | 1795 | 309 | 258.61 | 5.60E-09 | 5.60E-07 |
| GO:0005576 | extracellular region | 246 | 64 | 35.44 | 3.60E-07 | 1.80E-05 |
| GO:0016021 | integral component of membrane | 1027 | 186 | 147.96 | 1.80E-05 | 0.000575 |
| GO:0031224 | intrinsic component of membrane | 1031 | 186 | 148.54 | 2.30E-05 | 0.000575 |
| GO:0005581 | collagen trimer | 9 | 6 | 1.3 | 0.00049 | 0.0098 |


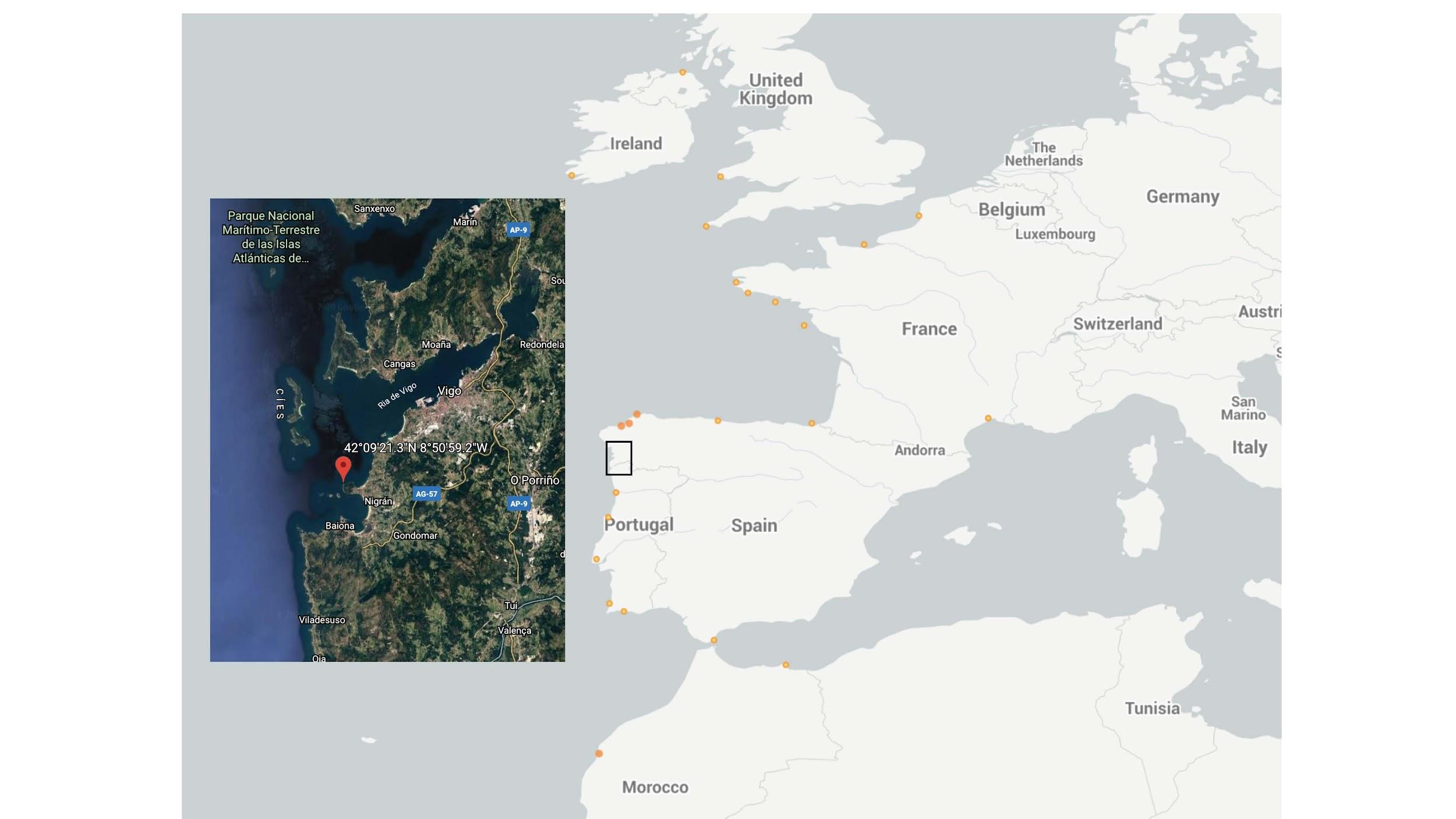


**Figure S1**. Distribution map of *Pollicipes pollicipes* based on the 159 occurrences with location data available (of 372 total occurrences) from GBIF GBIF.org (28 March 2020) GBIF Occurrence Download <https://doi.org/10.15468/dl.qjbfsz>. Inlaid in the distribution map is a magnified view of the boxed location near Vigo, Spain where the specimens were collected for this study.

**
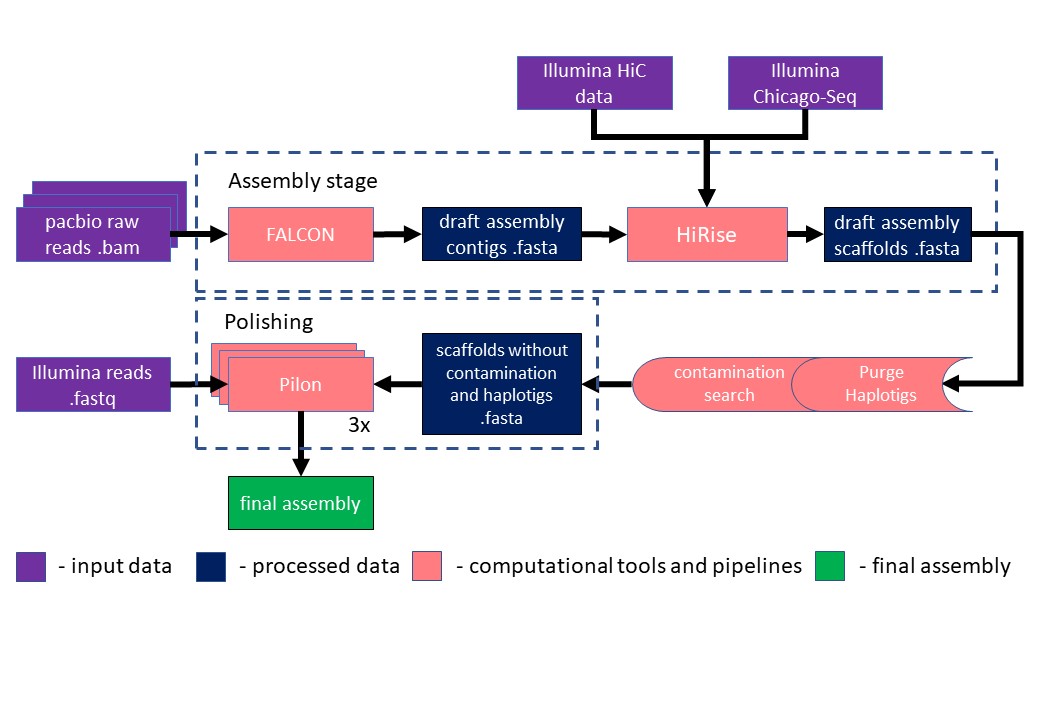
Figure S2**. Genome assembly pipeline.


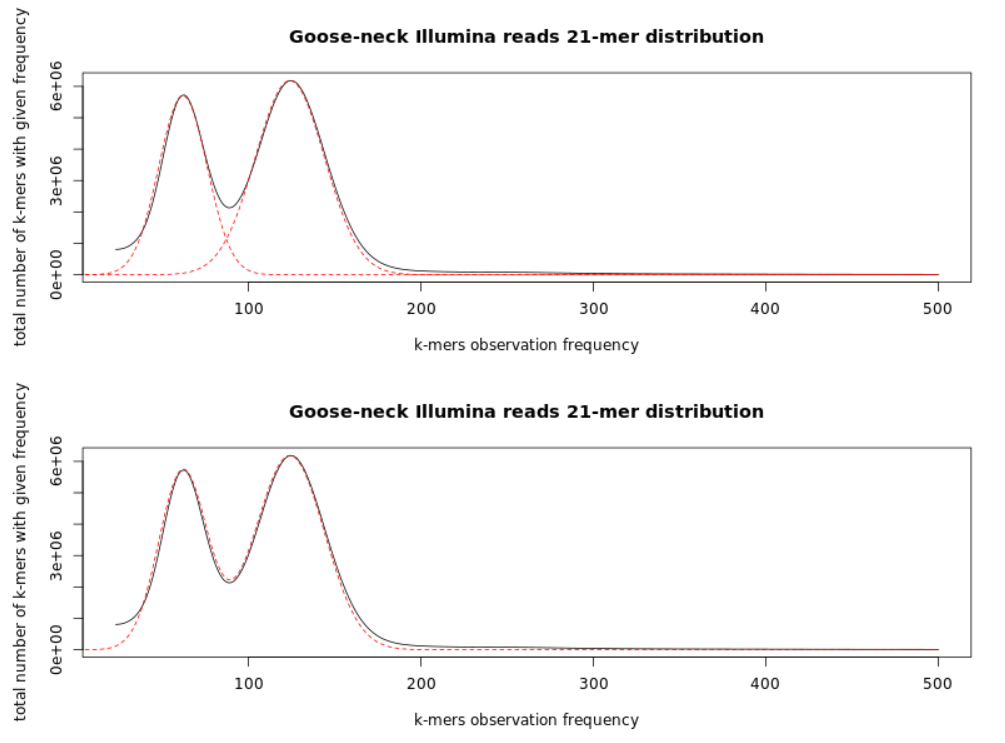


**Figure S3**. Distinct k-mer frequency distribution for the Illumina short reads and two normal distributions mixture for bimodal model red dotted


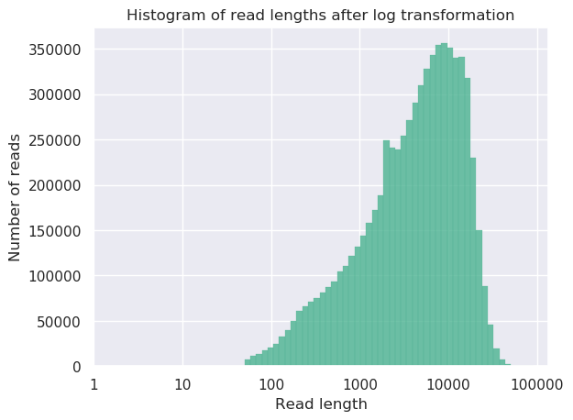
.

**Figure S4**. Histogram of read lengths for Pacbio reads (Nanoplot output).


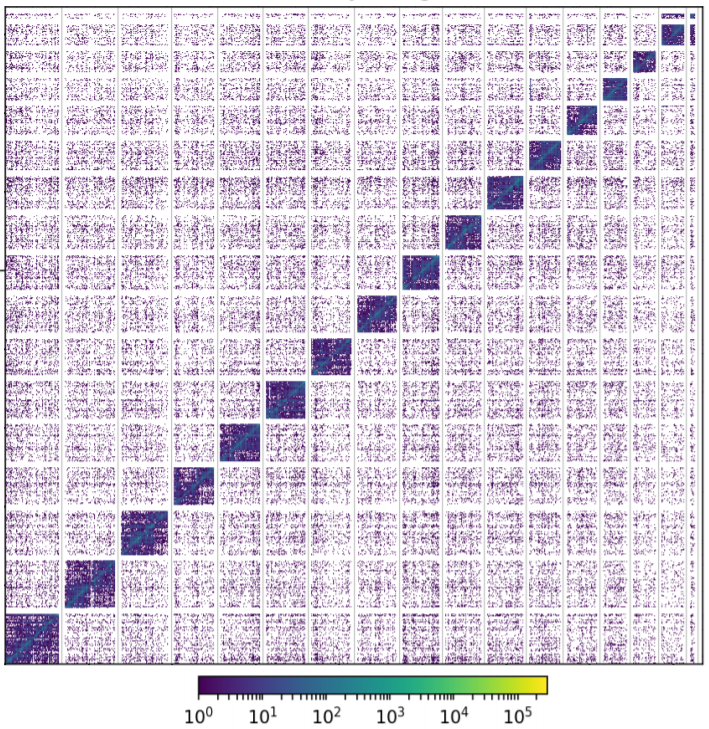


**Figure S5**. HiC contact heat map for scaffolds more than 1Mbp length. Each point color represents the number of HiC read-pairs in this bin. White vertical and horizontal lines have been added to show the borders between scaffolds.


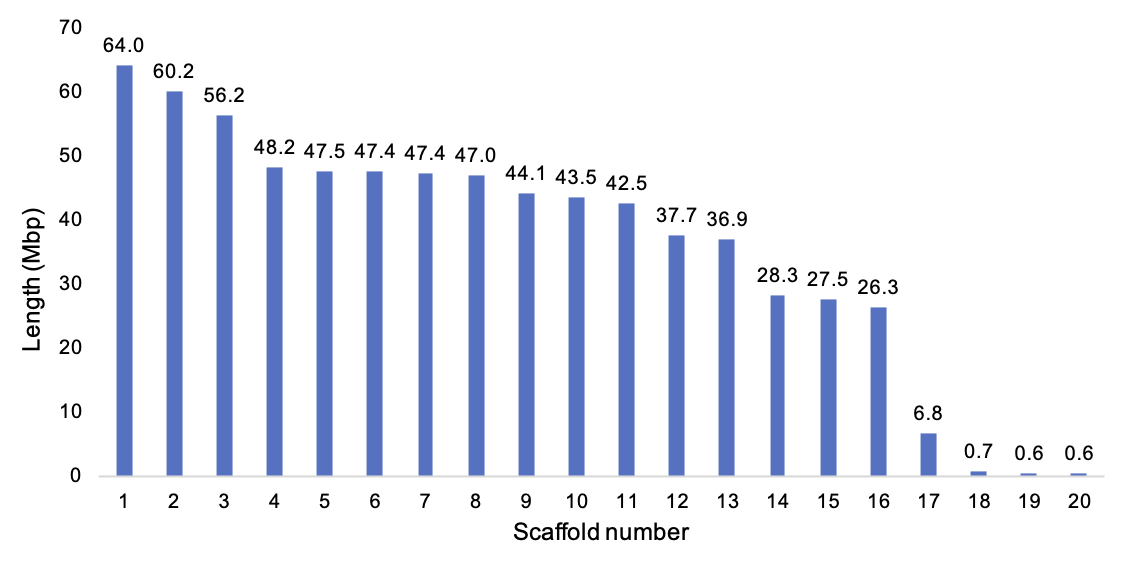


**Figure S6**. Barplot of the sizes of the 20 largest scaffolds.


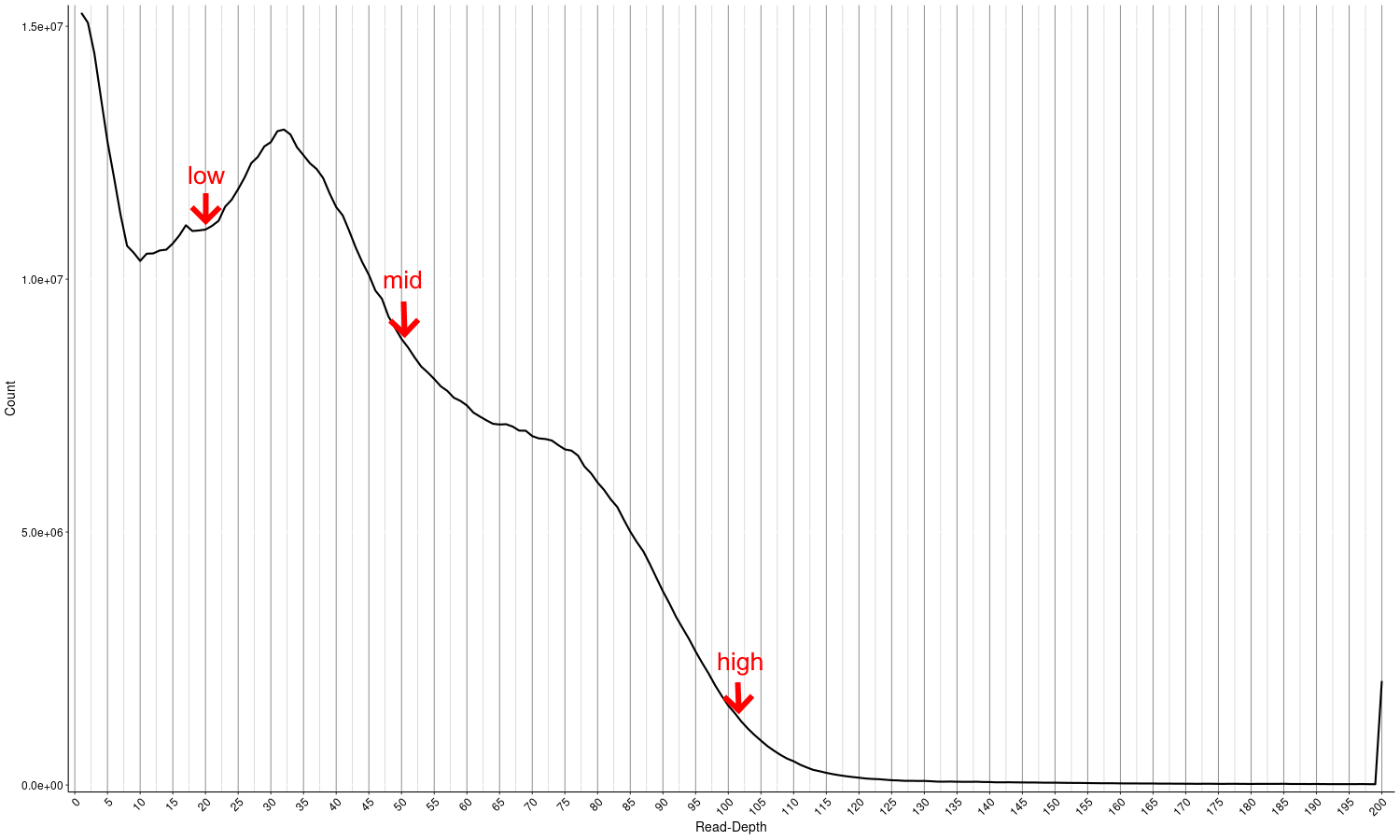


**Figure S7**. Read-depth histogram for Illumina short reads. X-axis - average read depth, Y-axis - number of reads with this depth. Red arrows represent coverage cutoffs for heterozygous and homozygous peaks.


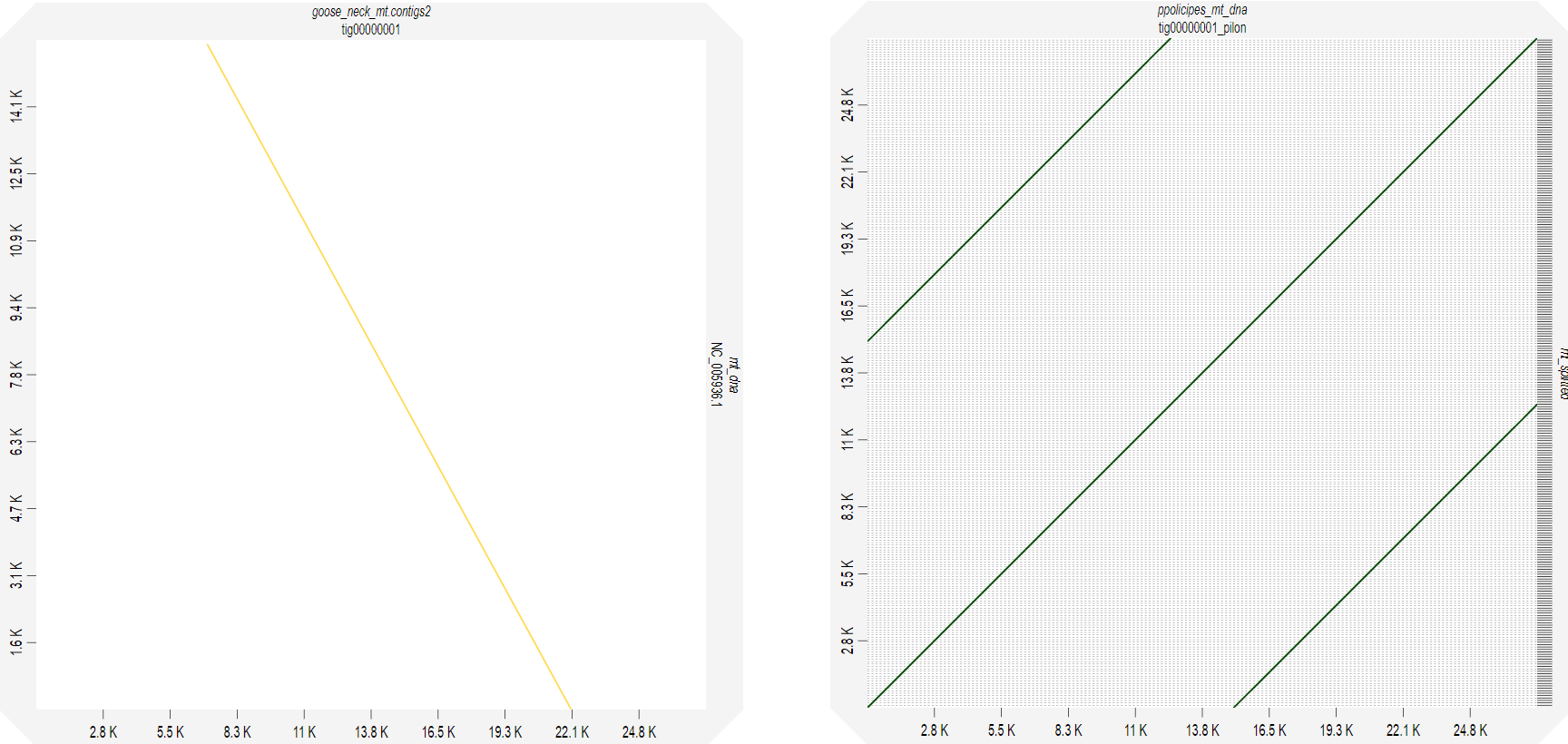


**Figure S8**. Pairwise alignments (a) assembled mitochondrial contig and *P. polymerus* mitochondrial DNA (b) assembled contig aligned to itself.

**
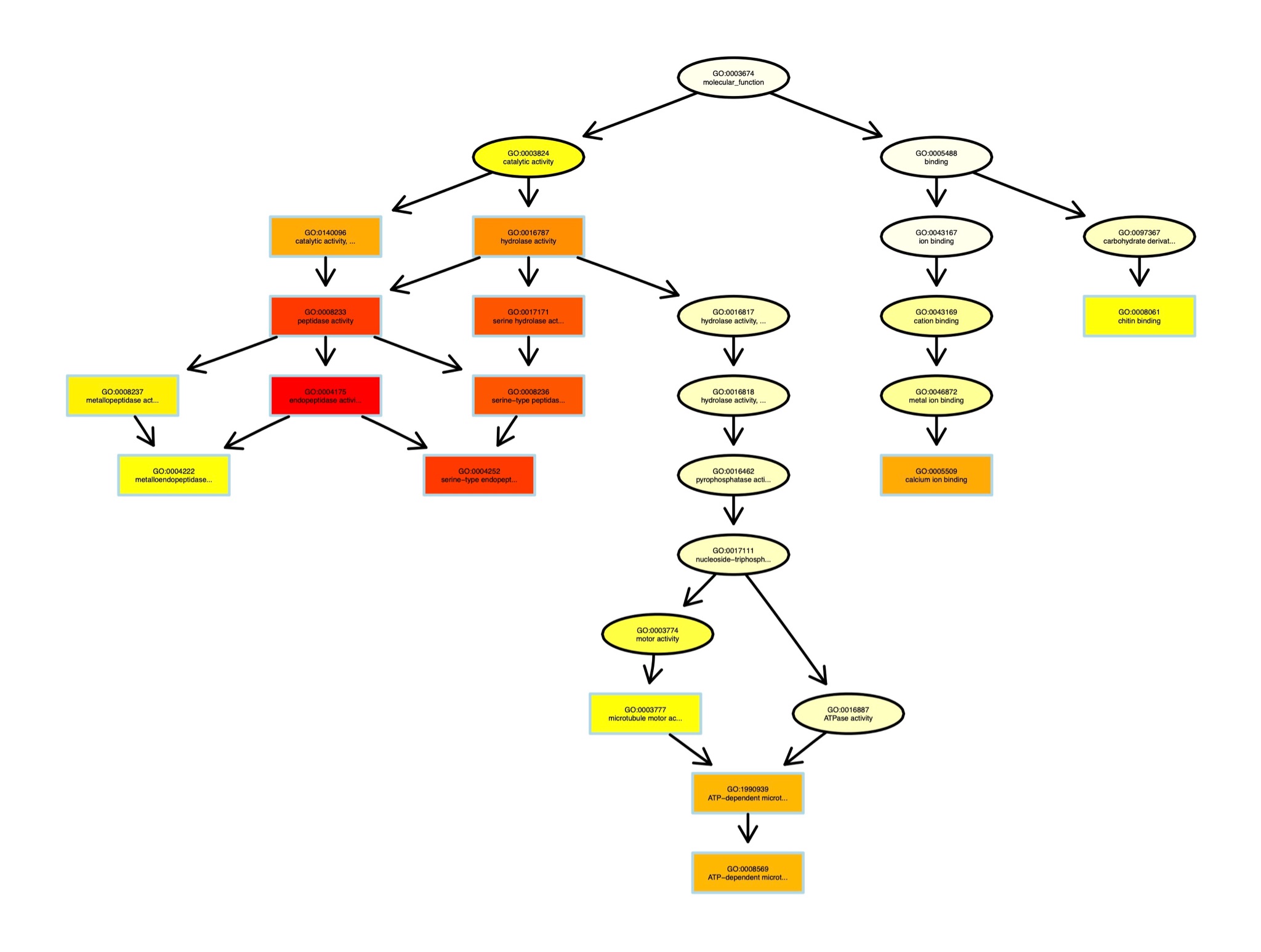
**

**Figure S9.** Nauplius enriched GO graph of Molecular Function for terms q < 0.0001. Boxes indicate significant terms. Box color represents the relative significance, ranging from dark red (most significant) to light yellow (least significant).

**
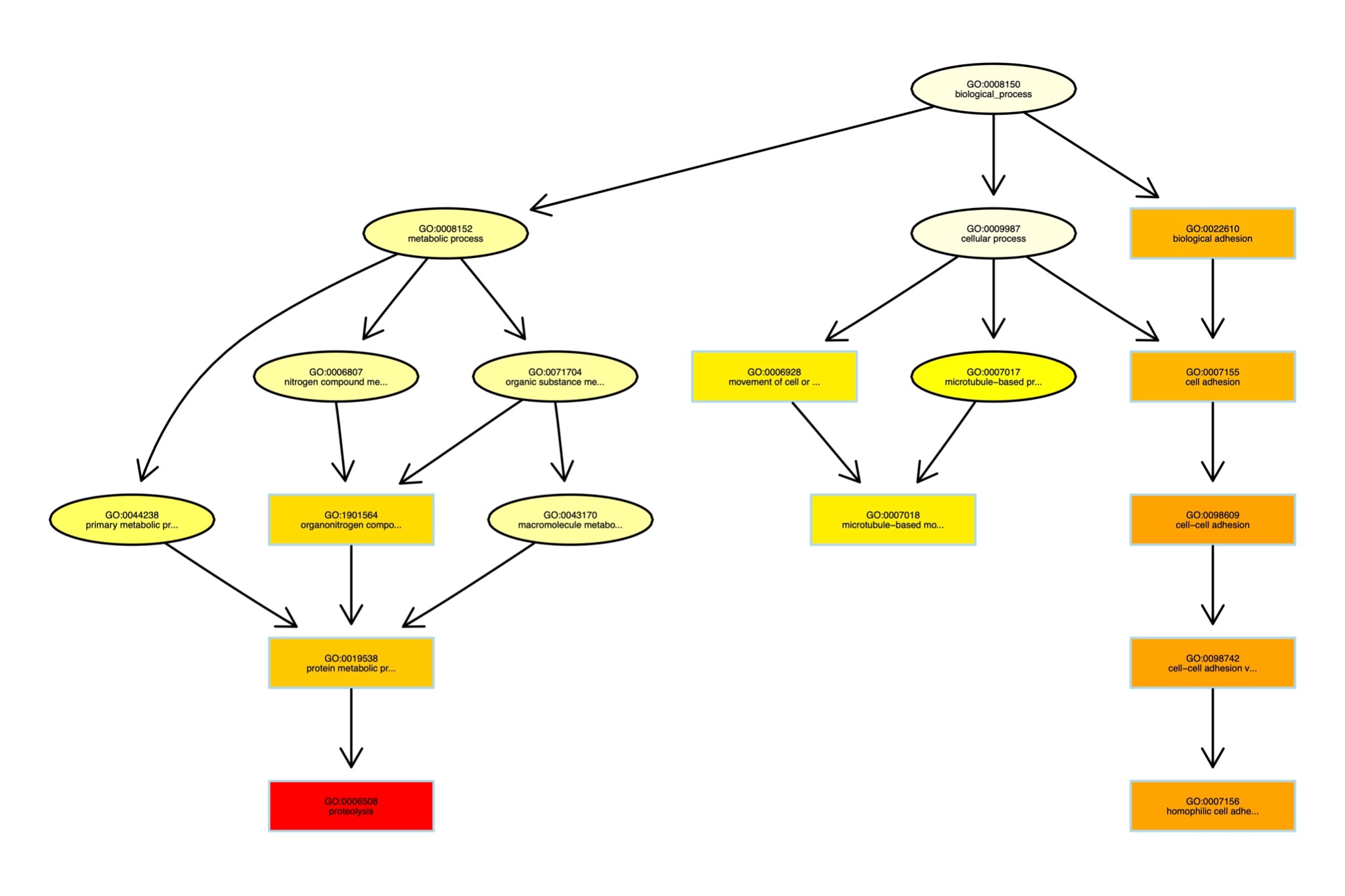
**

**Figure S10.** Nauplius enriched GO graph of Biological Process for terms q < 0.001. Boxes indicate significant terms. Box color represents the relative significance, ranging from dark red (most significant) to light yellow (least significant).

**
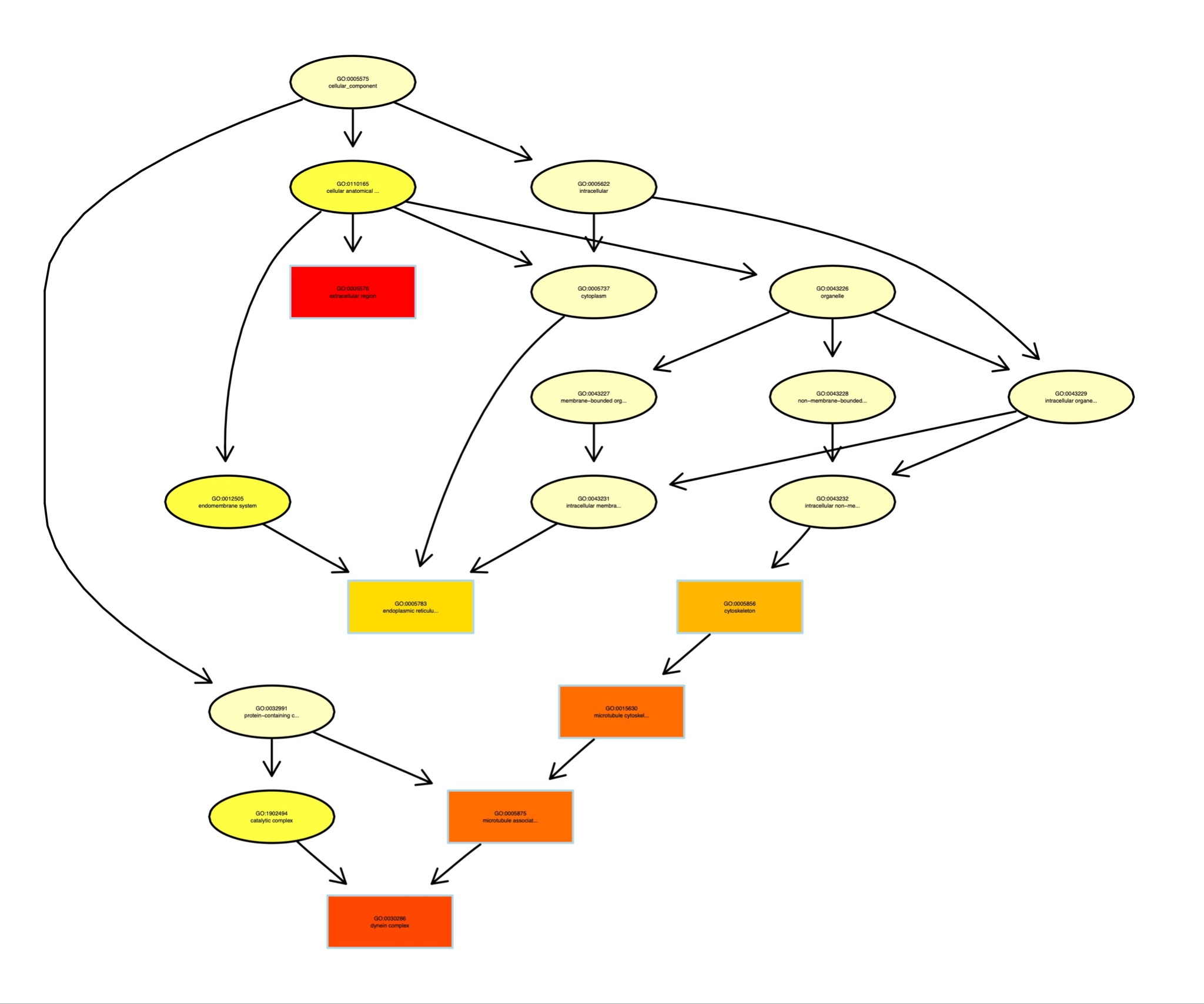
Figure S11.** Nauplius enriched GO graph of Cellular Component for terms q < 0.05. Boxes indicate significant terms. Box color represents the relative significance, ranging from dark red (most significant) to light yellow (least significant).

**
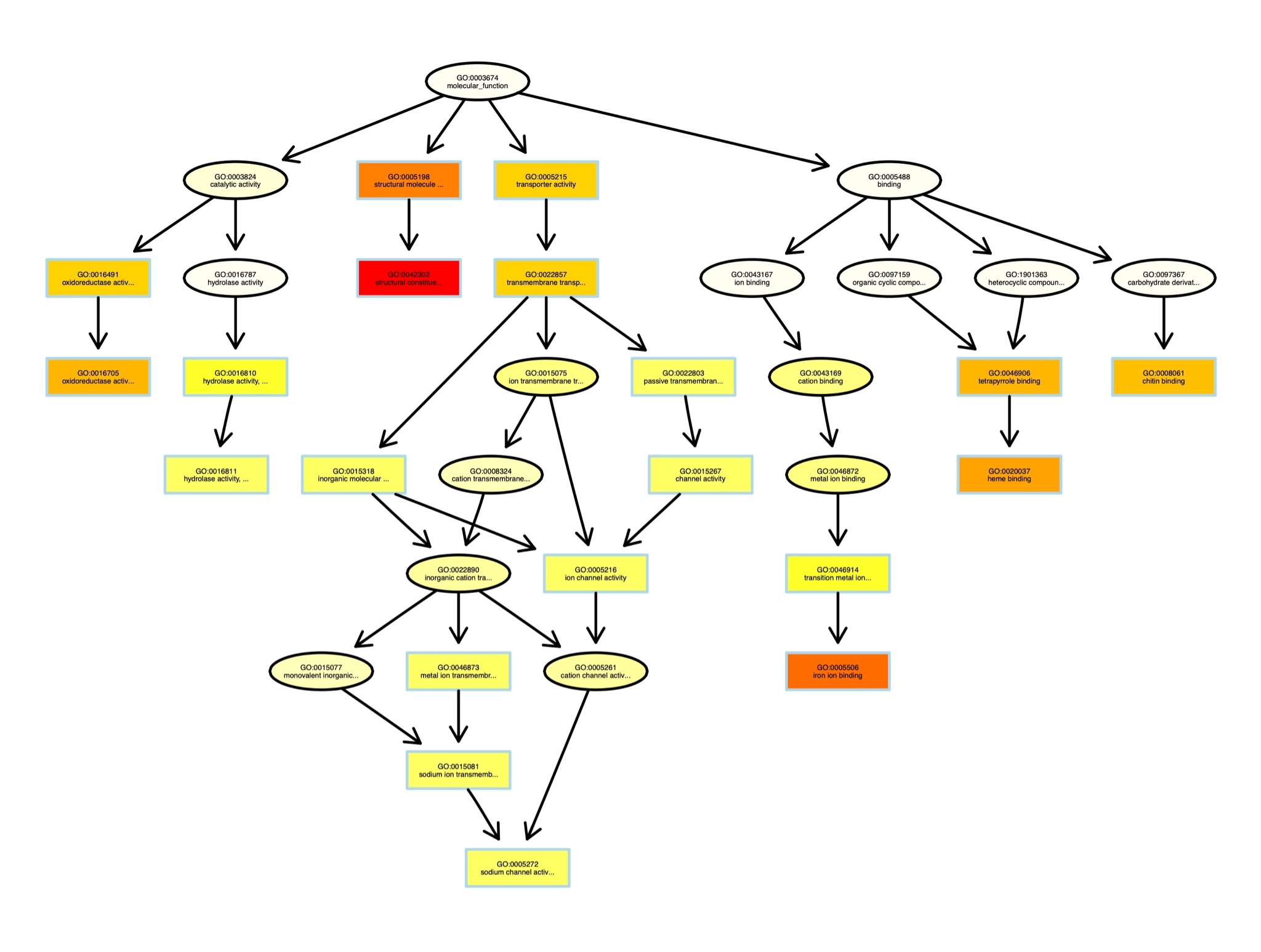
**

**Figure S12.** Adult enriched GO graph of Molecular Function for terms q < 0.0001. Boxes indicate significant terms. Box color represents the relative significance, ranging from dark red (most significant) to light yellow (least significant).

**
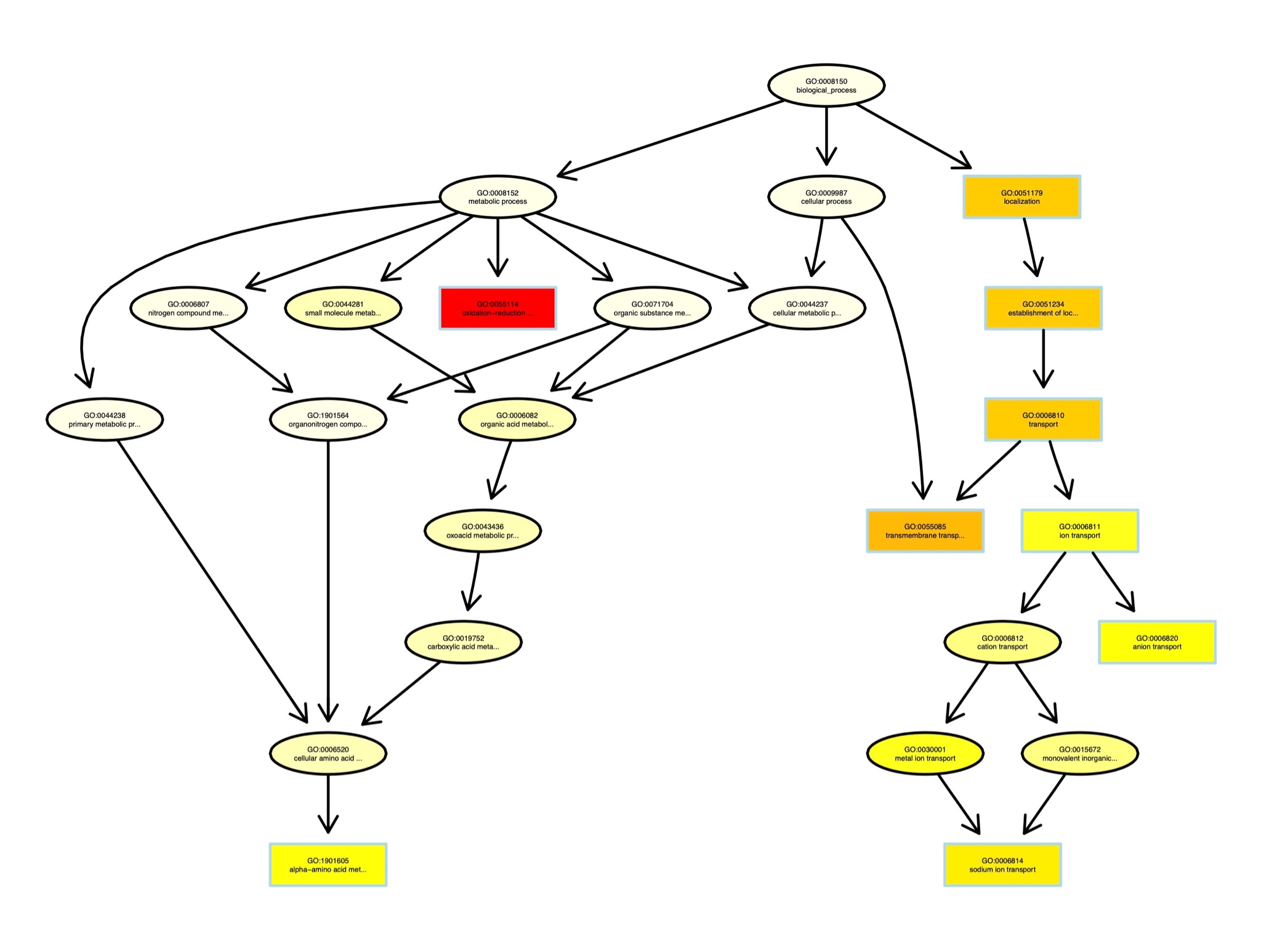
**

**Figure S13.** Adult enriched GO graph of Biological Process for terms q < 0.001. Boxes indicate significant terms. Box color represents the relative significance, ranging from dark red (most significant) to light yellow (least significant).

**
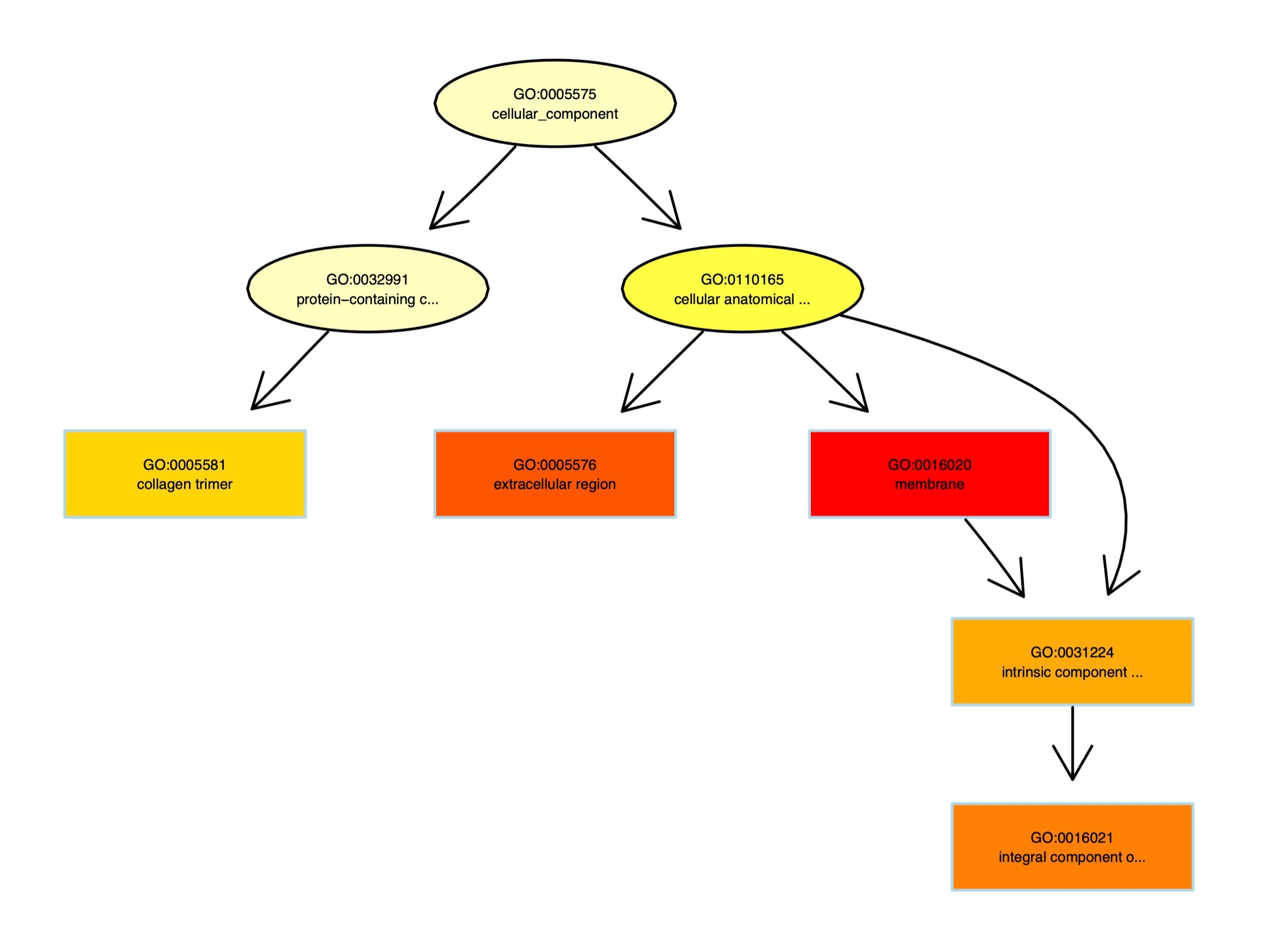
**

**Figure S14.** Adult enriched GO graph of Cellular Component for terms q < 0.05. Boxes indicate significant terms. Box color represents the relative significance, ranging from dark red (most significant) to light yellow (least significant).
