# Supplementary material for: Chromosome-level genome assembly, annotation, and phylogenomics of the gooseneck barnacle Pollicipes pollicipes
Source: Gigascience. 2022 Mar 12;11:giac021. doi: 10.1093/gigascience/giac021 (PMC8917513; doi:10.1093/gigascience/giac021)
Supplement: giac021_GIGA-D-21-00365_Revision_1 [file giac021_giga-d-21-00365_revision_1.pdf]

GigaScience

Chromosome-level genome assembly, annotation, and phylogenomics of the gooseneck barnacle Pollicipes pollicipes

--Manuscript Draft--

**Manuscript Number:**	GIGA-D-21-00365R1
**Full Title:**	Chromosome-level genome assembly, annotation, and phylogenomics of the gooseneck barnacle Pollicipes pollicipes
**Article Type:**	Data Note
**Funding Information:**	US National Science Foundation (2010898) — Dr. James P. Bernot
**Abstract:**	Background:<br>The barnacles are a group of more than 2,000 species that have fascinated biologists, including Darwin, for centuries. Their lifestyles are extremely diverse from free-swimming larvae to sessile adults, and even root-like endoparasites. Barnacles also have a major economic impact with hundreds of millions of dollars of losses annually due to biofouling. However, genomic resources for crustaceans, and barnacles in particular, are lacking.<br><br>Results:<br>Using 62x PacBio coverage, 189x Illumina WGS coverage, 203x HiC coverage, and 69x CHi-C coverage, we produced a chromosome-level genome assembly of the gooseneck barnacle Pollicipes pollicipes. The P. pollicipes genome is 770 Mbp long and its assembly is one of the most contiguous and complete crustacean genomes available, with a scaffold N50 of 47 Mbp and 90.5% of the BUSCO Arthropoda gene set. Using the genome annotation produced here along with transcriptomes of 13 other barnacle species, we completed phylogenomic analyses on a nearly 2 million amino acid alignment. Contrary to previous studies, our phylogenies suggest the Pollicipedomorpha is monophyletic and sister to the Balanomorpha, which alters our understanding of barnacle larval evolution and suggests homoplasy in a number of naupliar characters. We also compared transcriptomes of P. pollicipes nauplius larvae and adults and found that nearly one half of the genes in the genome are differentially expressed, highlighting the vastly different transcriptomes of larvae and adult gooseneck barnacles. Annotation of the genes with KEGG and GO terms reveals these stages exhibit many differences including cuticle binding, chitin binding, microtubule motor activity, and membrane adhesion.<br><br>Conclusion:<br>This study provides high quality genomic resources for a key group of crustaceans. This is especially valuable given the roles P. pollicipes plays in European fisheries, as a sentinel species for coastal ecosystems, and as a model for studying barnacle adhesion as well as its key position in the barnacle tree of life. A combination of genomic, phylogenetic, and transcriptomic analyses here provides valuable insights into the evolution and development of barnacles.
**Corresponding Author:**	Keith A Crandall, PhD<br>George Washington University<br>Washington, DC UNITED STATES
**Corresponding Author Secondary Information:**	
**Corresponding Author's Institution:**	George Washington University
**Corresponding Author's Secondary Institution:**	
**First Author:**	James P. Bernot, PhD
**First Author Secondary Information:**	
**Order of Authors:**	James P. Bernot, PhD

Powered by Editorial Manager® and ProduXion Manager® from Aries Systems Corporation

	Pavel Avdeyev, PhD
	Anton Zamyatin
	Niklas Dreyer
	Nikita Alexeev
	Marcos Pérez-Losada
	Keith A Crandall, PhD
**Order of Authors Secondary Information:**	
**Response to Reviewers:**	See attached file
**Additional Information:**	
**Question**	**Response**
Are you submitting this manuscript to a special series or article collection?	No
**Experimental design and statistics**<br><br>Full details of the experimental design and statistical methods used should be given in the Methods section, as detailed in our Minimum Standards Reporting Checklist. Information essential to interpreting the data presented should be made available in the figure legends.<br><br>Have you included all the information requested in your manuscript?	Yes
**Resources**<br><br>A description of all resources used, including antibodies, cell lines, animals and software tools, with enough information to allow them to be uniquely identified, should be included in the Methods section. Authors are strongly encouraged to cite Research Resource Identifiers (RRIDs) for antibodies, model organisms and tools, where possible.<br><br>Have you included the information requested as detailed in our Minimum Standards Reporting Checklist?	Yes
**Availability of data and materials**	Yes

All datasets and code on which the conclusions of the paper rely must be either included in your submission or deposited in publicly available repositories (where available and ethically appropriate), referencing such data using a unique identifier in the references and in the "Availability of Data and Materials" section of your manuscript.<br><br>Have you have met the above requirement as detailed in our Minimum Standards Reporting Checklist?	

Powered by Editorial Manager® and ProduXion Manager® from Aries Systems Corporation

Manuscript Click here to access/download;Manuscript;BarnacleGenomeRevised09JAN22

DATA NOTE

Chromosome-level genome assembly, annotation, and phylogenomics of the gooseneck barnacle *Pollicipes pollicipes*

James P. Bernot[1,2+] [https://orcid.org/0000-0002-1769-8631], Pavel Avdeyev[1+] [https://orcid.org/0000-0002-7953-6259], Anton Zamyatin[3] [https://orcid.org/0000-0001-5402-1796], Niklas Dreyer[4,5,6,7] [https://orcid.org/0000-0002-1391-1642], Nikita Alexeev[3] [https://orcid.org/0000-0003-3415-9565], Marcos Pérez-Losada[1,8,9] [https://orcid.org/0000-0002-2585-4657], and Keith A. Crandall[1,2,8*] [https://orcid.org/0000-0002-0836-3389]

[1]Computational Biology Institute, Milken Institute School of Public Health, The George Washington University, Washington, DC 20052, USA
[2]Department of Invertebrate Zoology, US National Museum of Natural History, Smithsonian Institution, Washington DC 20012, USA
[3]Computer Technologies Laboratory, ITMO University, Saint-Petersburg, Russia
[4]Department of Life Science, National Taiwan Normal University, Taiwan
[5]Biodiversity Program, Taiwan International Graduate Program, Academia Sinica, Taipei
[6]Biodiversity Research Center, Academia Sinica, Taipei 115, Taiwan
[7]Natural History Museum of Denmark, University of Copenhagen, Universitetsparken 15, DK-2100, Denmark
[8]Department of Biostatistics & Bioinformatics, Milken Institute School of Public Health, The George Washington University, Washington, DC 20052, USA
[9]CIBIO-InBIO, Centro de Investigação em Biodiversidade e Recursos Genéticos, Universidade do Porto, Campus Agrário de Vairão, Vairão, Portugal

[+]Equal contribution
[*]Correspondence address. Keith Crandall, Computational Biology Institute, 800 22nd Street NW, Suite 7000, Washington, DC 20052; kcrandall@gwu.edu, https://orcid.org/0000-0002-0836-3389

Abstract

Background:

The barnacles are a group of more than 2,000 species that have fascinated biologists, including Darwin, for centuries. Their lifestyles are extremely diverse from free-swimming larvae to sessile adults, and even root-like endoparasites. Barnacles also have a major economic impact with hundreds of millions of dollars of losses annually due to biofouling. However, genomic resources for crustaceans, and barnacles in particular, are lacking.

Results:

Using 62x PacBio coverage, 189x Illumina WGS coverage, 203x HiC coverage, and 69x CHi-C coverage, we produced a chromosome-level genome assembly of the gooseneck barnacle *Pollicipes pollicipes*. The *P. pollicipes* genome is 770 Mbp long and its assembly is one of the most contiguous and complete crustacean genomes available, with a scaffold N50 of 47 Mbp and 90.5% of the BUSCO Arthropoda gene set. Using the genome annotation produced here along with transcriptomes of 13 other barnacle species, we completed phylogenomic analyses on a nearly 2 million amino acid alignment. Contrary to previous studies, our phylogenies suggest the Pollicipedomorpha is monophyletic and sister to the Balanomorpha, which alters our understanding of barnacle larval evolution and suggests homoplasy in a number of naupliar characters. We also compared transcriptomes of *P. pollicipes* nauplius larvae and adults and found that nearly one half of the genes in the genome are differentially expressed, highlighting the vastly different transcriptomes of larvae and adult gooseneck barnacles. Annotation of the genes with KEGG and GO terms reveals these stages exhibit many differences including cuticle binding, chitin binding, microtubule motor activity, and membrane adhesion.

Conclusion:

This study provides high quality genomic resources for a key group of crustaceans. This is especially valuable given the roles *P. pollicipes* plays in European fisheries, as a

sentinel species for coastal ecosystems, and as a model for studying barnacle adhesion as well as its key position in the barnacle tree of life. A combination of genomic, phylogenetic, and transcriptomic analyses here provides valuable insights into the evolution and development of barnacles.

Keywords

Barnacle, genome, larval evolution, assembly, annotation, crustacea, phylogeny, Pollicipes

Data Description

Context

The Earth BioGenome Project has the ambitious goal of sequencing a high-quality genome from each described eukaryotic species on the planet [1]. This goal can be especially difficult for invertebrate species because of the fundamental lack of available reference genomes [2]. The Pancrustacea (‘Crustacea’ + Hexapoda) is the most biologically diverse and species-rich animal taxon on the planet, containing over 1.2 million described species. Even excluding the hyperdiverse insects, the ‘Crustacea’ contains more than 60,000 described species [3], including numerous taxa of economic importance as food resources, fouling organisms, keystone species, and model organisms for biological research. Despite their importance, there is very little genomic reference data available; fewer than 50 species have available genome sequences (42 species in NCBI), and only seven assemblies approach chromosome-level contiguity.

The Thecostraca is a pancrustacean taxon containing the familiar and ubiquitous barnacles and a number of parasitic lineages comprising the Ascothoracida [4], Rhizocephala [5], and the enigmatic Facetotecta, for which adult stages have not yet been found [6]. The Cirripedia, or barnacles, are an almost entirely marine group of over 2,000 species with a rich fossil record [7]. They display diverse morphologies and biologies including: i) free-swimming, plankton-feeding nauplius larvae (Fig. 1A, B), ii) nonfeeding, settlement-larvae called cyprids (Fig. 1C), and iii) sessile, shell-plated, suspension-feeding adults (Fig. 1D-F). Such diversity has made them model organisms in larval biology, morphology, sexual evolution, and intertidal ecology [7]. Barnacles have been the focus of evolutionary research since Darwin himself studied the group intently [8–11]. They are also notorious for fouling man-made objects, particularly ships and docks. Fouling barnacles are responsible for hundreds of millions of dollars in economic losses each year, primarily from fuel costs due to increased drag on ship hulls [12]; Schultz et al. [12] estimated that the US Navy alone overspends $180–$500 million each year due to fouling.

The gooseneck barnacle *Pollicipes pollicipes* (Gmelin, 1791 [in Gmelin, 1788-1792]) is a member of the Pollicipedomorpha (Thoracicalcarea), a new order [7] of stalked barnacles with a body encased by a wall of articulating, calcified shell plates

atop an elongate peduncle (Fig. 1D). The order includes four genera (*Anelasma*, *Pollicipes*, *Capitulum*, and *Lithotrya*) that have a close phylogenetic affinity in molecular analyses. Many studies have placed them near the Balanomorpha and Verrucamorpha [13], but their phylogenetic position and even the monophyly of the order are still under debate - particularly in studies using adult and/or larval character matrices (Fig. 2A) [14–17].

Like in many invertebrate taxa, genomic resources for crustaceans are lacking, which has hindered the study of genome and phenotypic evolution, and the estimation of robust phylogenies [2]. The gooseneck barnacle *P. pollicipes* is a particularly good choice for genomic sequencing given its importance in European fisheries [18] and role as a sentinel species for coastal marine ecosystems [19]; *P. pollicipes* is also a model for studying barnacle adhesion mechanisms and engineering of new adhesive materials [20] and occupies a key phylogenetic position in the barnacle tree of life [7]. Furthermore, our barnacle genome project may represent an exemplar for future invertebrate genome sequencing, assembly, and annotation approaches in the Earth BioGenome Project, since it includes abundant, high quality data, robust methods for assembly and annotation, appropriate vouchering of specimens used for genome sequencing, and metadata associated with the specimens - all with discoverable identifiers, allowing for an ‘extended specimen’ [21,22]. Therefore, the goal of this study is to sequence, assemble, and annotate the full genome of the gooseneck barnacle *P. pollicipes*. We think this barnacle species is a great example of this important group of organisms and our genome approach regarding depth, diversity of data (extended specimen), linkage of data, and FAIRness [23–25], aligns well with the goals of the Earth BioGenome Project.

Table 1. Genome sequencing mean coverage based on raw data (prior to QA/QC) and genome size of 770 Mbp.

Data type	Raw data (bp)	Coverage
PacBio	47,984,705,480	62x
Illumina WGS	150,600,000,000	196x

HiC	156,600,000,000	203x
CHi-C	53,100,000,000	69x

Methods and materials

Genomic Sample Collection

Samples for this study were collected by hand at 42˚09’21.2”N, 8˚50’59.2”W in Punta Meda, Nigran, Pontevedra, Spain (Fig. S1), preserved in 95% ethanol and stored at -80°C. Multiple individuals were collected to allow some to be used as vouchers (see Fig. 1), some for transcriptomics/proteomics, and some for genome sequencing. Voucher specimens are deposited in the US National Museum of Natural History, Smithsonian Institution under accession numbers USNM 1622609 (hologenophore) and USNM 1622610 (paragenophore lot).

Larval development and life cycle

We reared larvae and adults of *P. pollicipes* and examined them with macrophotography, light and scanning electron microscopy to provide an overview of the life cycle and key morphological features (Fig. 1). The adult specimens used for larval culturing and adult internal anatomy were collected in Quiberon Peninsula on the South coast of Brittany, France. Adult *P. pollicipes* groups or solitary individuals were carefully removed on a piece of substratum/rock using a hammer and chisel. The specimens were transported back to the lab wrapped in a wet towel and cultured at the University of Wales, Swansea, United Kingdom. The specimens were housed in laboratory aquaria with running sea-water, and egg-lamellae were removed and cultured separately in filtered sea-water and antibiotics (50 units penicillin and 0.05 mg streptomycin sulphate/mL water). Upon hatching from the eggs, approximately 50 nauplius stage 1 specimens (Fig. 1A) were attracted with a light-source and fixed in 10% sea-water-based formalin. The remaining nauplii were reared under a density of 5 x 10^2 L^{-1} and fed with the flagellate algae *Isochrysis galbana* at a concentration of 100 x 10^4 cells ml^{-1} at 20°C. Upon reaching nauplius stage 3, we switched diet to the large dinoflagellate *Prorocentrum micans*. Finally, 50 last stage nauplii (nauplius 6) and 10 cyprid larvae were fixed in formalin for examination with scanning electron microscopy

(SEM). One adult specimen was carefully dissected along its midline with a tweezer (Fig. 1E). Specimens for SEM-preparation were placed in filtered ddH_20 in small glass vessels. We changed the water thrice and left the larvae overnight. Larvae were then gradually dehydrated through an alcohol series (thrice each; 10%-100%). Specimens were then critical point dried with liquid CO_2, sputter coated with an alloy of platinum and palladium, placed on their lateral side on SEM-stubs with a hair brow wig taped to a stick and finally photographed in a JEOL-JSM-6335F fitted with a field emission gun.

Genome Sequencing

PacBio library preparation and sequencing

DNA was extracted from ethanol preserved specimens following Dovetail Genomics protocols. DNA samples were quantified using a Qubit 2.0 Fluorometer (Life Technologies, Carlsbad, CA, USA). Fragments of approximately 20 Kbp were selected for library preparation using SMRTbell Template Prep Kit 1.0 (PacBio, Menlo Park, CA, USA) following the manufacturer recommended protocol. The pooled library was bound to polymerase using the Sequel Binding Kit 2.0 (PacBio) and loaded onto a PacBio Sequel using the MagBead Kit V2 (PacBio). Sequencing was performed on 14 PacBio Sequel SMRT cells using Instrument Control Software v5.0.0.6235, Primary Analysis Software v5.0.0.6236 and SMRT Link v5.0.0.6792. The resulting PacBio library contained 7.01 M reads with mean read length 6.84 Kbp, median 4.74 Kbp, and read length N50 12.34 Kbp for an average of 62.18x coverage (Fig. S4). Statistics were gathered by the NanoStat tool from NanoPack package [26].

CHi-C library preparation and sequencing

Two Capture Hi-C (CHi-C) libraries were prepared as described previously [27]. Briefly, for each library, ~500ng of HMW gDNA (mean fragment length = 50 kbp) was reconstituted into chromatin *in vitro* and fixed with formaldehyde. Fixed chromatin was digested with DpnII, the 5' overhangs filled in with biotinylated nucleotides, and then free blunt ends were ligated. After ligation, crosslinks were reversed and the DNA purified from protein. Purified DNA was treated to remove biotin that was not internal to ligated fragments. The DNA was then sheared to ~350 bp mean fragment size and

sequencing libraries were generated using NEBNext Ultra enzymes and Illumina-compatible adapters. Biotin-containing fragments were isolated using streptavidin beads before PCR enrichment of each library. The libraries were sequenced on an Illumina HiSeqX. The number and length of read pairs produced for each library was: 87 million, 2x150 bp for library 1 and 90 million, 2x150 bp for library 2. Together, these CHi-C libraries provided 68.96x physical coverage of the genome (1-50 Kbp).

Dovetail HiC library preparation and sequencing

Two Dovetail HiC libraries were prepared in a similar manner as described previously [28]. Briefly, for each library, chromatin was fixed in place in the nucleus with formaldehyde and then extracted. Fixed chromatin was digested with DpnII, the 5' overhangs filled in with biotinylated nucleotides, and free blunt ends were then ligated. After ligation, crosslinks were reversed and the DNA purified from protein. Purified DNA was treated to remove biotin that was not internal to ligated fragments. The DNA was then sheared to ~350 bp mean fragment size and sequencing libraries were generated using NEBNext Ultra enzymes and Illumina-compatible adapters. Biotin-containing fragments were isolated using streptavidin beads before PCR enrichment of each library. The libraries were sequenced on an Illumina HiSeqX. The number and length of read pairs produced for each library was: 262 million, 2x150 bp for library 1 and 260 million, 2x150 bp for library 2. Together, these Dovetail HiC library reads provided 203.37x physical coverage of the genome (1-50 Kbp).

Illumina library preparation and sequencing

Approximately 10 µl of the remaining DNA extracted as described above for PacBio sequencing was used for Illumina short read sequencing. DNA samples were quantified using a Qubit 2.0 Fluorometer and libraries prepared using the standard Illumina DNA Prep protocol. The 10 µl of high molecular weight DNA were added to a PCR plate, combined with 20 µl of nuclease free water for a total volume of 30 µl, vortexed, and combined with the tagmentation master mix, sealed, and placed on a thermocycler (55°C for 15 minutes, held at 10°C). Beads were resuspended by adding 10 µl of TSB, placed on a magnetic stand for 3 minutes, and the supernatant removed

and discarded. The sample was washed twice with 100 µl TWB, allowing the sample to clear on the magnetic stand for 3 minutes each time. Another 100 µl of TWB was added and the tagmented DNA underwent limited PCR amplification to add dual index adapters (i7 and i5) following standard Illumina protocols. The libraries were then cleaned using the standard Illumina DNA double sided bead purification procedure. The final libraries were used for 150 bp paired-end sequencing using a NextSeq High Output 300 cycle kit on an Illumina NextSeq 500 at the GWU Genomics Core.

Quality control showed that the Illumina library contains 1,043.2 million sequences (502 million PE reads) with average duplication 26.6% and 145 bp average read length. To exclude possible sequencing errors and eliminate phiX contamination, we filtered the library with quality cutoff 10 using the DADA2 pipeline [29]. The filtered library contained 904.6 million sequences with 25.3% read duplication.

Table 2. *Pollicipes pollicipes* genome assembly statistics

Total assembly length	770 Mbp
GC (%)	52.31%
Largest scaffold	64,043,775 bp
Scaffold N50	47,009,503 bp
Scaffold N75	37,696,644 bp
Scaffold L50	8
Scaffold L75	12
Number contigs	1,075
Contig N50	95,549 bp
Contig N75	22,233 bp
Contig N90	16,125 bp
Complete BUSCO Arthropoda	90.5%
Single BUSCO Arthropoda	69.4%

Duplicated BUSCO Arthropoda	21.1%
Fragmented BUSCO Arthropoda	3.3%
Missing BUSCO Arthropoda	6.2%
Complete BUSCO Metazoa	91.2%
Single BUSCO Metazoa	67.8%
Duplicated BUSCO Metazoa	23.4%
Fragmented BUSCO Metazoa	4.4%
Missing BUSCO Metazoa	4.4%

Genome Assembly

The genome assembly pipeline is shown in Figure S2. The initial genome assembly was performed using the FALCON [30] v1.8.8 [31]. First, 59.9x whole-genome, single-molecule, real-time sequencing (SMRT) data were used as input to the traditional FALCON pipeline using a length cut-off that corresponds to 50x coverage of data during the initial error-correcting stage. This resulted in 4 million error corrected reads with an N50 read length equal of 9 Kbp covering 46.8x of the genome. The error-corrected reads were processed by the overlap portion of the FALCON pipeline. The aligned reads were assembled in the third stage of FALCON into 18,083 contigs. Finally, the assembly was polished with the Arrow algorithm from SMRT Link 5.0.1 using the original raw reads. The obtained assembly was assessed by QUAST-LG [32] and BUSCO v5.2.2 [33]. For the AUGUSTUS (v3.2) tool [34] in the BUSCO pipeline, we used a gene model pre-trained on the *Drosophila melanogaster* genome.

Mitochondrial genome assembly

We assembled the *P. pollicipes* mitochondrial genome using a modified version of the Vertebrate Genome Project mitoassembly pipeline [35]. Briefly, we aligned all PacBio reads to the *P. polymerus* mitochondrial genome (NCBI Reference Sequence: NC_005936.1) [36] using BlasR [37] v5.3.3 [38]. We then used CANU [39] v2.0 to assemble 2,388 extracted reads into a single circular contig 27.8 Kbp long. The

resulting contigs were aligned against the *P. polymerus* mitochondrial genome and itself (see Fig. S8). Dot plots indicated the cyclic DNA repetitiveness with approximately 1.9 copies of the full mitochondrial genome is present in the contig. We trimmed the contig to keep a single copy of the mitochondrial genome. The obtained sequence was further polished with approximately 6,000x Illumina coverage using Pilon [40] v1.23 [41]. Pilon confirmed 99.9% nucleotide bases and fixed 9 insertions, 2 deletions, and 1 SNP.

Genome Scaffolding

The initial Falcon assembly, CHi-C reads, and Hi-C reads were used as input in HiRise, a Dovetail Genomics software pipeline for using proximity ligation data to scaffold genome assemblies. An iterative analysis was conducted. First, CHi-C library sequences were aligned to the Falcon draft assembly using a modified SNAP [42] read mapper [43]. The separations of CHi-C read pairs mapped within draft scaffolds were analyzed by HiRise to produce a likelihood model for genomic distance between read pairs. The model was used to identify and break suspected incorrect joinings, to score prospective joins, and to make joins above a threshold. The resulting assembly contained 8,768 contigs of 906 Mbp total length with an N50 equals 660.8 Kbp. After aligning and scaffolding CHi-C data, HiC library sequences were aligned and scaffolded with the same method. At the last stage, the original PacBio long reads were used to close gaps between contigs. Figure S5 shows the resulting contact heat map for the resulting assembly of the *P. pollicipes* genome produced by HiRise.

Genome Curation

Haplotype Filtration

Given the higher rate of core gene duplications (21.1% of Arthropoda genes [Table 2]), we suspected the presence of haplotypes in the scaffolded assembly. Therefore, we classified all contigs with PurgeHaplotigs [44] v1.0.0 [45] into primary contigs, haplotigs, repeat contigs, and assembly artifacts based on the read-depth analysis as follows. Read-depth histograms were produced for the draft assembly (see Fig. S7). In each read-depth histogram, we chose three cut-offs to capture two peaks of the bimodal distribution that correspond to haploid and diploid levels of coverage. The

first read-depth peak resulted from the duplicated regions and corresponds to the 'haploid' level of coverage. The second read-depth peak resulted from regions that are haplotype-fused and corresponded to the 'diploid' level of coverage. We removed everything that was not classified as primary and repeated contigs from the assembly. We additionally generated an assembly where repeat contigs were removed. The number of contigs went down to 1,254 with repeats and 570 without repeats. The rate of duplicated genes was 12.8% after filtration (see Tables S1 and S2). We kept contigs containing repeats in the final assembly because they may represent regions of interest for further research (e.g., transposable elements, etc.).

Filtering for contaminants

We screened the barnacle assembly for contamination since the genomic DNA samples came from wild barnacle specimens that may have other species in and on them. The contamination search was first attempted using Kraken 2 [46] [47] against the complete Kraken database. Since the Kraken database does not include a nearby reference genome for barnacles, Kraken 2 unclassified the majority of scaffolds and, more problematically, classified them into unrelated taxa including vertebrates, fungi, and plants. Most of these classifications were unlikely (e.g., vertebrates and terrestrial plants present on barnacles), especially given that these results were not confirmed later by manual BLAST searches. This issue was likely caused by the absence of a close reference, and, as a result, each scaffold was classified with minor identity by the k-mer approach used in Kraken.

Therefore, we used a modified version of a method for removing human DNA contamination in bacterial genome assemblies recently proposed [48]. Briefly, the original method divides genome scaffolds into overlapping subreads and maps each subread to the reference database using NCBI BLAST [49]. We partitioned our barnacle reads into 10 Kbp pseudoreads with 5 Kbp overlap. Pseudoreads were then aligned to the NCBI nucleotide database (https://www.ncbi.nlm.nih.gov/nuccore) using MegaBLAST [50] with custom parameters. In contrast to the original strategy, where the authors used NCBI RefSeq database [51], we mapped against the NCBI nucleotide

database since there are not many reference assemblies for crustaceans and we wanted all accessions to the nearest sequenced organisms.

We performed two levels of analysis. First, we analyzed hits with an arbitrary length of alignment and e-value <0.01. Second, we analyzed hits with length of alignment ≥500 bp (minimal length of the PacBio error-corrected reads) and e-value <1e-50. The former method showed more homologous hits while the latter method showed more hits with contamination. Plots with the color representation of each scaffold subreads taxonomy classification were built and contaminated scaffolds were identified. For each pseudoscaffold classified as a contaminant, the full scaffold from which it came was then aligned to the NCBI nucleotide database with MegaBLAST to confirm bacterial contamination and when confirmed, the contaminated scaffolds were removed from the assembly.

Polishing

The filtered assembly of long reads from PacBio reads are prone to insertion and deletion errors, which usually are corrected by polishing. Our assembly was polished using Illumina WGS reads. Three rounds of polishing were completed using Pilon (v1.23) (-fix-all) to produce the final assembly. Pilon confirmed 83.9% of the assembly with Illumina reads alignment at the first round and 85.35% at the third round. Supplemental Table S3 provides detailed statistics of individual base, indels, and gap corrections for each round. After three rounds, the percentage of complete Arthropoda BUSCO genes equaled 90.5%.

Genome Size Estimation

Pollicipes pollicipes genome size was estimated from the final assembly length and by k-mer analysis (k=21) of the Illumina gDNA pair-end reads for validation. The frequency distribution of 21-mers was computed by Jellyfish [52] v2.3.0 [53]. Figure S3 shows bimodal frequency distribution of 21-mers. The first and second peaks in the distribution correspond to 21-mers from heterozygous and homozygous regions, respectively. The average 21-mer coverage was 62 for heterozygous regions and 124 for homozygous ones. We approximated the frequency distribution with two normal

distributions with means 62 and 124 and standard deviations 14 and 20, respectively, to estimate the monoploid genome size.

Genome Annotation

Genome annotation was performed with the NCBI Eukaryotic Genome Annotation Pipeline [54] v8.5. Briefly, masking of repeats was first attempted with RepeatMasker, but due to the lack of a comprehensive repeat library, repeats were masked with WindowMasker [55]. Available transcripts, RNA-Seq (Table S4), and protein data from RefSeq [56] were aligned to the masked genome using BLAST followed by refinement with SPLIGN [57]. Protein, transcript, and RNA-Seq alignments were used as input for two rounds of gene prediction with Gnomon [58] (https://www.ncbi.nlm.nih.gov/core/assets/genome/files/Gnomon-description.pdf). The final set of annotated features was built by evaluating the known RefSeq transcripts, the features projected from curated RefSeq genomic alignments, and the most highly-supported models predicted by Gnomon, respectively, at each locus. Protein naming, determination of locus type, and GeneID assignment followed the NCBI Eukaryotic Genome Annotation Pipeline standards.

Transcriptome assembly

All transcriptomes were assembled de novo as follows. Raw reads were downloaded from NCBI SRA [59], read quality was assessed using FastQC [60] v0.11.8 [61], reads were subjected to quality and adapter trimming using Trimmomatic [62] v0.33 (ILLUMINACLIP: TruSeq3-PE-2.fa:2:30:10 LEADING:3 TRAILING:3 SLIDINGWINDOW:4:15 MINLEN:50) [63], and quality trimming and adapter removal was confirmed using FastQC again after trimming. Trimmed reads were error-corrected using Rcorrector [64] v1.0.4 [65] with default settings. Error corrected reads were assembled using Trinity [66] v2.10.0 [67,68] under default parameters except that minimum kmer coverage was set to 2. Assembled contigs were translated to amino acid (AA) sequences using TransDecoder [69] v5.2.0 [67] with open reading frames identified using default parameters.

Table 3. Comparison of chromosome-level crustacean genome assemblies and other barnacle genomes

Contiguity	Taxon	Species	Assembly	Genome size	Scaffold N50	Scaffold L50	Arthropod BUSCO	Reference
**Chromosome**	**Thecostraca**	***Pollicipes pollicipes***	**GCA_011947565.2**	**770 MB**	**47,009,503**	**8**	**90.5%**	**this study**
Scaffold	Thecostraca	*Amphibalanus amphitrite*	GCA_009805615.1	613 MB	458,238	415	92.4%	[70]
Scaffold	Thecostraca	*Semibalanus balanoides*	GCA_014673585.1	482 MB*	56,726	1,896	56.4%	NCBI
Contig	Thecostraca	*Semibalanus balanoides*	GCA_003709985.1	101 MB*	1,475	24,797	14.5%	NCBI
Chromosome	Branchiopoda	*Daphnia carinata*	GCA_013167095.1	132 MB	8,418,570	7	98.6%	NCBI
Chromosome	Branchiopoda	*Daphnia magna*	GCA_003990815.1	123 MB	10,124,675	6	98.0%	[71]
Chromosome	Copepoda	*Caligus rogercresseyi*	GCA_013387185.1	478 MB	27,802,916	8	61.5%	NCBI
Chromosome	Copepoda	*Tigriopus californicus*	GCA_007210705.1	191 MB	15,806,032	6	93.5%	[72]
Chromosome	Copepoda	*Tigriopus japonicus*	GCA_010645155.1	197 MB	10,654,335	8	94.1%	[73]
Chromosome	Decapoda	*Eriocheir sinensis*	GCA_013436485.1	1272 MB	17,608,299	30	92.6%	[74]
Chromosome	Decapoda	*Paralithodes platypus*	GCA_013283005.1	4805 MB	51,153,954	39	81.4%	[75]

Entries in gray indicate other barnacle assemblies

* The size of this assembly is much shorter than the estimated size of the haploid genome (1,300–1,600 MB)

Ortholog identification

Orthologs were identified using a phylogenetic approach following Yang and Smith [76] (scripts from https://bitbucket.org/yangya/phylogenomic_dataset_construction/src/master). First, the predicted proteins from the transcriptomes had redundancy in AA sequence reduced using CD-HIT [77] v4.6.8 [78,79] with a 99% similarity threshold. Then the transcriptomes and genome were subjected to an all-by-all BLAST search (-max_target_seqs 1000 -evalue 10) and the resulting BLAST output was filtered for a hit fraction ≥0.4. Filtered BLAST hits were further clustered using MCL [80] v14.137 [81] with a -log E-value cutoff set to 5 and an I-value of 1.4 to identify homologous protein sequences. Fasta files were written from the MCL output using write_fasta_files_from_mcl.py.

Each cluster of homologs was then aligned individually with MAFFT [82] v7.427 (–genafpair–maxiterate 1000 if <1,000 sequences; –auto if >1,000 sequences) [83], trimmed using phyutility (minimum column occupancy = 0.1) [84], and trees were built using either RAxML [85] v8.2.12 [86] under the model "PROTGAMMALG" for clusters with less than 1,000 sequences, or FastTree [87] v2.1.10 [88] under the model "-lg" for clusters greater than 1,000 sequences. The resulting trees may contain branches representing paralogs or misassembled contigs, so they were filtered using the following three methods from Yang and Smith [76]. First, divergent sequences were removed

Table 4. Taxa and orthologs used in phylogenetic analyses

Taxon	# Orthologs (%)	AA positions	Accession numbers
Pollicipes polymerus	2137 (37%)	314,239	SRR10034703
Capitulum mitella	2220 (39%)	475,439	SRR10012027
Loxathylacus texanus	2216 (39%)	534,367	SRR5140130
Semibalanus balanoides	3685 (64%)	678,927	SRR5140144
Sacculina yatsui	2238 (39%)	783,829	DRR169034, DRR169035, DRR169036
Balanus improvisus	4232 (74%)	931,663	SRR8775110
Tetraclita japonica	4571 (80%)	1,137,491	SRR426837
Chthmalus fragilis	4833 (84%)	1,188,730	SRR4113502
Lepas anatifera	4919 (86%)	1,653,588	SRR6818896
Pollicipes pollicipes	5092 (89%)	1,742,039	GCA_011947565.2
Megabalanus volcano	5363 (94%)	1,777,080	SRR5091879, SRR5091880
Octolasmis warwickii	5161 (90%)	1,790,600	SRR10527303
Glyptelasma gigas	5221 (91%)	1,790,661	SRR10523768
Amphibalanus amphitrite	5385 (94%)	1,807,445	SRR10034703

from clusters if a terminal branch was longer than 0.75 or more than 10x longer than its sister using trim_tips.py, following the parameters used for the MIL dataset, a taxon of similar age, in Yang and Smith [76]. Next, if monophyletic or paraphyletic tips from the same taxa were present in a tree, only the sequence with the highest number of non-

ambiguous characters in the trimmed alignment was kept and the rest removed following previously published methods [76,89]. Lastly, potential deep paralogs were removed using cut_long_internal_branches.py with an internal branch length cutoff of 1.5 and a minimum number of taxa of 7 (i.e., 50%). Fasta files were written from the trimmed trees and alignments and the entire process of aligning, trimming alignments, building trees, and removing paralogs and long branches was repeated. After the second round of refinement, the trees were called homolog trees and were further pruned to call orthologs.

Orthologs were called using the maximum inclusion method [76,89,90]. After pruning the homolog trees to identify maximum inclusion orthologs, the remaining subtrees might still have contained terminal taxa subtended by long branches as a result of the subtree trimming method [76]. To account for this, the trees were trimmed once more using a range of permissive-to-strict branch length trimming parameters, referred to from here on as permissive and strict branch trimming, with relative branch lengths of 10x and absolute branch lengths of 0.4 or 0.3 at the permissive and strict levels, respectively. Because of the large number of orthologs retrieved from both trimming parameters, the orthologs resulting from the strict, more conservative, trimming were used for downstream phylogenetic analyses.

Phylogenomics

The final orthologs were aligned individually using MAFFT and trimmed using Gblocks [91] v0.91b [92] following the same parameters detailed above. The Gblocks trimmed alignments were then concatenated using concatenate_matrices.py with a minimum length of 100 AA and a minimum taxon cutoff of 7 (50%). Phylogenetic analyses were completed using concatenation and coalescent methods. Concatenated analyses were done with a maximum likelihood (ML) partitioned analysis and with a ML mixture model. The partitioned, concatenated analysis was carried out using IQTree [93] v1.6.11 [94]. Partitions and models of evolution for each partition were selected using the fast relaxed-hierarchical clustering algorithm (-rclusterf) [95], followed by tree building with 1,000 ultrafast bootstrap pseudoreplicates [96]. Mixture models were also used for ML tree search because they account for among-site-variation in AA

propensities, and thus are less prone to artifacts like long branch attraction [97–99]. For the ML mixture model tree search, the c20 mixture model implemented in IQTREE v1.6.11 (-m LG+C20+F+G) was used to build a starting tree, and the resulting tree was used as a guide tree for a c60 posterior mean site frequency model (PMSF) [100] (-m LG+C60+F+G -ft) with 100 bootstrap pseudoreplicates. For the coalescent approach, individual gene trees were built for each ortholog using IQTree and the substitution model of best fit (-mfp) with 1,000 rapid bootstrap pseudoreplicates. A species tree was then estimated by using all gene trees as input in ASTRAL [101] v5.6.3 [102].

Differential gene expression

Raw RNA-Seq reads for 2 replicates each of *P. pollicipes* nauplii and adults were downloaded from NCBI (Table S4) [103]. Reads were subjected to error correction with Rcorrector [65] using default settings and aligned to the *P. pollicipes* genome assembly GCA_011947565.2 downloaded from NCBI using HISAT2 [104] v2.1 [105]. A GTF file was generated using GffRead [106] v0.12.7 [107] and the *P. pollicipes* genome GFF file from NCBI. Read counts were generated using featureCounts in the Subread package [108] v2.0.1 (-t exon -g gene_name) [109]. Differential gene expression analysis was performed using DESeq2 [110] v1.34 [111] with default settings. Results were considered significant when $P < 0.05$ after FDR correction ($q < 0.05$). A $\log_2$ fold-change of at least 2 was used to further filter differentially expressed genes. To estimate the number of genes with expression unique to, or shared between, the nauplius and adult stages, FPKM was calculated for each sample using DESeq2. Genes with FPKM <0.5 were counted as not expressed due to the presence of transcriptional noise in RNA-Seq datasets [112,113].

To classify the differentially expressed genes (DEGs) into functional categories, the AA sequences of all genes were mapped to GO terms [114,115] by identifying pfam domains [116] with InterProScan [117] 5.46-81.0 [118]. Because DESeq2 maps reads to genes and not constituent isoforms, when more than one isoform was present for a gene, the longest isoform was used for the functional mapping of DEGs. Enrichment analyses of GO terms was carried out with topGO [119] v2.44 [120] (nodeSize = 5) by comparing GO terms from DEGs to GO terms of all expressed protein coding genes in

the genome, and significance was determined using Fisher's exact test ($q < 0.05$). To further identify functional categories and pathways, DEGs were mapped to KEGG orthologs and pathways [121] using KofamKOALA [122] v2021-10-03 with an E-value cutoff of 0.01 [123].

Results

Larval development and life cycle

Here we document the life cycle of *P. pollicipes.* It is not the purpose of this study to describe all morphological changes seen during larval development, nor was it our intention to report on the setation formulae of naupliar stages. Briefly, the first nauplius stage (N1) hatches from the egg and is nonfeeding (lecithotrophic), relying on yolk stores (Fig. 1A). This stage is morphologically reduced compared to the later, more complex naupliar instars (Fig 1A-B). Shortly after hatching, the nauplius undergoes a series of five molts of feeding instars, finally arriving at the N6 (Fig. 1B). The nauplius increases in size by nearly 3x during development, with the N1 growing from 0.21-0.24 mm to a N6 0.56-0.62mm (N=50 per stage). After 9-11 days post-hatching, the N6 molts into the next larval phase, the cyprid, which is a stage specialized for settlement (Fig. 1C). Using modified antennules equipped with a battery of sensory and attachment structures (Fig. 1C), the cyprid walks over substrates in a bipedal, exploratory manner. After 2-3 days of various probing behaviors, the cyprid commits to permanent settlement by releasing larval cement. Juvenile metamorphosis begins followed by subsequent molting into the adult phase (Fig. 1D). Juveniles and adults grow rapidly from a roughly 0.5 mm cyprid to large stalked adults that can exceed 3 cm in length and shell plate widths over 1 cm. Adults are often found in clusters of individuals (Fig. 1E). The life cycle is completed when hermaphroditic adults cross fertilize via an extensible penis (Fig. 1E) and produce clutches of hundreds of eggs.

Genome assembly

We sequenced the *P. pollicipes* genome with 62x PacBio coverage, 196x Illumina WGS coverage, 203x HiC coverage, and 69x Capture Hi-C (CHi-C) coverage (Table 1). The total assembly length was 770 Mbp with a scaffold N50 of 47 Mbp, a

scaffold L50 of 8, and the largest scaffold being 64 Mbp (Table 2). More than 92% of the assembly length was composed of 17 large scaffolds (Fig. S5, S6). Of the 1,066 genes in the arthropod BUSCO gene set [124,125], 90.5% of them were assembled completely, 3.3% were fragmented, and 6.2% were missing from the assembly. Results from BUSCO analysis of the 978 conserved singly-copy metazoan genes were similar, with 91.2% were assembled completely, 4.4% fragmented, and 4.4% missing (Table 2). Using a modified version of genome contamination removal suggested in [48], we identified 62 of the 576 scaffolds in the assembly as bacterial contaminants, which we then removed. All 62 contaminant scaffolds were relatively small and collectively comprised about 7 Mbp, or just <1% of the final assembly length. This method is highly effective at identifying sequences containing homogenous contaminant DNA, but it may be less effective in the presence of a small proportion of chimeric contamination. We also assembled the mitochondrial genome separately using the PacBio reads and Canu, and then polished the assembly with 6,000x Illumina coverage using Pilon. The final mitochondrial genome was 15,090 bp.

To validate the genome size measured from the assembly length, we also estimated the genome with a kmer analysis of Illumina WGS using Jellyfish. The estimated genome size from Jellyfish was 702 Mbp, close to the total length of the resulting assembly (770 Mbp), indicating our assembly covers the majority of the genome well. However, there was a double peak in the distribution of k-mers in the Jellyfish estimate (Figure S7), which impacts k-mer based size estimates [126]. This bimodal distribution is typical of heterozygous genomes [127], which is unsurprising given the samples we sequenced were of non-inbred individuals from a large, wild population. As a result, the assembly length of 770 Mpb was used to calculate coverage estimates.

We compared our newly generated *P. pollicipes* assembly with the seven other available chromosome-level crustacean assemblies (*Caligus rogercresseyi, Daphnia carinata, D. magna, Eriocheir sinensis, Paralithodes platypus, Tigriopus californicus, T. japonicus*) and the three other available barnacle genome assemblies (Table 3). Since BUSCO scores and contiguity statistics were not provided for all of these assemblies,

we generated BUSCO reports and measured N50 and L50 for each for comparative purposes.

Genome annotation with the NCBI Eukaryotic Genome Annotation Pipeline identified 31,804 transcripts and 25,694 genes. Of the genes, 20,444 were protein coding, 4,220 were noncoding, 1,030 were pseudogenes. The 24,664 genes (excluding pseudogenes) had a mean length of 13,244 bp and a median length of 6,980 bp. An average of 1.3 transcripts were identified for each gene with an average of 7.48 exons per transcript. Exons had a mean length of 241 bp while introns averaged 2,077 bp. RepeatMasker identified 3.2% of the genome as repetitive, but a comprehensive repeat library is not available for barnacles, especially not for gooseneck barnacles, and nearly all repeats were classified as simple repeats or low complexity repeats. To avoid reliance on a repeat library, WindowMasker was used and masked 18.5% of the genome prior to annotation.

Phylogenomics

A phylogenetic analysis of selected barnacles was performed using the *P. pollicipes* genome and transcriptomes from 13 other barnacle species (Table 4). In total, 5,734 orthologs of at least 100 AA were identified, which produced a concatenated alignment 1,999,119 AA long in length. The Rhizocephala was selected as the outgroup following previous studies [5,7,128,129]. All concatenated and coalescent-based phylogenetic analyses had identical topologies, and each tree had maximum support values (PP=1, BS = 100%) for all nodes. The tree from the partitioned ML analysis is shown in Figure 3B.

Differential gene expression of nauplii and adults

On average, 76.4% of RNA-Seq reads per sample aligned to the *Pollicipes* genome. The total aligned reads per sample are as follows: larva1 = 26.6 million, larva2 = 12.5 million, adult1 = 34.2 million, adult2 = 26.1 million. In total, reads aligned to 23,075 genes from the assembly. After removing genes with very low expression (< 0.5 FPKM) to filter out transcriptional noise, we observed 2,083 genes expressed only in the nauplius stage, 2,337 unique to the adult stage, and 13,352 genes were expressed in

both stages (Fig. 3A). However, many of the shared genes differed in their expression level. Of the 24,668 genes in the *P. pollicipes* genome, 11,846 were DEGs between the nauplius and adult stages after FDR correction (q value < 0.05). A similar proportion of the DEGs were overexpressed in each stage (5,870 in nauplii, 5,976 in adults). To further filter the DEGs, a log_2 fold-change > 2 cutoff was applied, which resulted in 5,198 DEGs (2,400 overexpressed in nauplii, 2,789 overexpressed in adults). Of these DEGs, 91 and 112 in nauplii and adults, respectively, were classified as pseudogenes in the genome annotation, while 332 genes in nauplii and 148 genes in adults were long non-coding RNAs (lncRNAs).

To explore the functions of DEGs, they were further mapped to GO terms and KEGG orthologs and pathways. We attempted to map all expressed protein coding genes to GO terms with pfam and annotated 51% (10,436/20,443) of all genes with GO terms, including 51% (2,321/4,507) of the most highly DEGs. Figure 3B shows the most significant, enriched GO terms in the nauplius stage accounting for the nesting of GO terms (see full results in Table S5, Figs. S9–S11). DEGs in nauplii were enriched for molecular motor activity, peptidases, homophilic cell adhesion via membrane-bound proteins, and chitin binding proteins, among others (Fig. 3B). Overall, secretory proteins were enriched in nauplii relative to adults. Results of the GO enrichment analysis in the adult stage are likewise provided (Fig. 3C, Table S6, Figs. S12–S14). DEGs in adults were enriched for structural components of cuticle, iron/heme binding, oxioreductase and hydrolase activity, sodium and anion transport, and chitin binding (Fig. 3D). Enrichment for membrane bound proteins was highly significant (p = 5.6E-10) in adults but not in nauplii.

Functions of DEGs were also examined using KEGG orthologs and KEGG pathways. Of the protein coding DEGs, 82% (3,685/4,507) were assigned to KEGG orthologs using KofamKOALA and these mapped to 335 KEGG pathways (Tables S7, S8). The most frequently identified KEGG pathways assigned to DEGs overexpressed in the nauplius with the percent of annotated DEGs follow by the count of DEGs in parentheses were represented as follows: metabolism (6.6%, 104), biosynthesis of secondary metabolites (4.8%, 76), transport and catabolism (2.8%, 45), signal transduction (2.4%, 38), carbohydrate metabolism (2.3%, 36), glycan biosynthesis and

metabolism (2.1%, 33), amino acid metabolism (1.6%, 25), and transcription and translation (1.5%, 23). The most frequently identified KEGG pathways assigned to DEGs in adults were: metabolism (11%, 229), carbohydrate metabolism (3.7%, 78), signal transduction (3.7%, 78), biosynthesis of secondary metabolites (3.6%, 76), amino acid metabolism (2.8%, 59), biosynthesis of cofactors (2.6%, 54), metabolism of cofactors and vitamins (2.3%, 49), and lipid metabolism (2.2%, 46).

The functional annotation from the NCBI annotation pipeline was examined manually for the top 100 most differentially expressed genes ($q < 1E-10$, $\log_2$ fold-change > 7) (Fig. 3D). The most common annotations for these DEGs were as follows: 18 were cuticle proteins (all upregulated in the nauplius); 14 were various enzymes (e.g., proteases, deacetylases, oxygenases, one RNA helicase); 4 were heat shock proteins, 4 were involved with chitin modifications (deactylase, chitinase, prisilkin-39-like, peritrophin-1-like), 4 were pseudogenes; 3 were involved with vision pathways (2 calphotins, 1 opsin, all upregulated in the nauplius); 3 were lncRNAs; and the remainder had miscellaneous functions.

Discussion

Genome Assembly

We assembled a highly contiguous genome for the stalked barnacle *P. pollicipes.* More than 92% of the assembly length was composed of 17 large scaffolds, which likely represent 16 or 17 chromosomes or chromosome arms (Fig. S5, S6). The smaller 17th scaffold may represent a small chromosome or the arm of a chromosome that remained unlinked. It is difficult to confirm a chromosome count for *P. pollicipes* because the number of chromosomes has not yet been recorded for this species, and chromosomal counts in crustaceans are highly variable [132]. Nonetheless, the scaffolds assembled here are as long or longer than most chromosome-level assemblies in other crustaceans (Table 3). Our assembly has greater contiguity than all other chromosome-level crustacean assemblies, except for the relatively giant genome of the blue king crab *Paralithodes platypus*. Moreover, our barnacle assembly has relatively high BUSCO scores. Notably, this assembly has higher contiguity and BUSCO scores compared to all other barnacle genome assemblies (Table 3).

Phylogenomics

The Pollicipedomorpha has proven to be one of the most difficult clades of stalked and acorn barnacles to resolve in phylogenetic analyses, both in terms of its relationship to the other barnacle orders and also the relationships among its four genera [7]. Barnacle phylogenies based on morphology or molecular data have yielded very different results depending on the characters or genes used (e.g., Fig. 2A). Historically, larval characters have played a major role in our understanding of the phylogeny and evolution of thecostacans, which is especially true for the parasitic taxa that often lack traditional adult barnacles characters [5,7]. Still, using a matrix of 41 larval characters across all major barnacle lineages, Pérez-Losada et al. [128] were not able to resolve thecostracan relationships below the sub-class level. Similar attempts to code larval characters for phylogenetic inference [e.g., 133,134] ultimately failed at recovering topologies consistent with those inferred using nuclear and mitochondrial protein coding and ribosomal genes (Fig 2A). While adult characters consistently unite *Capitulum* and *Pollicipes,* larval characters have separated the genera in some analyses. Further obscuring this situation, phylogenetic analyses of characters from different larval stages have led to conflicting phylogenies. For example, because *Pollicipes* and balanomorphans share some naupliar characters (e.g., more oval carapaces and lack of marginal carapace spines; Fig. 1A, B; [135–137]) that are absent in *Capitulum,* Korn [133], Newman and Ross [134] found *Pollicipes* nested within Balanomorpha and Korn [133] found *Capitulum* within Scallpelomorpha. Cypris larval characters, however, united *Capitulum* and *Pollicipes* (i.e., heavily ornamented carapaces and third antennal segments surrounded by a series of velar flaps or filaments [138]).

Here, we resolved part of the Pollicipedormopha conundrum with phylogenomic analyses of nearly 2 million AA positions from 14 barnacles. We found robust support for the independence of the order and its sister relationship with the Balanomorpha. Reinterpreting the larval characters in light of this phylogeny suggests that the shared naupliar features in *Pollicipes* and balamorphan taxa that are lacking in *Capitulum* are the result of homoplasy. Still, questions remain regarding the interrelationships of the 4

pollicipedomorphan genera. To further resolve the situation, *Analesma* and *Lithotrya* must be included in future phylogenomic analyses. Sampling the 8 remaining species in the Pollicipedomorpha is thus within reach, and is crucial to understanding the evolution of key larval characters in this morphologically diverse order. Taken together, this work supports the validity of the Pollicipedomorpha and highlights the fact that larval character analyses should be coupled with robust molecular phylogenetic hypotheses to understand barnacle evolution.

Differential gene expression of nauplii and adults

The differences in the transcriptomes of the nauplius and adult *P. pollicipes* are striking. Nearly half of all genes (i.e., 11,846/24,664) undergo significant differential expression between these stages. These transcriptional differences reflect the vastly different biology of larval and adult barnacles. For example, among the 100 most differentially expressed genes, cuticle proteins were highly upregulated in the nauplius, a stage in which cuticle is rapidly being modified as individuals molt 6 times within 10–25 days [139] (Fig 1A–C). Similarly, genes related to vision were upregulated in nauplii, which is unsurprising considering that nauplii possess an eye whereas adults do not. Numerous structural proteins (e.g., chitin, keratin, collagen) were upregulated in adults; at this stage, barnacles grow orders of magnitude larger and build shell plates and a peduncle >2x their body length (Fig. 3D). One of the most upregulated genes in adults was vitellogenin-1-like (Fig. 3D), which is in alignment with the developmental biology of *Pollicipes*; adults provide large yolk stores that the non-feeding (lecithotrophic) first nauplius stage relies on. Adults DEGs were also enriched for heme proteins (Fig. 3C), which may be necessary for oxygen delivery given that, at their size, adult gooseneck barnacles cannot rely on passive diffusion of oxygen like nauplii.

GO enrichment analysis and KEGG pathways also revealed broader patterns. Nauplii upregulated genes involved in tissue morphogenesis, such as homophilic cell adhesion genes including cadherins (Fig. 3B, Table S5) [140], while adults upregulated proteins that may be involved in tissue modeling and adhesion, such as cuticle-binding proteins, chitin binding proteins, and collagen trimers (Fig. 3C, Table S6). At the broadest levels, the enrichment analysis showed nauplii upregulate excretory (i.e.,

secretory) proteins, while adult barnacles upregulated more membrane bound proteins (Fig. 3B, C, Table S5, S6). Furthermore, preliminary pathway analysis showed that adults upregulate genes involved in carbohydrate metabolism, lipid metabolism, metabolism of cofactors and vitamins, and amino acid metabolism. One likely factor at play here is dietary change; nauplii feed on small single-celled phytoplankton, while adults feed mostly on small crustaceans [139,141]; concordantly, DEGs were often involved in macronutrient metabolism in the KEGG pathway and GO enrichment analyses. A total of 332 lncRNAs were also differentially expressed between adults and nauplii (Fig. 3D). Given that lncRNAs are thought to play important roles in gene regulation [142], further research is needed to assess their associations to mRNAs.

Overall, the differences in larval and adult transcriptomes of *P. pollicipes* are substantial. This is true even when compared to transcriptional differences typically seen in other arthropods with profound metamorphoses, such as holometabolous insects. For example, in *Anopheles* [143], *Apis* [144], *Drosophila [145]* and other insects [146–148], typically around 3–30% of their genes are differentially expressed between larva and adult stages compared to the 48% of genes observed here in *P. pollicipes.* Stark differences in the biology of the nauplius and adult barnacle stages appear to be reflected in drastic transcriptomic differences both in the degree of expression of nearly ½ of their genes as well as the number of genes (>4,000) that exhibited stage-specific expression (Fig. 3A). A noteworthy limitation of this DEG analysis is that there were only 2 replicates each for the nauplius and adult stages, though the nauplius libraries were prepared from pooled individuals [74], which reduces sample variability and helps compensate for the lack of replication [149]. Still, a better understanding of transcriptional differences across *P. pollicipes* life stages requires additional replication, ideally with nauplius samples separated by stage (i.e., N1–N6), and samples of the cyprid stage (Fig. 1).

Conclusions

By combining Illumina short reads, PacBio long reads, and Hi-C and CHi-C chromatin-conformation capture data, we produced a high-quality genome assembly and annotation for the gooseneck barnacle *P. pollicipes*. This is one of the most

contiguous crustacean genomes to date and the most complete assembly for a barnacle species. Using the genome annotation and transcriptomic data from 13 other barnacles, we completed phylogenetic analyses with the greatest number of orthologs and AA positions to date for barnacles and showed that the Pollicipedomorpha is a monophyletic order sister to Balanomorpha (Fig. 2). Our differential gene expression analysis of nauplii and adult transcriptomes revealed large differences in metabolic function and regulation in *P. pollicipes*, underlying the vast difference in lifestyle between these two stages. This study hence provides a valuable example of good genomic practices, high quality genomic resources for a key group of crustaceans, and valuable insights into the evolution and development of barnacles.

Acknowledgments

We thank the Earth BioGenome Project for helpful discussions on genome assembly, annotation, and analysis approaches. We are grateful to Dr. Jens Høeg (University of Copenhagen), Dr. Hamad Al-Yahya (King Saud University), and Mr. Stefan Sørensen (University of Copenhagen) for providing larval and adult specimens. We thank Dr. Perina Cedrón and the authors of Machado et al. [150] for making *P. pollicipes* RNA-Seq data publicly available for study.

Data Availability

BioProject: PRJNA614970 (*Pollicipes pollicipes*)

Associated BioProjects: PRJNA533106 (EBP); PRJNA649812 (GIGA)

WGS accession number: SUB8253844

Mitochondrial genome accession: CM029732

Biosample: SAMN14444043

WGS Project: JAAVLY02

GenBank (and RefSeq) assembly accession: GCA_011947565.2

NCBI Annotation (Release 100):

https://www.ncbi.nlm.nih.gov/genome/annotation_euk/Pollicipes_pollicipes/100/

SRA accessions: SRR11456527-40 (PacBio), SRR12730898 (Illumina NextSeq WGS), SRR11483033-34 (HiC), SRR11483035-36 (CHi-C)

TSA accession: SUB10638669

Hologenophore: USNM 1622609

Paragenophores: USNM 1622610 (lot of 6 specimens)

Author Contributions

KAC and MPL conceived the project and funded the effort. NA secured funding for AZ. MPL collected the specimens for the study (at great personal peril). JPB, PA, AZ conducted data cleaning including contaminant filtering, alternative assembly approaches, and bioinformatic analyses. JPB deposited raw sequence data, assemblies, and specimen information to NCBI. KAC deposited specimens into the US Museum of Natural History Crustacean Collection at the Smithsonian Institution. NCBI conducted the annotation. ND performed scanning electron- and light microscopy of larval and adult specimens and assisted with the phylogenomic analyses. NA and PA supervised AZ. KAC, JPB, PA, ND, and AZ wrote the original draft of the manuscript and all authors read and approved the manuscript.

Funding

This work was supported by the Computational Biology Institute at George Washington University and an award from Dovetail Genomics. The work of AZ is supported by JetBrains Research. The work of NA is supported by the Government of the Russian Federation through the ITMO Fellowship and Professorship Program. JPB was supported in part by the NSF Postdoctoral Research Fellowships in Biology Program under Grant No. 2010898. ND was sponsored by a double-degree graduate stipend by Biodiversity Research Center, Academia Sinica (NTNU), and Natural History Museum of Denmark (University of Copenhagen). Any opinions, findings, and conclusions or recommendations expressed in this material are those of the author(s) and do not necessarily reflect the views of the National Science Foundation.

US National Science Foundation, Postdoctoral Research Fellowships in Biology, 2010898, JP Bernot;

George Washington University, Computational Biology Institute, M Pérez-Losada, K Crandall;
Dovetail Genomics, 2017 Matching Funds Award, 00001174, K Crandall;
JetBrains Research, A Zamyatin;
Government of the Russian Federation, ITMO Fellowship and Professorship Program, N Alexeev;
Biodiversity Research Center, Academia Sinica (NTNU), N Dreyer;
Natural History Museum of Denmark (University of Copenhagen), N Dreyer.

Declaration of Interests

The authors declare they have no competing interests.

Figure Legends

Figure 1. *Pollicipes pollicipes* life cycle. Note the fundamental structural differences among the life history stages. (A) Nauplius stage 1. (B) Nauplius stage 6. (C) Cyprid, insert showing magnified view of the third antennal segment used for permanent attachment to the substratum surface. (D) Juvenile adult. (E) Mature adult, insert showing a dissected adult specimen with six cirri or 'feeding legs', the penis and the gut. (F) *Pollicipes pollicipes* voucher and genome sequencing specimens. Asterisk indicates genome hologenophore specimen (USNM 1622609).

Figure 2. (A) Previous phylogenetic hypotheses on the position of Pollicipedomorpha taxa. (B) Phylogeny of the Cirripedia based on 5,734 protein coding orthologs comprising 1,999,119 AA positions. The topology was identical across all analyses and all nodes received maximum support in all analyses (PP=1, BS = 100%). Branch lengths for the partitioned ML analysis are shown. Illustrations from Darwin [8,130] except for Rhizocephala, which is from Haeckel [131].

Figure 3. (A) Venn diagram of genes expressed in each life stage. (B) Summary of most significant, enriched GO terms in nauplius DEGs accounting for nested GO terms. (C) Summary of most significant, enriched GO terms in adult DEGs accounting for nested GO terms. (D) Heatmap of top 100 differentially expressed genes including gene IDs and annotations, clustered according to expression on the y-axis. N 1 = nauplius 1, N 2 = nauplius 2.

References

1. Lewin HA, Robinson GE, Kress WJ, Baker WJ, Coddington J, Crandall KA, et al.. Earth BioGenome Project: Sequencing life for the future of life. *Proc Natl Acad Sci U S A*. 2018; doi: 10.1073/pnas.1720115115.

2. Community of Scientists G. The Global Invertebrate Genomics Alliance (GIGA): Developing Community Resources to Study Diverse Invertebrate Genomes. *J Hered*. Oxford Academic; 2014; doi: 10.1093/jhered/est084.

3. Martin JW, Davis GE. An updated classification of the recent Crustacea. Natural History Museum of Los Angeles County Los Angeles;

4. Kolbasov GA. Acrothoracica, burrowing crustaceans. KMK Scientific Press Ltd., Moscow;

5. Høeg JT, Noever C, Rees DA, Crandall KA, Glenner H. A new molecular phylogeny-based taxonomy of parasitic barnacles (Crustacea: Cirripedia: Rhizocephala). *Zool J Linn Soc*. Oxford Academic; 2020; doi: 10.1093/zoolinnean/zlz140.

6. Grygier MJ. Classe des Thecostrace's (Thecostraca Gruvel, 1905): Sous-classe des Facetotecta (Facetotecta Grygier, 1985). *Traite de Zoologie: Anatomie, Systeatique, Biologie, Tome VII*. Masson; :425–321996;

7. Chan BKK, Dreyer N, Gale AS, Glenner H, Ewers-Saucedo C, Pérez-Losada M, et al.. The evolutionary diversity of barnacles, with an updated classification of fossil and living forms. *Zool J Linn Soc*. Oxford Academic; 2021; doi: 10.1093/zoolinnean/zlaa160.

8. Darwin C. A Monograph on the Fossil Balanidae and Verrucidae of Great Britain. Palaeontographical Society;

9. Darwin C. A Monograph on the Fossil Lepadidae, Or, Pedunculated Cirripedes of Great Britain. Paleontographical Society;

10. Love AC. Darwin and Cirripedia prior to 1846: Exploring the origins of the barnacle research. *J Hist Biol*. Springer; 35:251–892002;

11. Deutsch J. Darwin and barnacles. *C R Biol*. 2010; doi: 10.1016/j.crvi.2009.11.009.

12. Schultz MP, Bendick JA, Holm ER, Hertel WM. Economic impact of biofouling on a naval surface ship. *Biofouling*. 2011; doi: 10.1080/08927014.2010.542809.

13. Pérez-Losada M, Høeg JT, Simon-Blecher N, Achituv Y, Jones D, Crandall KA. Molecular phylogeny, systematics and morphological evolution of the acorn barnacles (Thoracica: Sessilia: Balanomorpha). *Mol Phylogenet Evol*. Elsevier; 2014; doi: 10.1016/j.ympev.2014.09.013.

14. Lozano-Fernandez J, Giacomelli M, Fleming JF, Chen A, Vinther J, Thomsen PF, et al.. Pancrustacean Evolution Illuminated by Taxon-Rich Genomic-Scale Data Sets with an Expanded Remipede Sampling. *Genome Biol Evol*. academic.oup.com; 2019; doi: 10.1093/gbe/evz097.

15. Lin H-C, Høeg JT, Yusa Y, Chan BKK. The origins and evolution of dwarf males and habitat use in thoracican barnacles. *Mol Phylogenet Evol*. 2015; doi: 10.1016/j.ympev.2015.04.026.

16. Rees DJ, Noever C, Høeg JT, Ommundsen A, Glenner H. On the origin of a novel parasitic-feeding mode within suspension-feeding barnacles. *Curr Biol*. 2014; doi: 10.1016/j.cub.2014.05.030.

17. Herrera S, Watanabe H, Shank TM. Evolutionary and biogeographical patterns of barnacles from deep-sea hydrothermal vents. *Mol Ecol*. 2015; doi: 10.1111/mec.13054.

18. Rivera A, Gelcich S, García-Florez L, Alcázar JL, Acuña JL. Co-management in Europe: Insights from the gooseneck barnacle fishery in Asturias, Spain. *Mar Policy*. Elsevier; 2014; doi: 10.1016/j.marpol.2014.07.011.

19. Ramos AS, Antunes SC, Gonçalves F, Nunes B. The Gooseneck Barnacle (Pollicipes pollicipes) as a candidate sentinel species for coastal contamination. *Arch Environ Contam Toxicol*. Springer; 2014; doi: 10.1007/s00244-013-9978-1.

20. Rocha M, Antas P, Castro LFC, Campos A, Vasconcelos V, Pereira F, et al.. Comparative Analysis of the Adhesive Proteins of the Adult Stalked Goose Barnacle Pollicipes pollicipes (Cirripedia: Pedunculata). *Mar Biotechnol* . Springer; 2019; doi: 10.1007/s10126-018-9856-y.

21. Webster MS. The Extended Specimen: Emerging Frontiers in Collections-Based Ornithological Research. CRC Press;

22. Lendemer J, Thiers B, Monfils AK, Zaspel J, Ellwood ER, Bentley A, et al.. Corrigendum: The Extended Specimen Network: A Strategy to Enhance US Biodiversity Collections, Promote Research and Education. *Bioscience*. academic.oup.com; 2020; doi: 10.1093/biosci/biz165.

23. Lannom L, Koureas D, Hardisty AR. FAIR Data and Services in Biodiversity Science and Geoscience. *Data Intelligence*. MIT Press; 2020; doi: 10.1162/dint_a_00034.

24. Stall S, Yarmey L, Cutcher-Gershenfeld J, Hanson B, Lehnert K, Nosek B, et al.. Make scientific data FAIR. *Nature*. nature.com; 2019; doi: 10.1038/d41586-019-01720-7.

25. Wilkinson MD, Dumontier M, Aalbersberg IJJ, Appleton G, Axton M, Baak A, et al.. The FAIR Guiding Principles for scientific data management and stewardship. *Sci Data*. nature.com; 2016; doi: 10.1038/sdata.2016.18.

26. De Coster W, D'Hert S, Schultz DT, Cruts M, Van Broeckhoven C. NanoPack: visualizing and processing long-read sequencing data. *Bioinformatics*. academic.oup.com; 2018; doi: 10.1093/bioinformatics/bty149.

27. Putnam NH, O'Connell BL, Stites JC, Rice BJ, Blanchette M, Calef R, et al.. Chromosome-scale shotgun assembly using an in vitro method for long-range linkage. *Genome Res*. Cold Spring Harbor Lab; 26:342–502016;

28. Lieberman-Aiden E, van Berkum NL, Williams L, Imakaev M, Ragoczy T, Telling A, et al.. Comprehensive mapping of long-range interactions reveals folding principles of the human genome. *Science*. science.sciencemag.org; 2009; doi: 10.1126/science.1181369.

29. Callahan BJ, McMurdie PJ, Rosen MJ, Han AW, Johnson AJA, Holmes SP. DADA2: High-resolution sample inference from Illumina amplicon data. *Nat Methods*. nature.com; 2016; doi: 10.1038/nmeth.3869.

30. Kingan SB, Concepcion GT, Dunn C, Pollard M: pb-assembly: PacBio Assembly Tool Suite:

Reads in ⇨ Assembly out. https://github.com/PacificBiosciences/pb-assembly Accessed 2021 Dec 18.

31. Chin C-S, Peluso P, Sedlazeck FJ, Nattestad M, Concepcion GT, Clum A, et al.. Phased diploid genome assembly with single-molecule real-time sequencing. *Nat Methods*. 2016; doi: 10.1038/nmeth.4035.

32. Mikheenko A, Prjibelski A, Saveliev V, Antipov D, Gurevich A. Versatile genome assembly evaluation with QUAST-LG. *Bioinformatics*. 2018; doi: 10.1093/bioinformatics/bty266.

33. Manni M, Berkeley MR, Seppey M, Simão FA, Zdobnov EM. BUSCO Update: Novel and Streamlined Workflows along with Broader and Deeper Phylogenetic Coverage for Scoring of Eukaryotic, Prokaryotic, and Viral Genomes. *Mol Biol Evol*. 2021; doi: 10.1093/molbev/msab199.

34. Stanke M, Keller O, Gunduz I, Hayes A, Waack S, Morgenstern B. AUGUSTUS: ab initio prediction of alternative transcripts. *Nucleic Acids Res*. academic.oup.com; 2006; doi: 10.1093/nar/gkl200.

35. Rhie A, McCarthy SA, Fedrigo O, Damas J, Formenti G, Koren S, et al.. Towards complete and error-free genome assemblies of all vertebrate species. Cold Spring Harbor Laboratory.

36. Lavrov DV, Brown WM, Boore JL. Phylogenetic position of the Pentastomida and (pan) crustacean relationships. *Proceedings of the Royal Society of London Series B: Biological Sciences*. The Royal Society; 271:537–442004;

37. Li Y, Hseih M, Seifert D, Rainish V, Barnett D, Topfer A, et al.: blasr: BLASR: The PacBio® long read aligner. https://github.com/PacificBiosciences/blasr Accessed 2021 Dec 18.

38. Chaisson MJ, Tesler G. Mapping single molecule sequencing reads using basic local alignment with successive refinement (BLASR): application and theory. *BMC Bioinformatics*. Springer; 2012; doi: 10.1186/1471-2105-13-238.

39. Koren S, Walenz BP, Berlin K, Miller JR, Phillippy AM: canu: A single molecule sequence assembler for genomes large and small. https://github.com/marbl/canu Accessed 2021 Dec 18.

40. Walker BJ, Abeel T, Shea T, Priest M, Abouelliel A, Sakthikumar S, et al.: pilon: Pilon is an automated genome assembly improvement and variant detection tool. https://github.com/broadinstitute/pilon Accessed 2021 Dec 18.

41. Walker BJ, Abeel T, Shea T, Priest M, Abouelliel A, Sakthikumar S, et al.. Pilon: an integrated tool for comprehensive microbial variant detection and genome assembly improvement. *PLoS One*. 2014; doi: 10.1371/journal.pone.0112963.

42. Zaharia M, Bolosky WJ, Curtis K, Fox A, Patterson D, Shenker S, et al.: snap: Scalable Nucleotide Alignment Program -- a fast and accurate read aligner for high-throughput sequencing data. https://github.com/amplab/snap Accessed 2021 Dec 18.

43. Zaharia M, Bolosky WJ, Curtis K, Fox A, Patterson D, Shenker S, et al.. Faster and More Accurate Sequence Alignment with SNAP. arXiv [cs.DS].

44. Roach MJ, Schmidt SA, Borneman AR: PurgeHaplotigs. https://bitbucket.org/mroachawri/purge_haplotigs/src/master/ Accessed 2021 Dec 18.

45. Roach MJ, Schmidt SA, Borneman AR. Purge Haplotigs: allelic contig reassignment for third-gen diploid genome assemblies. *BMC Bioinformatics*. 2018; doi: 10.1186/s12859-018-2485-7.

46. Wood D, Lu J, Langmead B: Kraken2. https://ccb.jhu.edu/software/kraken2/ Accessed 2021 Dec 18.

47. Wood DE, Lu J, Langmead B. Improved metagenomic analysis with Kraken 2. *Genome Biol*. Springer; 2019; doi: 10.1186/s13059-019-1891-0.

48. Lu J, Salzberg SL. Removing contaminants from databases of draft genomes. *PLoS Comput Biol*. 2018; doi: 10.1371/journal.pcbi.1006277.

49. Altschul SF, Gish W, Miller W, Myers EW, Lipman DJ. Basic local alignment search tool. *J Mol Biol*. Elsevier; 1990; doi: 10.1016/S0022-2836(05)80360-2.

50. Morgulis A, Coulouris G, Raytselis Y, Madden TL, Agarwala R, Schäffer AA. Database indexing for production MegaBLAST searches. *Bioinformatics*. 2008; doi: 10.1093/bioinformatics/btn322.

51. O'Leary NA, Wright MW, Brister JR, Ciufo S, Haddad D, McVeigh R, et al.. Reference sequence (RefSeq) database at NCBI: current status, taxonomic expansion, and functional annotation. *Nucleic Acids Res*. 2016; doi: 10.1093/nar/gkv1189.

52. Marcais G, Kingford C: Jellyfish: A fast multi-threaded k-mer counter. https://github.com/gmarcais/Jellyfish Accessed 2021 Dec 18.

53. Marçais G, Kingsford C. A fast, lock-free approach for efficient parallel counting of occurrences of k-mers. *Bioinformatics*. academic.oup.com; 2011; doi: 10.1093/bioinformatics/btr011.

54. NCBI: The NCBI eukaryotic genome annotation pipeline. https://www.ncbi.nlm.nih.gov/genome/annotation_euk/process/ Accessed 2021 Dec 18.

55. Morgulis A, Gertz EM, Schäffer AA, Agarwala R. WindowMasker: window-based masker for sequenced genomes. *Bioinformatics*. 2006; doi: 10.1093/bioinformatics/bti774.

56. Pruitt KD, Brown GR, Hiatt SM, Thibaud-Nissen F, Astashyn A, Ermolaeva O, et al.. RefSeq: an update on mammalian reference sequences. *Nucleic Acids Res*. 2014; doi: 10.1093/nar/gkt1114.

57. Kapustin Y, Souvorov A, Tatusova T, Lipman D. Splign: algorithms for computing spliced alignments with identification of paralogs. *Biol Direct*. 2008; doi: 10.1186/1745-6150-3-20.

58. : Gnomon - the NCBI eukaryotic gene prediction tool. https://www.ncbi.nlm.nih.gov/genome/annotation_euk/gnomon/ Accessed 2021 Jan 19.

59. Leinonen R, Sugawara H, Shumway M, International Nucleotide Sequence Database Collaboration. The sequence read archive. *Nucleic Acids Res*. academic.oup.com; 2011; doi: 10.1093/nar/gkq1019.

60. Andrews S: FastQC A quality control tool for high throughput sequence data. https://www.bioinformatics.babraham.ac.uk/projects/fastqc/ Accessed 2021 Dec 18.

61. Andrews S, Others: FastQC: a quality control tool for high throughput sequence data. Babraham Bioinformatics, Babraham Institute, Cambridge, United Kingdom; https://www.bioinformatics.babraham.ac.uk/projects/fastqc/ (2010).

62. Bolger AM, Lohse M, Usadel B: Trimmomatic. https://github.com/usadellab/Trimmomatic Accessed 2021 Dec 18.

63. Bolger AM, Lohse M, Usadel B. Trimmomatic: a flexible trimmer for Illumina sequence data. *Bioinformatics*. academic.oup.com; 2014; doi: 10.1093/bioinformatics/btu170.

64. Song L, Florea L: Rcorrector. https://github.com/mourisl/Rcorrector

65. Song L, Florea L. Rcorrector: efficient and accurate error correction for Illumina RNA-seq reads. *Gigascience*. 2015; doi: 10.1186/s13742-015-0089-y.

66. Haas BJ, Papanicolaou A, Yassour M, Grabherr M, Blood PD, Bowden J, et al.: Trinity. https://github.com/trinityrnaseq/trinityrnaseq Accessed 2021 Dec 18.

67. Haas BJ, Papanicolaou A, Yassour M, Grabherr M, Blood PD, Bowden J, et al.. De novo transcript sequence reconstruction from RNA-seq using the Trinity platform for reference generation and analysis. *Nat Protoc*. nature.com; 2013; doi: 10.1038/nprot.2013.084.

68. Grabherr MG, Haas BJ, Yassour M, Levin JZ, Thompson DA, Amit I, et al.. Full-length transcriptome assembly from RNA-Seq data without a reference genome. *Nat Biotechnol*. ncbi.nlm.nih.gov; 2011; doi: 10.1038/nbt.1883.

69. Haas BJ, Papanicolaou A, Yassour M, Grabherr M, Blood PD, Bowden J, et al.: TransDecoder. https://github.com/TransDecoder/TransDecoder Accessed 2021 Dec 18.

70. Kim J-H, Kim H, Kim H, Chan B, Kang S, Kim W. Draft Genome Assembly of a Fouling Barnacle, Amphibalanus amphitrite (Darwin, 1854): The First Reference Genome for Thecostraca. *Frontiers in Ecology and Evolution*. 2019; doi: 10.3389/fevo.2019.00465.

71. Lee B-Y, Choi B-S, Kim M-S, Park JC, Jeong C-B, Han J, et al.. The genome of the freshwater water flea Daphnia magna: A potential use for freshwater molecular ecotoxicology. *Aquat Toxicol*. 2019; doi: 10.1016/j.aquatox.2019.02.009.

72. Barreto FS, Watson ET, Lima TG, Willett CS, Edmands S, Li W, et al.. Genomic signatures of mitonuclear coevolution across populations of Tigriopus californicus. *Nat Ecol Evol*. 2018; doi: 10.1038/s41559-018-0588-1.

73. Jeong C-B, Lee B-Y, Choi B-S, Kim M-S, Park JC, Kim D-H, et al.. The genome of the harpacticoid copepod Tigriopus japonicus: Potential for its use in marine molecular ecotoxicology. *Aquat Toxicol*. 2020; doi: 10.1016/j.aquatox.2020.105462.

74. Song L, Bian C, Luo Y, Wang L, You X, Li J, et al.. Draft genome of the Chinese mitten crab, Eriocheir sinensis. *Gigascience*. 2016; doi: 10.1186/s13742-016-0112-y.

75. Tang B, Wang Z, Liu Q, Wang Z, Ren Y, Guo H, et al.. Chromosome-level genome assembly of Paralithodes platypus provides insights into evolution and adaptation of king crabs. *Mol Ecol Resour*. 2020; doi: 10.1111/1755-0998.13266.

76. Yang Y, Smith SA. Orthology inference in nonmodel organisms using transcriptomes and

low-coverage genomes: improving accuracy and matrix occupancy for phylogenomics. *Mol Biol Evol*. 2014; doi: 10.1093/molbev/msu245.

77. Fu L, Niu B, Zhu Z, Wu S, Li W: CD-HIT. https://github.com/weizhongli/cdhit Accessed 2021 Dec 18.

78. Fu L, Niu B, Zhu Z, Wu S, Li W. CD-HIT: accelerated for clustering the next-generation sequencing data. *Bioinformatics*. academic.oup.com; 2012; doi: 10.1093/bioinformatics/bts565.

79. Li W. Godzik A. *Cd-hit: a fast program for clustering and comparing large sets of protein or nucleotide sequences Bioinformatics*. 22:1658–92006;

80. van Dongen S: MCL - a cluster algorithm for graphs. http://micans.org/mcl/ Accessed 2021 Dec 18.

81. van Dongen SM. Graph clustering by flow simulation. dspace.library.uu.nl; 2000;

82. Katoh M, Standley DM, Toh H: MAFFT. https://mafft.cbrc.jp/alignment/software/

83. Katoh K, Standley DM. MAFFT multiple sequence alignment software version 7: improvements in performance and usability. *Mol Biol Evol*. academic.oup.com; 2013; doi: 10.1093/molbev/mst010.

84. Smith SA, Dunn CW. Phyutility: a phyloinformatics tool for trees, alignments and molecular data. *Bioinformatics*. 2008; doi: 10.1093/bioinformatics/btm619.

85. Stamatakis A: RAxML. https://github.com/stamatak/standard-RAxML Accessed 2021 Dec 18.

86. Stamatakis A. RAxML version 8: a tool for phylogenetic analysis and post-analysis of large phylogenies. *Bioinformatics*. academic.oup.com; 2014; doi: 10.1093/bioinformatics/btu033.

87. Price MN, Dehal PS, Arkin AP: FastTree. http://www.microbesonline.org/fasttree/

88. Price MN, Dehal PS, Arkin AP. FastTree 2--approximately maximum-likelihood trees for large alignments. *PLoS One*. journals.plos.org; 2010; doi: 10.1371/journal.pone.0009490.

89. Dunn CW, Howison M, Zapata F. Agalma: an automated phylogenomics workflow. *BMC Bioinformatics*. 2013; doi: 10.1186/1471-2105-14-330.

90. Dunn CW, Hejnol A, Matus DQ, Pang K, Browne WE, Smith SA, et al.. Broad phylogenomic sampling improves resolution of the animal tree of life. *Nature*. 2008; doi: 10.1038/nature06614.

91. Talavera G, Castresana J, Kjer K, Page R, Sullivan J: Gblocks. http://molevol.cmima.csic.es/castresana/Gblocks.html Accessed 2021 Dec 18.

92. Talavera G, Castresana J. Improvement of phylogenies after removing divergent and ambiguously aligned blocks from protein sequence alignments. *Syst Biol*. academic.oup.com; 2007; doi: 10.1080/10635150701472164.

93. Minh BQ, Schmidt HA, Chernomor O, Schrempf D, Woodhams, von Haeseler A, et al.: IQ-Tree. http://www.iqtree.org Accessed 2021 Dec 18.

94. Minh BQ, Schmidt HA, Chernomor O, Schrempf D, Woodhams MD, von Haeseler A, et al.. IQ-TREE 2: New Models and Efficient Methods for Phylogenetic Inference in the Genomic Era. *Mol Biol Evol*. 2020; doi: 10.1093/molbev/msaa015.

95. Lanfear R, Frandsen PB, Wright AM, Senfeld T, Calcott B. PartitionFinder 2: New Methods for Selecting Partitioned Models of Evolution for Molecular and Morphological Phylogenetic Analyses. *Mol Biol Evol*. 2017; doi: 10.1093/molbev/msw260.

96. Hoang DT, Chernomor O, von Haeseler A, Minh BQ, Vinh LS. UFBoot2: Improving the Ultrafast Bootstrap Approximation. *Mol Biol Evol*. 2018; doi: 10.1093/molbev/msx281.

97. Lartillot N, Philippe H. A Bayesian mixture model for across-site heterogeneities in the amino-acid replacement process. *Mol Biol Evol*. 2004; doi: 10.1093/molbev/msh112.

98. Lartillot N, Brinkmann H, Philippe H. Suppression of long-branch attraction artefacts in the animal phylogeny using a site-heterogeneous model. *BMC Evol Biol*. 2007; doi: 10.1186/1471-2148-7-S1-S4.

99. Quang LS, Gascuel O, Lartillot N. Empirical profile mixture models for phylogenetic reconstruction. *Bioinformatics*. 2008; doi: 10.1093/bioinformatics/btn445.

100. Wang H-C, Minh BQ, Susko E, Roger AJ. Modeling Site Heterogeneity with Posterior Mean Site Frequency Profiles Accelerates Accurate Phylogenomic Estimation. *Syst Biol*. 2018; doi: 10.1093/sysbio/syx068.

101. Zhang C, Rabiee M, Sayyari E, Mirarab S: ASTRAL: Accurate Species TRee ALgorithm. https://github.com/smirarab/ASTRAL Accessed 2021 Dec 18.

102. Zhang C, Rabiee M, Sayyari E, Mirarab S. ASTRAL-III: polynomial time species tree reconstruction from partially resolved gene trees. *BMC Bioinformatics*. 2018; doi: 10.1186/s12859-018-2129-y.

103. Perina Cedrón A. Analyses of molecular markers and gene expression in crustacean species. ruc.udc.es; 2018;

104. Kim D, Paggi JM, Park D, Bennett C, Salzberg SL: HISAT2. http://daehwankimlab.github.io/hisat2/ Accessed 2021 Dec 18.

105. Kim D, Paggi JM, Park C, Bennett C, Salzberg SL. Graph-based genome alignment and genotyping with HISAT2 and HISAT-genotype. *Nat Biotechnol*. nature.com; 2019; doi: 10.1038/s41587-019-0201-4.

106. Pertea G, Pertea M: gffread: GFF/GTF utility providing format conversions, region filtering, FASTA sequence extraction and more. https://github.com/gpertea/gffread Accessed 2021 Dec 18.

107. Pertea G, Pertea M. GFF Utilities: GffRead and GffCompare. *F1000Res*. 2020; doi: 10.12688/f1000research.23297.2.

108. Liao Y, Smyth GK, Shi W: Subread package: high-performance read alignment, quantification and mutation discovery. http://subread.sourceforge.net Accessed 2021 Dec 18.

109. Liao Y, Smyth GK, Shi W. featureCounts: an efficient general purpose program for

assigning sequence reads to genomic features. *Bioinformatics*. 2014; doi: 10.1093/bioinformatics/btt656.

110. Love MI, Huber W, Anders S: DESeq2. https://bioconductor.org/packages/release/bioc/html/DESeq2.html Accessed 2021 Dec 18.

111. Love MI, Huber W, Anders S. Moderated estimation of fold change and dispersion for RNA-seq data with DESeq2. *Genome Biol*. Springer; 2014; doi: 10.1186/s13059-014-0550-8.

112. Struhl K. Transcriptional noise and the fidelity of initiation by RNA polymerase II. *Nat Struct Mol Biol*. 2007; doi: 10.1038/nsmb0207-103.

113. Pertea M, Shumate A, Pertea G, Varabyou A, Breitwieser FP, Chang Y-C, et al.. CHESS: a new human gene catalog curated from thousands of large-scale RNA sequencing experiments reveals extensive transcriptional noise. *Genome Biol*. 2018; doi: 10.1186/s13059-018-1590-2.

114. Ashburner M, Ball CA, Blake JA, Botstein D, Butler H, Cherry JM, et al.. Gene ontology: tool for the unification of biology. The Gene Ontology Consortium. *Nat Genet*. 2000; doi: 10.1038/75556.

115. Gene Ontology Consortium. The Gene Ontology resource: enriching a GOld mine. *Nucleic Acids Res*. 2021; doi: 10.1093/nar/gkaa1113.

116. Punta M, Coggill PC, Eberhardt RY, Mistry J, Tate J, Boursnell C, et al.. The Pfam protein families database. *Nucleic Acids Res*. 2012; doi: 10.1093/nar/gkr1065.

117. Jones P, Binns D, Chang H-Y, Fraser M, Li W, McAnulla C, et al.: InterProScan. https://interproscan-docs.readthedocs.io/en/latest/ClusterModeBenchmarkRun.html Accessed 2021 Dec 18.

118. Jones P, Binns D, Chang H-Y, Fraser M, Li W, McAnulla C, et al.. InterProScan 5: genome-scale protein function classification. *Bioinformatics*. 2014; doi: 10.1093/bioinformatics/btu031.

119. Alexa A, Rahnenfuhrer J: topGO. https://bioconductor.org/packages/release/bioc/html/topGO.html Accessed 2021 Dec 18.

120. Alexa A, Rahnenführer J. Gene set enrichment analysis with topGO. *Bioconductor Improv*. bioconductor.statistik.tu-dortmund.de; 27:1–262009;

121. Kanehisa M, Goto S. KEGG: kyoto encyclopedia of genes and genomes. *Nucleic Acids Res*. 2000; doi: 10.1093/nar/28.1.27.

122. Aramaki T, Blanc-Mathieu R, Endo H, Ohkubo K, Kanehisa M, Goto S, et al.: KofamKOALA. https://www.genome.jp/tools/kofamkoala/ Accessed 2021 Dec 18.

123. Aramaki T, Blanc-Mathieu R, Endo H, Ohkubo K, Kanehisa M, Goto S, et al.. KofamKOALA: KEGG Ortholog assignment based on profile HMM and adaptive score threshold. *Bioinformatics*. 2020; doi: 10.1093/bioinformatics/btz859.

124. Simão FA, Waterhouse RM, Ioannidis P, Kriventseva EV, Zdobnov EM. BUSCO: assessing genome assembly and annotation completeness with single-copy orthologs. *Bioinformatics*. academic.oup.com; 2015; doi: 10.1093/bioinformatics/btv351.

125. Richards S. Arthropod Genome Sequencing and Assembly Strategies. *Methods Mol Biol*. Springer; 2019; doi: 10.1007/978-1-4939-8775-7_1.

126. Vurture GW, Sedlazeck FJ, Nattestad M, Underwood CJ, Fang H, Gurtowski J, et al.. GenomeScope: fast reference-free genome profiling from short reads. *Bioinformatics*. 2017; doi: 10.1093/bioinformatics/btx153.

127. Kajitani R, Toshimoto K, Noguchi H, Toyoda A, Ogura Y, Okuno M, et al.. Efficient de novo assembly of highly heterozygous genomes from whole-genome shotgun short reads. *Genome Res*. 2014; doi: 10.1101/gr.170720.113.

128. Pérez-Losada M, Høeg JT, Crandall KA. Remarkable convergent evolution in specialized parasitic Thecostraca (Crustacea). *BMC Biol*. 2009; doi: 10.1186/1741-7007-7-15.

129. Ewers-Saucedo C, Owen CL, Pérez-Losada M, Høeg JT, Glenner H, Chan BKK, et al.. Towards a barnacle tree of life: integrating diverse phylogenetic efforts into a comprehensive hypothesis of thecostracan evolution. *PeerJ*. peerj.com; 2019; doi: 10.7717/peerj.7387.

130. Darwin C, Ray society. A monograph on the sub-class Cirripedia. the Ray Society;

131. Haeckel E. The Wonders of Life: A Popular Study of Biological Philosophy. Harper & brothers;

132. Niiyama H. A comparative study of the chromosomes in decapods, isopods and amphipods, with some remarks on cytotaxonomy and sex-determination in the Crustacea. *Mem Fac Fish Hokkaido Univ*. THE FACULTY OF FISHERIES, HOKKAIDO UNIVERSITY; 7:1–601959;

133. KORN, O. M. Naupliar evidence for Cirripede taxonomy and phylogeny. *New Frontiers in Barnacle Evolution*. A. A. Balkema; :87–1211995;

134. Newman WA, Ross A. Prospectus on Larval Cirriped Setation Formulae, Revisited. *J Crustacean Biol*. Oxford Academic; 2001; doi: 10.1163/20021975-99990109.

135. Lang WH. Larval Development of Shallow Water Barnacles of the Carolinas (Cirripedia; Thoracica) with Keys to Naupliar Stages. Department of Commerce, National Oceanic and Atmospheric Administration, National Marine Fisheries Service;

136. Korn OM, Kulikova VA. Seasonal species composition and distribution of barnacle larvae in Avacha Inlet (Kamchatka). *J Plankton Res*. Oxford Academic; 1995; doi: 10.1093/plankt/17.2.221.

137. Burrows MT, Hawkins SJ, Southward AJ. Larval development of the intertidal barnacles Chthamalus stellatus and Chthamalus montagui. *J Mar Biol Assoc U K*. Cambridge University Press; 1999; doi: 10.1017/S0025315498000101.

138. Dreyer N, Tsai P-C, Olesen J, Kolbasov GA, Høeg JT, Chan BKK. Independent and adaptive evolution of phenotypic novelties driven by coral symbiosis in barnacle larvae. *Evolution*. 2021; doi: 10.1111/evo.14380.

139. Franco SC, Aldred N, Cruz T, Clare AS. Effects of culture conditions on larval growth and survival of stalked barnacles (Pollicipes pollicipes). *Aquac Res*. Wiley; 2017; doi: 10.1111/are.13125.

140. Nishiguchi S, Yagi A, Sakai N, Oda H. Divergence of structural strategies for homophilic E-cadherin binding among bilaterians. *J Cell Sci*. 2016; doi: 10.1242/jcs.189258.

141. Lewis CA. Juvenile To Adult Shift in Feeding Strategies in the Pedunculate Barnacle Pollicipes Polymerus (Sowerby) (Cirripedia, Lepadomorpha). *Crustaceana*. Brill; 1981; doi: 10.1163/156854081X00039.

142. Statello L, Guo C-J, Chen L-L, Huarte M. Gene regulation by long non-coding RNAs and its biological functions. *Nat Rev Mol Cell Biol*. 2021; doi: 10.1038/s41580-020-00315-9.

143. Koutsos AC, Blass C, Meister S, Schmidt S, MacCallum RM, Soares MB, et al.. Life cycle transcriptome of the malaria mosquito Anopheles gambiae and comparison with the fruitfly Drosophila melanogaster. *Proc Natl Acad Sci U S A*. National Acad Sciences; 2007; doi: 10.1073/pnas.0703988104.

144. Kim WJ, Lee SH, An SB, Kim SE, Liu Q, Choi JY, et al.. Comparative Transcriptome Analysis of Queen, Worker, and Larva of Asian Honeybee, Apis cerana. *Int J Indust Entomol*. Korean Society of Sericultural Science; 2013; doi: 10.7852/ijie.2013.27.2.271.

145. Graveley BR, Brooks AN, Carlson JW, Duff MO, Landolin JM, Yang L, et al.. The developmental transcriptome of Drosophila melanogaster. *Nature*. Nature Publishing Group; 2010; doi: 10.1038/nature09715.

146. Yang H, Cai Y, Zhuo Z, Yang W, Yang C, Zhang J, et al.. Transcriptome analysis in different developmental stages of Batocera horsfieldi (Coleoptera: Cerambycidae) and comparison of candidate olfactory genes. *PLoS One*. 2018; doi: 10.1371/journal.pone.0192730.

147. Allen ML, Rhoades JH, Sparks ME, Grodowitz MJ. Differential Gene Expression in Red Imported Fire Ant (Solenopsis invicta) (Hymenoptera: Formicidae) Larval and Pupal Stages. *Insects*. 2018; doi: 10.3390/insects9040185.

148. Noriega DD, Arias PL, Barbosa HR, Arraes FBM, Ossa GA, Villegas B, et al.. Transcriptome and gene expression analysis of three developmental stages of the coffee berry borer, Hypothenemus hampei. *Sci Rep*. 2019; doi: 10.1038/s41598-019-49178-x.

149. Takele Assefa A, Vandesompele J, Thas O. On the utility of RNA sample pooling to optimize cost and statistical power in RNA sequencing experiments. *BMC Genomics*. 2020; doi: 10.1186/s12864-020-6721-y.

150. Machado AM, Sarropoulou E, Castro LFC, Vasconcelos V, Cunha I. An important resource for understanding bio-adhesion mechanisms: Cement gland transcriptomes of two goose barnacles, Pollicipes pollicipes and Lepas anatifera (Cirripedia, Thoracica). *Mar Genomics*. Elsevier; 2019; doi: 10.1016/j.margen.2018.11.001.

Figure1

Click here to access/download;Figure;Figure1.jpg

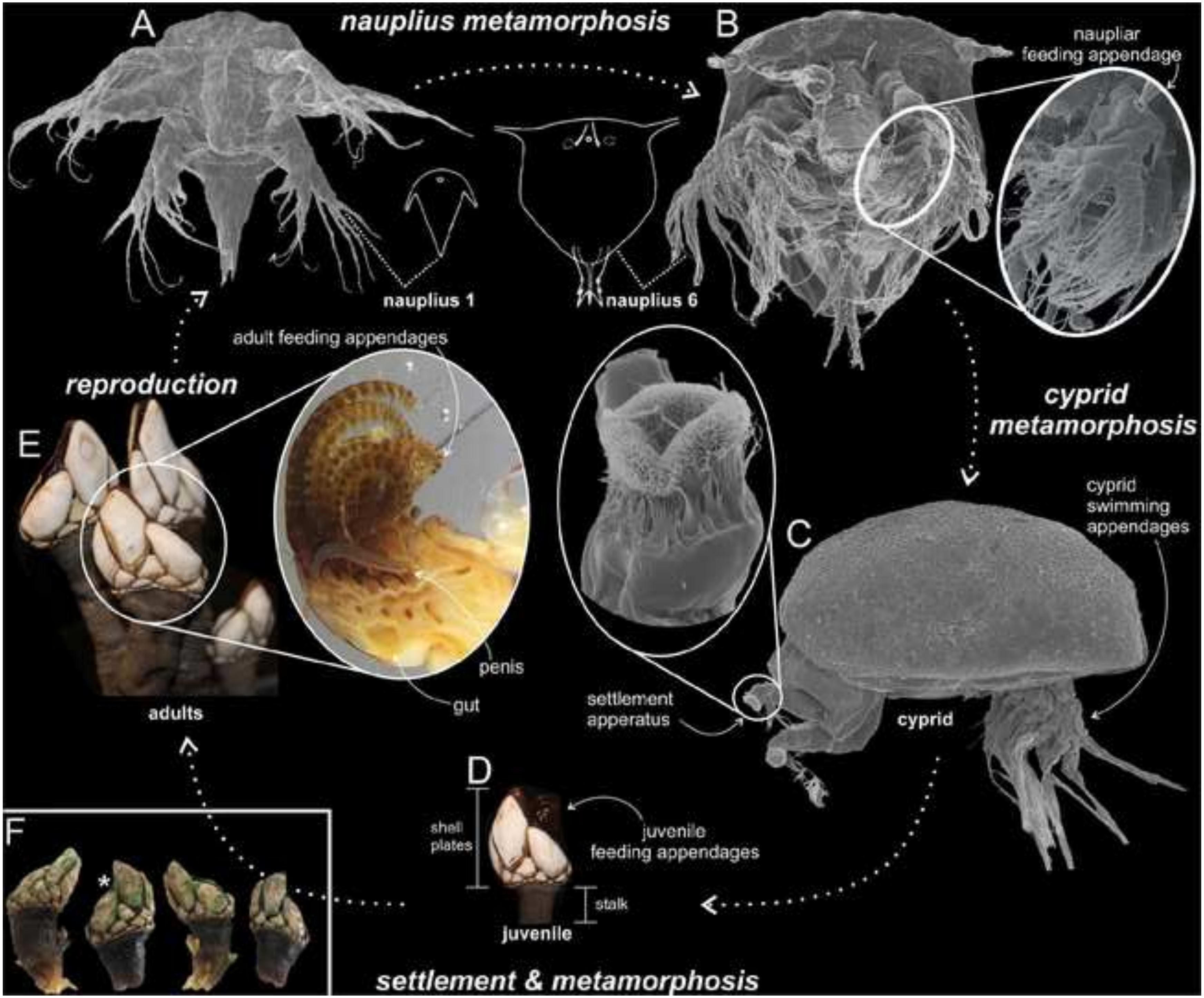

Figure2 Click here to access/download;Figure;Figure2.jpg

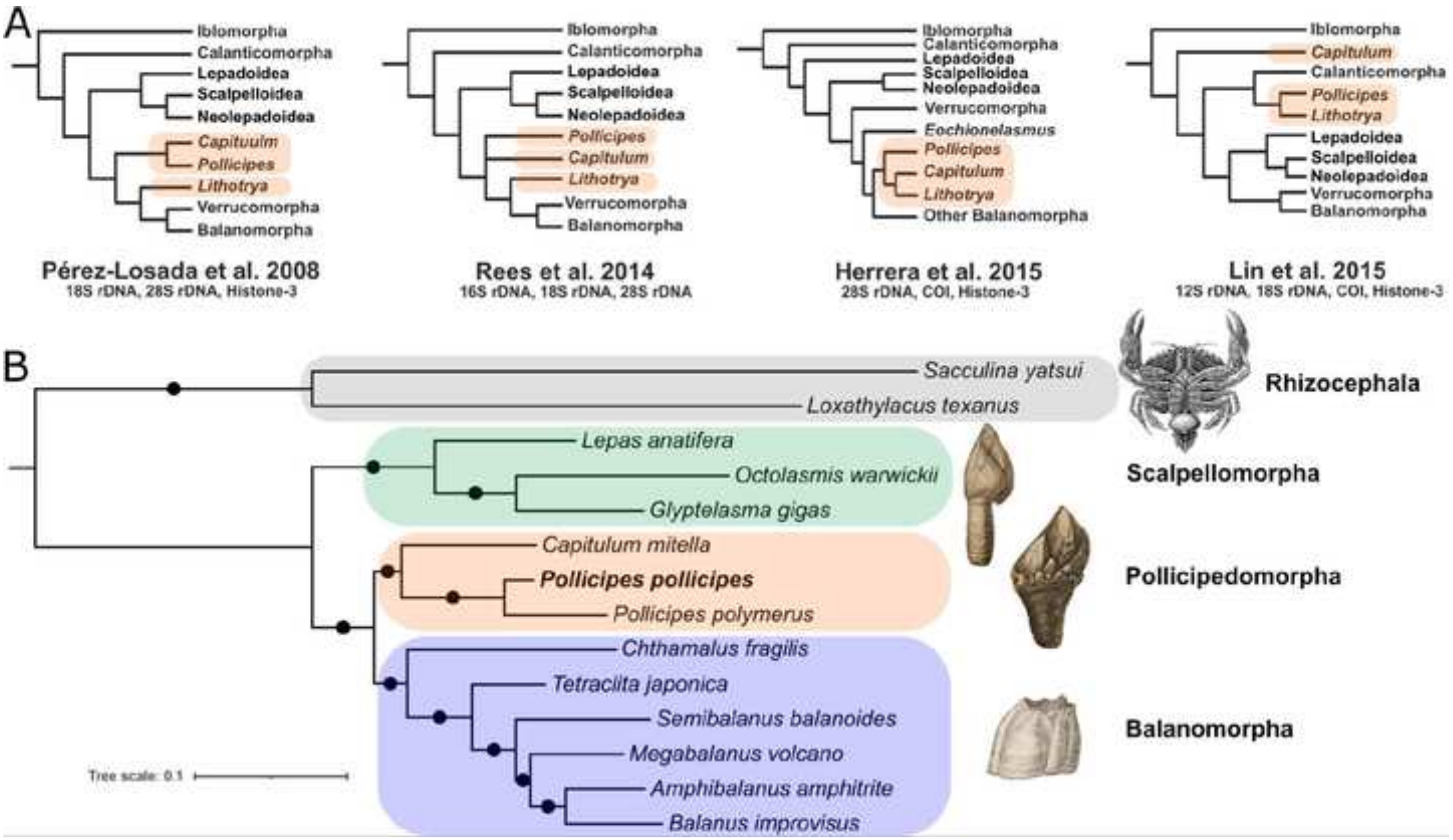

Figure3 Click here to access/download;Figure;Figure3.jpg

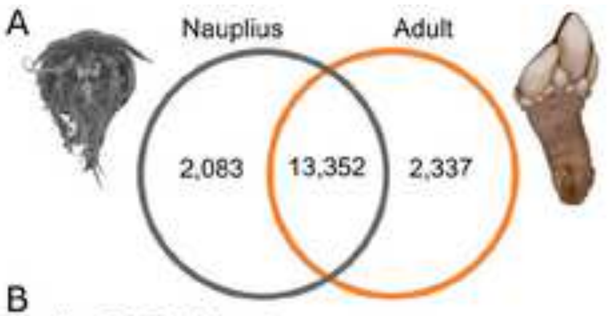

B

Nauplius GO Enrichment

Gene Ontology and name	GO term	q value
Molecular Function		
endopeptidase activity	GO:0004175	2.0E-22
serine-type endopeptidase activity	GO:0004252	1.3E-18
calcium ion binding	GO:0005509	2.5E-11
minus-end-directed microtubule motor activity	GO:0008569	5.8E-10
chitin binding	GO:0008061	1.3E-05
Biological Process		
proteolysis	GO:0006508	8.8E-17
homophilic cell adhesion via plasma membrane adhesion molecules	GO:0007156	6.3E-08
microtubule based movement	GO:0007018	1.3E-04
Cellular Component		
extracellular region	GO:0005576	5.5E-08
dynein complex	GO:0030286	4.6E-06
endoplasmic reticulum	GO:0005783	2.0E-02

C

Adult GO Enrichment

Gene Ontology and name	GO term	q value
Molecular Function		
structural constituent of cuticle	GO:0042302	<1.0E-23
iron ion binding	GO:0005506	1.1E-23
heme binding	GO:0020037	3.2E-18
oxidoreductase activity acting on paired donors with incorporation or reduction of molecular oxygen	GO:0016705	1.6E-16
chitin binding	GO:0008061	1.6E-15
hydrolase activity acting on carbon-nitrogen but not peptide bonds	GO:0016810	3.0E-07
Biological Process		
oxidation-reduction process	GO:0055114	2.2E-18
transmembrane transport	GO:0055085	1.9E-07
sodium ion transport	GO:0006814	3.7E-05
anion transport	GO:0006820	1.2E-04
Cellular Component		
membrane	GO:0016020	5.6E-07
extracellular region	GO:0005576	1.8E-05
integral component of membrane	GO:0016021	5.8E-04
intrinsic component of membrane	GO:0031224	5.8E-04
collagen trimer	GO:0005581	9.8E-03

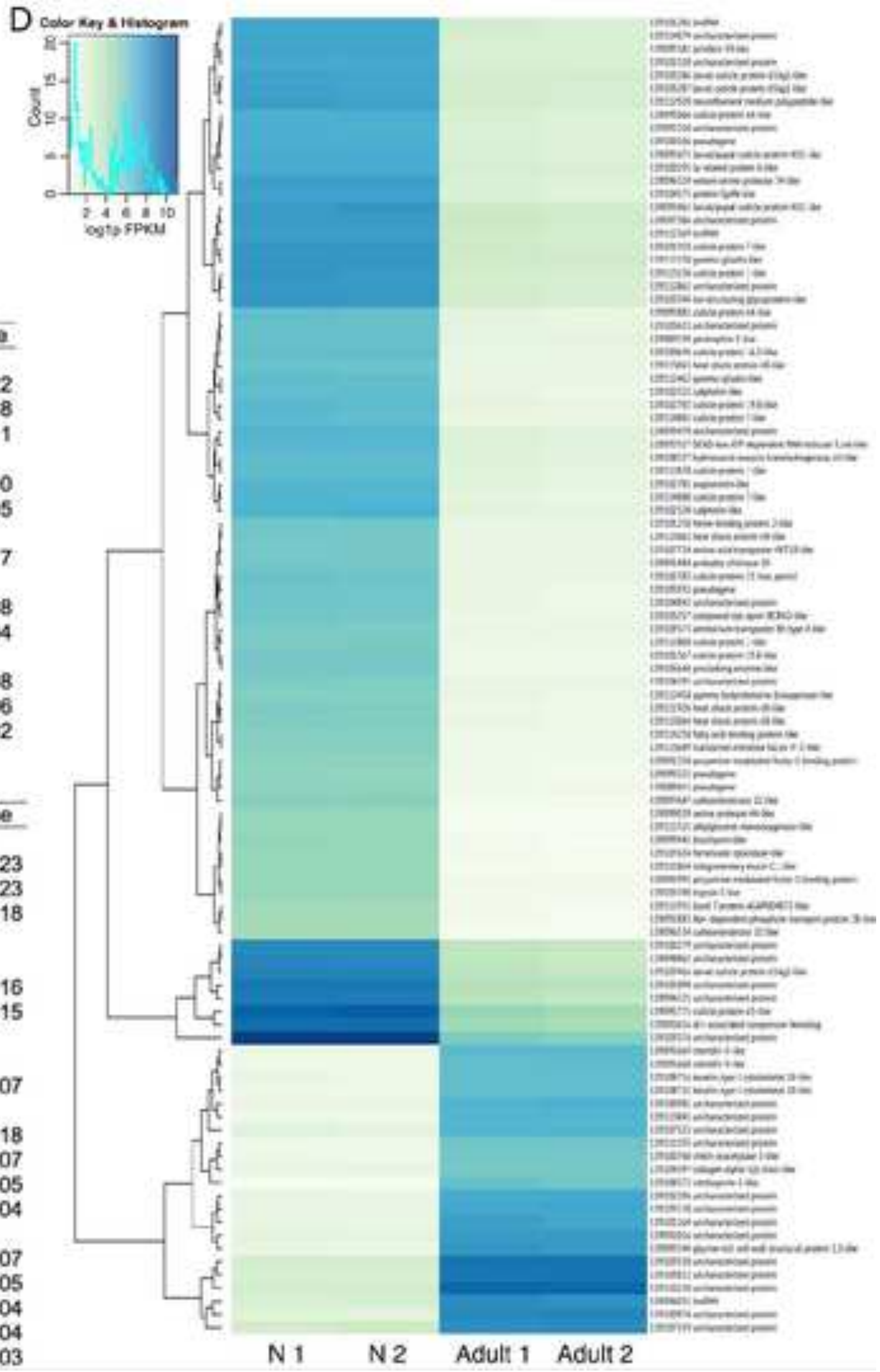

Click here to access/download
Supplementary Material
Supplemental Information.docx

Reviewer Response Click here to access/download;Personal Cover;PollicipesResponse.docx

Reviewer comments followed by **author's response in bold**.

<u>Reviewer #1:</u>
This study entitled 'Chromosome-level genome assembly, annotation, and phylogenomics of the gooseneck barnacle Pollicipes pollicipes' has reported a relative chromosome-level barnacle genome by using the Illumina, Pacbio and Hic sequencing. The scaffold N50 and contig N50 is 47M and 95.5k. Comparative transcriptome analysis between nauplii and adults were performed in this study. DEG results have shown that some genes such as vision genes were related to the morphological changes in these two stages. GO and KEGG enrichment also discovered some genes and pathways were involved in tissue modeling. Phylogenetic analysis was also performed in this study to provide a more detailed phylogenetic placement in some representative species. In summary, this study reported a relatively good assembly and some analyses. This species can also attract wide interests of researchers.

However, the major weakness of this study is that the analysis points are really sparse. I suggest the authors to combine the DEGs and find their detailed relationships to be a pathway in a figure.
This is challenging given how little is known about barnacle molecular biology and pathways in barnacles. For example, only 51% of genes could be assigned GO terms, while 82% were assigned KEGG orthologs. We also assigned the DEGs to KEGG pathways. However, the KEGG ortholog assignments tended to be very specific (e.g., "dynein heavy chain 1, cytosolic" Table S7, S8) and the KEGG pathways were very broad (e.g., "metabolism" or "signal transduction"), while GO terms varied from very specific to very broad. We attempted to summarize these results into something biologically meaningful and interpretable (not too specific while not too general) with KEGG Pathway analysis and the GO enrichment analyses in Figs. S9-S14. Given the issues with very narrow and very broad annotations that occur when summarizing thousands of genes, we found the TopGO enrichment analysis and nesting of significant terms in Figs. S9-S14 and Figure 3B, C to be a good balance.

The English language of this paper should be largely polished.
We made a number of minor changes for clarity throughout the text and polished our writing. We would be happy to address specific areas of difficulty.

From the second paragraph of Introduction, what's meaning of 'Thecostraca' and 'Thecostracans '?
These are the scientific names of the Linnaean taxa to which barnacles belong. Cirripedia = barnacles, while Thecostraca = Cirripedia + Ascothoracida + Rhizocephala + Facetotecta. We added explanations about these higher level taxa to the Introduction.

Decimal places should be uniformed through whole paper, like 26.59%, 904.6 and 189.06, etc.
Change made to a single decimal place.

For the filtering step of contaminants, what's ratio of the contaminated scaffold in whole assembly?
62 of the 576 scaffolds were identified as bacterial contaminants, but all 62 contaminant scaffolds were small scaffolds (the largest contaminated scaffold was 99 KB). Collectively, these 62 scaffolds comprised 7.2MB (including repetitive regions), <1% of the final assembly length. We added these numbers to the text of the results.

In figure 3, the table and figures should be shown independently.
While not a typical figure arrangement, we added the tables here for space efficiency because the heatmap requires a rather tall figure. We would prefer to keep them together, so there is not wasted space to the left and right of figure 3. But we are happy to defer to the publisher on this point and can make adjustments as needed.

Word font in Phylogenomics should be revised.
Change made.

For the format of this paper:
The abstract should be divided into three parts of Background, Results and Conclusion.
Change made.

The format of Tables should be revised.
Some of the table sizing is cumbersome because they are inserted into the manuscript text and are constrained to 8.5 x 11 page sizing. We will work with the journal to properly format the tables depending on the constraints on their dimensions and font sizes in the final display formatting.

The authors should provide the RRID and version of each used software should be added in paper.
Version numbers added throughout the manuscript and software citations added following GigaScience formatting from https://f1000research.com/articles/9-1257/v2#FN1

Reviewer #2:

The paper entitled "Chromosome-level genome assembly and annotation of the gooseneck barnacle Pollicipes pollicipes" by Bernot et al. presents a high-quality genome assembly of a barnacle (770 Mbp, 16-17 chromosomes, 90.5% BUSCO), which was obtained using a state-of-the art pipeline including PacBio raw sequence data, Illumina reads to polish indels, and DOVETAIL Chicago and HiC libraries to capture chromatin information. In addition, the authors sequenced and compared two transcriptomes each of larvae and adults of the species, finding striking differences in gene expression. Phylogenomic analyses were performed that placed Pollicipedomorpha sister to Balanomorpha. Altogether, I find this study a very valuable contribution that will be important for future comparative genomic studies within Thecostraca and will be of the interest of the wider readership of Gigascience.

I have only few minor comments that the authors may want to consider when revising the paper:

-Larval culturing and morphology. It is really nice that the authors were able to hatch eggs and rear nauplii, cyprids and adults of this barnacle species (as shown in Fig. 1). A detailed morphological study is mentioned, but only in the M&M section. Although I understand that the genome is the focus of the paper, do not morphology and development deserve some lines in the results and discussion sections?

Thank you, we added a larval culturing and morphology paragraph to the Results section.

-Differential gene expression. I assume that the authors could ONLY have two biological replicates per larval and adult stage. However, as they may be aware, it is normally recommended to have a minimum of three replicates for statistical purposes. In this regard, they should tone down (e.g., extreme differences?) the interpretation of their results (or at least mention the limitations of the comparisons).

The Reviewer is correct. We rephrased the "extreme differences" to "substantial differences" and added sentences to the end of the differential gene expression Discussion paragraph to point out the replication limitations.

-Genome annotation. The authors mention in M&M that they used WindowMasker but in the results they mention that first they tried RepeatMasker. The use of the latter should be mentioned in M&M.

Change made.

In any case, they assume that they had low recovery of repeat elements due to the lack of a repeat library for barnacles. In this case, they should use RepeatModeler (before RepatMasker) and create de novo a reference library directly from the genome.

Repeat masking was done as part of the NCBI annotation pipeline, and they found WindowMasker worked well to mask repeats. It was used to mask 18.5% of the genome as repetitive before downstream annotation.

-References: I think that they should add more references (in addition to [7]) to support the importance of barnacles as model system. The references of previous phylogenetic hypotheses (Lin et al, 2015, Rees et al 2014, Herrera et al, 2015) should be included in the reference list. The studies that generated the transcriptomes should be credited (e.g., Machado et al. 2019. An important resource for understanding bio-adhesion mechanisms: Cement gland transcriptomes of two goose barnacles, Pollicipes pollicipes and Lepas anatifera (Cirripedia, Thoracica). Marine Genomics, 45, 16-20). **Changes made. Added these citations and others to the reference list.**

Check reference style of Gigascience. I miss the year in all references.
The years were present but incorrectly formatted after the page numbers. We have fixed the citations and they are now in the GigaScience format. We actually used Paperpile to format the references selecting the GigaScience format. Apparently, Paperpile has now updated this format. We now have used the updated version.

-Table 1. Illumina WGS. 150,600 millions divided by 770 Mb equals 195.58x. On the other hand, 770 Mb times 189.06x equals 145,576.200 millions. Please check all numbers (in the text 1,043.2 and 904.6 million reads are mentioned).
Thank you for pointing out this mistake in Table 1. The 189x coverage in the table was after QA/QC. The table presents raw sequence data so you are correct that it should be 196x coverage in Table 1. We revised the numbers in Table 1 and the text to reflect this.
