# Supplementary material for: Chromosome-level genome assembly, annotation, and phylogenomics of the gooseneck barnacle Pollicipes pollicipes
Source: Gigascience. 2022 Mar 12;11:giac021. doi: 10.1093/gigascience/giac021 (PMC8917513; doi:10.1093/gigascience/giac021)
Supplement: giac021_Response_to_Reviewer_Comments_Revision_2 [file giac021_response_to_reviewer_comments_revision_2.pdf]

Author's Response To Reviewer Comments

Close

Please see attached document for the authors response to concerns.

Close
