# Supplementary material for: Chromosome-level genome assembly, annotation, and phylogenomics of the gooseneck barnacle Pollicipes pollicipes
Source: Gigascience. 2022 Mar 12;11:giac021. doi: 10.1093/gigascience/giac021 (PMC8917513; doi:10.1093/gigascience/giac021)
Supplement: giac021_Reviewer_1_Report_Original_Submission — Chao Bian -- 11/23/2021 Reviewed [file giac021_reviewer_1_report_original_submission.pdf]

**Reviewer Report**

**Title: Chromosome-level genome assembly, annotation, and phylogenomics of the gooseneck barnacle Pollicipes pollicipes**

**Version: Original Submission** **Date:** 11/23/2021

**Reviewer name: Chao Bian**

**Reviewer Comments to Author:**

This study entitled 'Chromosome-level genome assembly, annotation, and phylogenomics of the gooseneck barnacle Pollicipes pollicipes' has reported a relative chromosome-level barnacle genome by using the Illumina, Pacbio and Hic sequencing. The scaffold N50 and contig N50 is 47M and 95.5k. Comparative transcriptome analysis between nauplii and adults were performed in this study. DEG results have shown that some genes such as vision genes were related to the morphological changes in these two stages. GO and KEGG enrichment also discovered some genes and pathways were involved in tissue modeling. Phylogenetic analysis was also performed in this study to provide a more detailed phylogenetic placement in some representative species. In summary, this study reported a relatively good assembly and some analyses. This species can also attract wide interests of researchers.
However, the major weakness of this study is that the analysis points are really sparse. I suggest the authors to combine the DEGs and find their detailed relationships to be a pathway in a figure.
The English language of this paper should be largely polished.
From the second paragraph of Introduction, what's meaning of 'Thecostraca' and 'Thecostracans '?
Decimal places should be uniformed through whole paper, like 26.59%, 904.6 and 189.06, etc.
For the filtering step of contaminants, what's ratio of the contaminated scaffold in whole assembly?
In figure 3, the table and figures should be shown independently.
Word font in Phylogenomics should be revised.
For the format of this paper:
The abstract should be divided into three parts of Background, Results and Conclusion.
The format of Tables should be revised.
The authors should provide the RRID and version of each used software should be added in paper.

**Level of Interest**

Please indicate how interesting you found the manuscript: Choose an item.

**Quality of Written English**

Please indicate the quality of language in the manuscript: Choose an item.

**Declaration of Competing Interests**

Please complete a declaration of competing interests, considering the following questions:

- Have you in the past five years received reimbursements, fees, funding, or salary from an organisation that may in any way gain or lose financially from the publication of this manuscript, either now or in the future?
- Do you hold any stocks or shares in an organisation that may in any way gain or lose financially from the publication of this manuscript, either now or in the future?
- Do you hold or are you currently applying for any patents relating to the content of the manuscript?
- Have you received reimbursements, fees, funding, or salary from an organization that holds or has applied for patents relating to the content of the manuscript?
- Do you have any other financial competing interests?
- Do you have any non-financial competing interests in relation to this paper?

If you can answer no to all of the above, write 'I declare that I have no competing interests' below. If your reply is yes to any, please give details below.

I declare that I have no competing interests

I agree to the open peer review policy of the journal. I understand that my name will be included on my report to the authors and, if the manuscript is accepted for publication, my named report including any attachments I upload will be posted on the website along with the authors' responses. I agree for my report to be made available under an Open Access Creative Commons CC-BY license (http://creativecommons.org/licenses/by/4.0/). I understand that any comments which I do not wish to be included in my named report can be included as confidential comments to the editors, which will not be published.

Choose an item.

To further support our reviewers, we have joined with Publons, where you can gain additional credit to further highlight your hard work (see: https://publons.com/journal/530/gigascience). On publication of this paper, your review will be automatically added to Publons, you can then choose whether or not to claim your Publons credit. I understand this statement.

Yes Choose an item.
