# Supplementary material for: Chromosome-level genome assembly, annotation, and phylogenomics of the gooseneck barnacle Pollicipes pollicipes
Source: Gigascience. 2022 Mar 12;11:giac021. doi: 10.1093/gigascience/giac021 (PMC8917513; doi:10.1093/gigascience/giac021)
Supplement: giac021_Reviewer_1_Report_Revision_1 — Chao Bian -- 1/18/2022 Reviewed [file giac021_reviewer_1_report_revision_1.pdf]

**Reviewer Report**

**Title: Chromosome-level genome assembly, annotation, and phylogenomics of the gooseneck barnacle Pollicipes pollicipes**

**Version: Revision 1** **Date:** 1/18/2022

**Reviewer name: Chao Bian**

**Reviewer Comments to Author:**

This study entitled 'Chromosome-level genome assembly, annotation, and phylogenomics of the gooseneck barnacle Pollicipes pollicipes' reported a very fascinated species, gooseneck barnacle. A large number of genome data were sequenced, and were then assembled to be a chromosome-level assembly. BUSCO evaluation value is also relatively high. The major goal of this study is to provide a confirmed phylogenetic tree and show some gene changes in two different stages. The morphological introduction in discussion, in my opinion, is absolutely important and punch lines for this study, which is very good for readers to understand this interested species.
However, the point statements are clear but only have some common results produced by several general pipelines, like KEGG, GO enrichments, DEG identification, etc. A representative and comprehensive pathway should be initiated in this study.
The English language of this study is indeed well written.
This study is really good for gigascience publication, if the authors well addressed some defects,
Some questions:
How about the estimated genome size of this species by using the kmer analysis?
Suggest to show the HIC heatmap in this study, because this heatmap can comprehensively and directly exhibit the quality of your assembly with chromosome level.
Some minor suggestions:
The decimal places should be uniformed in this study.
For the second paragraph, what's meaning of Thecostraca and thecostacans?
'with a rich fossil record' suggests to be 'with rich fossil records'.
Near ref [20] 'occupies' should be 'occupying'.
'FALCON [30] V1.8.8 [31]' should be 'FALCON V1.8.8 [30, 31]'.
The RRIDs of used software should be demonstrated in this study.
'RepeatMasker identified 3.2% of the genome as repetitive' revised to 'RepeatMasker identified 3.2% of the genome as repetitive sequences'.
The format of Table 3 and 4 should be revised.

**Level of Interest**

Please indicate how interesting you found the manuscript: Choose an item.

**Quality of Written English**

Please indicate the quality of language in the manuscript: Choose an item.

**Declaration of Competing Interests**

Please complete a declaration of competing interests, considering the following questions:

- Have you in the past five years received reimbursements, fees, funding, or salary from an organisation that may in any way gain or lose financially from the publication of this manuscript, either now or in the future?
- Do you hold any stocks or shares in an organisation that may in any way gain or lose financially from the publication of this manuscript, either now or in the future?
- Do you hold or are you currently applying for any patents relating to the content of the manuscript?
- Have you received reimbursements, fees, funding, or salary from an organization that holds or has applied for patents relating to the content of the manuscript?
- Do you have any other financial competing interests?
- Do you have any non-financial competing interests in relation to this paper?

If you can answer no to all of the above, write 'I declare that I have no competing interests' below. If your reply is yes to any, please give details below.

I declare that I have no competing interests

I agree to the open peer review policy of the journal. I understand that my name will be included on my report to the authors and, if the manuscript is accepted for publication, my named report including any attachments I upload will be posted on the website along with the authors' responses. I agree for my report to be made available under an Open Access Creative Commons CC-BY license (http://creativecommons.org/licenses/by/4.0/). I understand that any comments which I do not wish to be included in my named report can be included as confidential comments to the editors, which will not be published.

Choose an item.

To further support our reviewers, we have joined with Publons, where you can gain additional credit to further highlight your hard work (see: https://publons.com/journal/530/gigascience). On publication of this paper, your review will be automatically added to Publons, you can then choose whether or not to claim your Publons credit. I understand this statement.

Yes Choose an item.
