# Supplementary material for: Chromosome-level genome assembly, annotation, and phylogenomics of the gooseneck barnacle Pollicipes pollicipes
Source: Gigascience. 2022 Mar 12;11:giac021. doi: 10.1093/gigascience/giac021 (PMC8917513; doi:10.1093/gigascience/giac021)
Supplement: giac021_Reviewer_2_Report_Original_Submission — Rafael Zardoya, PhD -- 12/7/2021 Reviewed [file giac021_reviewer_2_report_original_submission.pdf]

Reviewer Report

Title: Chromosome-level genome assembly, annotation, and phylogenomics of the gooseneck barnacle Pollicipes pollicipes

Version: Original Submission **Date:** 12/7/2021

Reviewer name: Rafael Zardoya, PhD

Reviewer Comments to Author:

The paper entitled "Chromosome-level genome assembly and annotation of the gooseneck barnacle Pollicipes pollicipes" by Bernot et al. presents a high-quality genome assembly of a barnacle (770 Mbp, 16-17 chromosomes, 90.5% BUSCO), which was obtained using a state-of-the art pipeline including PacBio raw sequence data, Illumina reads to polish indels, and DOVETAIL Chicago and HiC libraries to capture chromatin information. In addition, the authors sequenced and compared two transcriptomes each of larvae and adults of the species, finding striking differences in gene expression. Phylogenomic analyses were performed that placed Pollicipedomorpha sister to Balanomorpha. Altogether, I find this study a very valuable contribution that will be important for future comparative genomic studies within Thecostraca and will be of the interest of the wider readership of Gigascience.

I have only few minor comments that the authors may want to consider when revising the paper:

- Larval culturing and morphology. It is really nice that the authors were able to hatch eggs and rear nauplii, cyprids and adults of this barnacle species (as shown in Fig. 1). A detailed morphological study is mentioned, but only in the M&M section. Although I understand that the genome is the focus of the paper, do not morphology and development deserve some lines in the results and discussion sections?
- Differential gene expression. I assume that the authors could ONLY have two biological replicates per larval and adult stage. However, as they may be aware, it is normally recommended to have a minimum of three replicates for statistical purposes. In this regard, they should tone down (e.g., extreme differences?) the interpretation of their results (or at least mention the limitations of the comparisons).
- Genome annotation. The authors mention in M&M that they used WindowMasker but in the results they mention that first they tried RepeatMasker. The use of the latter should be mentioned in M&M. In any case, they assume that they had low recovery of repeat elements due to the lack of a repeat library for barnacles. In this case, they should use RepeatModeler (before RepatMasker) and create de novo a reference library directly from the genome.
- References: I think that they should add more references (in addition to [7]) to support the importance of barnacles as model system. The references of previous phylogenetic hypotheses (Lin et al, 2015, Rees et al 2014, Herrera et al, 2015) should be included in the reference list. The studies that generated the transcriptomes should be credited (e.g., Machado et al. 2019. An important resource for understanding bio-adhesion mechanisms: Cement gland transcriptomes of two goose barnacles, Pollicipes pollicipes and Lepas anatifera (Cirripedia, Thoracica). Marine Genomics, 45, 16-20). Check reference style of Gigascience. I miss the year in all references.
- Table 1. Illumina WGS. 150,600 millions divided by 770 Mb equals 195.58x. On the other hand, 770 Mb

times 189.06x equals 145,576.200 millions. Please check all numbers (in the text 1,043.2 and 904.6 million reads are mentioned).

Level of Interest

Please indicate how interesting you found the manuscript: Choose an item.

Quality of Written English

Please indicate the quality of language in the manuscript: Choose an item.

Declaration of Competing Interests

Please complete a declaration of competing interests, considering the following questions:

- Have you in the past five years received reimbursements, fees, funding, or salary from an organisation that may in any way gain or lose financially from the publication of this manuscript, either now or in the future?
- Do you hold any stocks or shares in an organisation that may in any way gain or lose financially from the publication of this manuscript, either now or in the future?
- Do you hold or are you currently applying for any patents relating to the content of the manuscript?
- Have you received reimbursements, fees, funding, or salary from an organization that holds or has applied for patents relating to the content of the manuscript?
- Do you have any other financial competing interests?
- Do you have any non-financial competing interests in relation to this paper?

If you can answer no to all of the above, write 'I declare that I have no competing interests' below. If your reply is yes to any, please give details below.

I declare I have no competing interests

I agree to the open peer review policy of the journal. I understand that my name will be included on my report to the authors and, if the manuscript is accepted for publication, my named report including any attachments I upload will be posted on the website along with the authors' responses. I agree for my report to be made available under an Open Access Creative Commons CC-BY license (http://creativecommons.org/licenses/by/4.0/). I understand that any comments which I do not wish to be included in my named report can be included as confidential comments to the editors, which will not be published.

Choose an item.

To further support our reviewers, we have joined with Publons, where you can gain additional credit to further highlight your hard work (see: https://publons.com/journal/530/gigascience). On publication of this paper, your review will be automatically added to Publons, you can then choose whether or not to claim your Publons credit. I understand this statement.

Yes Choose an item.
